# Cardiovascular risk prediction in type 2 diabetes: a comparison of 22 risk scores in primary care settings

**DOI:** 10.1007/s00125-021-05640-y

**Published:** 2022-01-15

**Authors:** Katarzyna Dziopa, Folkert W. Asselbergs, Jasmine Gratton, Nishi Chaturvedi, Amand F. Schmidt

**Affiliations:** 1 Health Data Research UK and Institute of Health Informatics, University College London, London, UK; 2 Institute of Cardiovascular Science, Faculty of Population Health Sciences, University College London, London, UK; 3 Department of Cardiology, Division Heart and Lungs, University Medical Centre Utrecht, Utrecht University, Utrecht, the Netherlands; 4 MRC Unit for Lifelong Health and Ageing at UCL, University College London, London, UK

**Keywords:** Cardiovascular disease, Diabetes, Prediction, Risk score

## Abstract

**Aims/hypothesis:**

We aimed to compare the performance of risk prediction scores for CVD (i.e., coronary heart disease and stroke), and a broader definition of CVD including atrial fibrillation and heart failure (CVD+), in individuals with type 2 diabetes.

**Methods:**

Scores were identified through a literature review and were included irrespective of the type of predicted cardiovascular outcome or the inclusion of individuals with type 2 diabetes. Performance was assessed in a contemporary, representative sample of 168,871 UK-based individuals with type 2 diabetes (age ≥18 years without pre-existing CVD+). Missing observations were addressed using multiple imputation.

**Results:**

We evaluated 22 scores: 13 derived in the general population and nine in individuals with type 2 diabetes. The Systemic Coronary Risk Evaluation (SCORE) CVD rule derived in the general population performed best for both CVD (C statistic 0.67 [95% CI 0.67, 0.67]) and CVD+ (C statistic 0.69 [95% CI 0.69, 0.70]). The C statistic of the remaining scores ranged from 0.62 to 0.67 for CVD, and from 0.64 to 0.69 for CVD+. Calibration slopes (1 indicates perfect calibration) ranged from 0.38 (95% CI 0.37, 0.39) to 0.74 (95% CI 0.72, 0.76) for CVD, and from 0.41 (95% CI 0.40, 0.42) to 0.88 (95% CI 0.86, 0.90) for CVD+. A simple recalibration process considerably improved the performance of the scores, with calibration slopes now ranging between 0.96 and 1.04 for CVD. Scores with more predictors did not outperform scores with fewer predictors: for CVD+, QRISK3 (19 variables) had a C statistic of 0.68 (95% CI 0.68, 0.69), compared with SCORE CVD (six variables) which had a C statistic of 0.69 (95% CI 0.69, 0.70). Scores specific to individuals with diabetes did not discriminate better than scores derived in the general population: the UK Prospective Diabetes Study (UKPDS) scores performed significantly worse than SCORE CVD (p value <0.001).

**Conclusions/interpretation:**

CVD risk prediction scores could not accurately identify individuals with type 2 diabetes who experienced a CVD event in the 10 years of follow-up. All 22 evaluated models had a comparable and modest discriminative ability.

**Graphical abstract:**

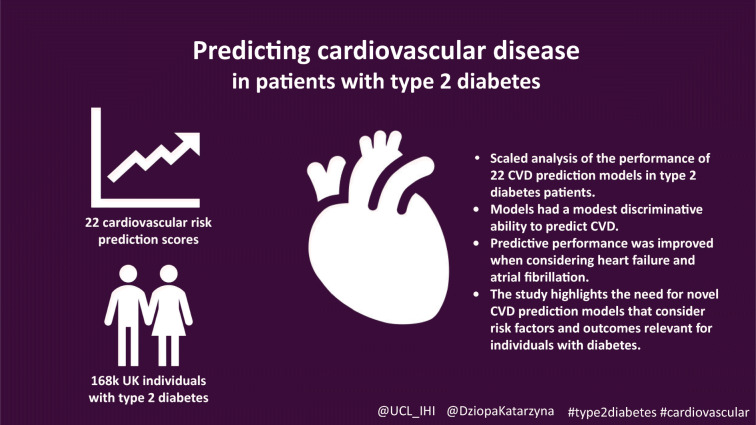

**Supplementary Information:**

The online version contains peer-reviewed but unedited supplementary material available at 10.1007/s00125-021-05640-y.

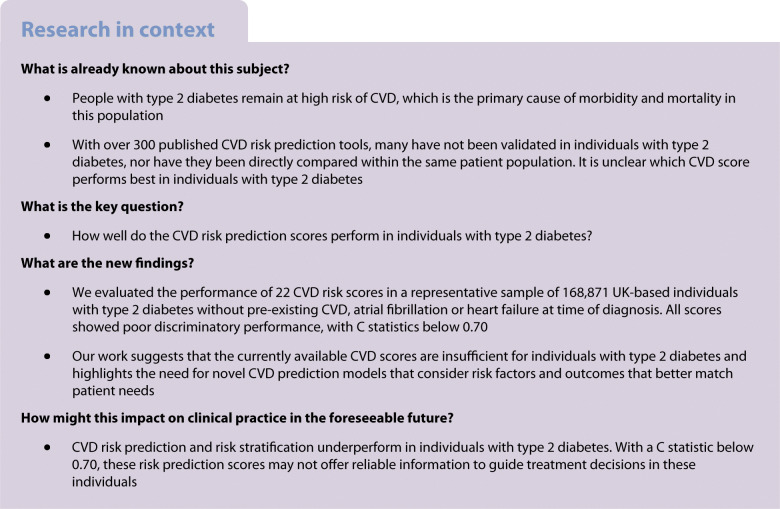



## Introduction

CVD treatment initiation and intensification in clinical practice are guided by risk prediction algorithms. The UK National Institute for Health and Care Excellence (NICE) guidelines pragmatically recommend the use of the QRISK2 risk prediction tool in people with and without diabetes. The American College of Cardiology/American Heart Association (ACC/AHA) recommends estimating the 10 year risk of CVD using the Atherosclerotic Cardiovascular Disease (ASCVD) risk score [1]. Contrary to this, the European Society of Cardiology (ESC) does not recommend a specific CVD risk prediction tool, and instead stratifies individuals into three categories based on risk factors including: presence of target organ damage, number of risk factors, diabetes duration and age [2].

Despite major advances in treatment, people with type 2 diabetes remain at high risk of CVD, the main cause of morbidity and mortality in this population [3]. There is, however, considerable heterogeneity in risk [4], supporting the need for risk-stratified management. With over 300 published CVD risk prediction tools [5], many of which have not been validated in individuals with type 2 diabetes, nor directly compared within the same patient population, it is unclear which CVD score performs best in people with diabetes. Previous comparisons only partially addressed this question, due to focusing on non-representative individuals with diabetes enrolled in drug trials [6], focusing on a relatively short follow-up [7] or using a modest sample of individuals [8], and with all focusing on a small subset of available scores, without exploring performance to predict CVD outcomes more relevant for those individuals. Quite apart from the greater CVD risk, even at a given level of individual risk factors, it is evident that the initial presentation of CVD in individuals with diabetes differs from that of the general population, with greater representation of heart failure (HF) and of peripheral artery disease (PAD), while haemorrhagic strokes are less frequent [9]. General population scores, and indeed many designed for people with diabetes, have focused largely on the prediction of CHD and stroke only.

Our aim was to inform the use of risk scores in clinical practice by quantifying the validity of existing risk scores in predicting standard CVD (CHD, stroke, PAD), as well as a broader definition of major CVD outcomes (CVD+) that includes HF and atrial fibrillation (AF), as these are more frequent outcomes in diabetic populations [10, 11]. Additionally, we explored the scores’ predictive performance against individual disease types: stroke, CHD, AF and HF. We also compared the performance of both bespoke CVD risk scores for individuals with diabetes and CVD risk scores for the general population. The latter are preferred for clinical practice as a single tool is simpler to deploy. While it is assumed that diabetes-specific scores may perform better in people with diabetes, no formal comparison with general population scores has been undertaken before. We first performed a literature review to identify CVD risk prediction scores, and subsequently validated these in a large UK-based electronic health records (EHR) dataset. We also performed key subgroup analyses, stratifying by sex, age, CVD history and treatment.

## Methods

### Literature review

A literature search for CVD risk assessment tools was performed using MEDLINE [12]. The search strategy focused on key words including ‘CVD’, ‘type 2 diabetes’, ‘risk assessment’ or ‘risk score’ and names of known risk scores. Please see the electronic supplementary material (ESM) Methods section and ESM Fig. 1 for more information.

### Cohort study of individuals with type 2 diabetes

A cohort of 168,871 individuals with type 2 diabetes (18 years or older without recorded CVD+ diagnosis prior or 30 days after the time of type 2 diabetes diagnosis) was extracted from Cardiovascular disease research using Linked Bespoke studies and Electronic health Records (CALIBER), linking three English EHR sources: primary care records from the Clinical Practice Research Datalink (CPRD), Hospital Episodes Statistics (HES) and national death registration from the Office for National Statistics (ONS) [13]; see ESM Methods. The study was approved by the Medicines and Healthcare products Regulatory Agency (MHRA) (UK) Independent Scientific Advisory Committee [17_155], under Section 251 (NHS Social Care Act 2006).

Individuals with type 2 diabetes in this dataset were identified based on a CALIBER phenotyping algorithm (https://www.caliberresearch.org/portal/phenotypes), harmonising and combining data from general practitioner (GP) records and HES; see ESM Table 1. The CALIBER phenotyping algorithms have been extensively validated, as described previously [14]. As primary care registration is close to universal in the UK and free at the point of delivery, with reimbursements based on correct entry of diagnostic codes, type 2 diabetes case ascertainment based on UK primary care data can be considered as highly accurate [15].

### Individual characteristics

The following participant characteristics and measurements were extracted: sex, age (years), smoking status, HbA_1c_, fasting plasma glucose (FPG), BMI, HDL- and LDL-cholesterol, total cholesterol, triacylglycerol, systolic blood pressure (SBP) and diastolic blood pressure (DBP), urine albumin/creatinine ratio, serum creatinine, C-reactive protein, total white blood cell count and ECG results (see ESM Methods and ESM Tables 2–4).

### Cardiovascular outcomes

Individuals were followed up from their initial type 2 diabetes diagnosis until their first cardiovascular event, death, end of study (5 February 2018) or 10 year follow-up landmark, whichever occurred first. Individuals with a previous record of AF were excluded due to the inability to differentiate between ongoing vs recurrent AF events in EHR. Individuals with any other pre-existing CVD event were excluded from the main analyses, and considered in subsequent subgroup analyses of performance in participants with pre-existing CVD at the time of diagnosis.

A CVD event was defined as the first occurrence of fatal or non-fatal myocardial infarction (MI), sudden cardiac death, ischaemic heart disease, fatal or non-fatal stroke, or PAD since diagnosis of type 2 diabetes. We additionally defined CVD+ as including HF and/or AF: ‘CVD + AF + HF’. Additionally, we explored performance against individual CVD components: CHD, stroke, AF and HF. Detailed CALIBER [13] endpoint definitions are provided in ESM Table 5.

### Statistical analysis

Models were evaluated on discrimination (using Harrell’s C statistic [16]) and calibration (calibration-in-the-large [CIL] and calibration slope [CS] [17]); see ESM Methods. We note that when predicting the occurrence of a binary outcome (such as a disease) at a single moment in time, the C statistic is identical to the area under a receiver operating characteristic (ROC) curve [16]. The C statistic varies from 1.0 (perfect discrimination) to 0.5 (random chance). It has been suggested that a C statistic below 0.70 indicates inadequate discrimination, between 0.70 and 0.80 acceptable discrimination, and between 0.80 and 0.90 excellent model discrimination [18]. Missing data were addressed using multiple imputation, and, for comparison’s sake, the results were compared with those obtained from a complete-case deletion dataset. Models were evaluated both before and after recalibration, a process whereby a model’s intercept and slope are updated to adapt a risk score to a different local setting, a similar but distinct outcome or both. Here, the risk scores were independently recalibrated to predict all six of the CVD endpoints described. To prevent model overfitting, recalibration was performed in a 10% (16,887) independent training sample, which is an ample sample size to estimate the two coefficients (the intercept and slope) necessary for model recalibration. The remaining 90% (151,984) of the dataset was used to compare like-with-like model performance of the uncalibrated and recalibrated models. Subgroup performance was explored for CVD history, sex, age and statin usage at the time of diagnosis. The discriminative ability of two models was formally compared by testing the difference in C statistics using the test data. The net reclassification index (NRI) for CVD was calculated using the test data (after model recalibration in the training data) to compare Systemic Coronary Risk Evaluation (SCORE) CVD against the following scores: QRISK2, QRISK3 and Cardiovascular Health Study (CHS) Basic.

Missing variables (presented in Table 1 and ESM Tables 2, 6, 7) were imputed using multiple imputation [19]. Imputation variables were selected using the procedure described previously [20], guarding against missing data while at the same time maximising predictive accuracy. Moreover, the procedure eliminates predictors whose proportion of usable cases fails to meet a minimum value (here 0.5). Imputation-specific results were combined using Rubin’s rules [21].

**Table 1:** Participant characteristics, with data sourced from around the time of type 2 diabetes diagnosis (1 year before to 1 week after diagnosis)

Clinical characteristic	Mean (SD) or *N* (%)	Median (Q1; Q3)	Missing data (%)
Total no. of individuals	168,871		
Follow-up time (years)		9.0 (5.3; 10.0)	
Women (%)	78,204 (46.3)		0.0
Age (years)	59.3 (13.9)	60.0 (50.0; 69.0)	0.0
HbA_1c_			55.2
mmol/mol	64.1 (20.6)	57.0 (49.0; 76.0)	
%	8.0 (4)	7.4 (6.6; 9.1)	
FPG (mmol/l)	9.7 (3.9)	8.1 (7.1; 11.0)	68.4
BMI (kg/m^2^)	31.9 (6.8)	30.9 (27.2; 35.5)	40.1
HDL-cholesterol (mmol/l)	1.2 (0.4)	1.2 (1.0; 1.4)	47.9
LDL-cholesterol (mmol/l)	3.2 (1.0)	3.1 (2.5; 3.9)	59.3
Total cholesterol (mmol/l)	5.4 (1.3)	5.3 (4.6; 6.2)	37.9
SBP (mmHg)	140 (18)	140 (130; 150)	25.8
Statin usage (before type 2 diabetes diagnosis)	43,102 (25.5)		
Smoking status^a^			23.3
Never smoked	67,828 (52.4)		
Ex-smoker	35,533 (27.4)		
Current smoker	26,165 (20.2)		
Townsend score			0.1
1 (least deprived)	32,058 (19.0)		
2	35,090 (20.8)		
3	35,255 (20.9)		
4	37,365 (22.1)		
5 (most deprived)	28,990 (17.2)		

Q1 and Q3 refer to lower and upper quartiles, accordingly

^a^The denominator for smoking status is 129,526 individuals, after excluding individuals with missing information

## Results

### Literature review

We included 15 publications reporting 22 different risk score models that predicted the 10 year risk of CVD. Only two of the scores were published before 2000 (Framingham 1991 [22], Framingham 1998 [23]) (ESM Results, ESM Tables 8, 9).

Out of the 22 identified CVD risk prediction models, nine were derived in individuals with type 2 diabetes alone (Risk Equations for Complications Of type 2 Diabetes [RECODE] [24], Diabetes Audit and Research in Tayside [DARTS] [25], UK Prospective Diabetes Study [UKPDS] 56 [26], UKPDS 68 Congestive Heart Failure [C-HF] and Stroke [27], UKPDS 82 C-HF and CHD [28], and CHS Basic and Advanced [29]), and 13 scores enrolled both non-diabetic individuals and individuals with type 2 diabetes (SCORE CHD and CVD [30]; Finrisk Stroke, CHD and CVD [31]; Framingham 1991 CHD, CVD and Stroke [22]; Framingham 1998 [23]; QRISK2 [32]; QRISK3 [33]; ASCVD [1]; and Reynolds Risk [34, 35]), and these were considered general population samples. Ten rules were designed to predict CVD, seven predicted CHD, three predicted stroke and two HF (ESM Table 8 [Type of predicted CVD reported]).

All the risk scores incorporated classic CVD risk factors, such as age, sex, blood pressure and smoking status. Twenty risk scores included information about lipids. The scores that included a proportion of individuals with diabetes typically included type 2 diabetes (presence/absence) as a predictor, but did not include diabetes-specific risk factors such as diabetes duration and glycaemic status (which are often used in diabetes-specific scores). The total number of predictors in these risk prediction models ranged from six (SCORE [30]) to 19 (QRISK3 [33]) (ESM Fig. 2, ESM Table 10).

### Individual characteristics and 10 year CVD outcomes

The baseline characteristics of the individuals are presented in Table 1 and ESM Tables 2, 6, 7. The mean age was 59.3 years (SD: 13.9), 78,204 (46%) participants were women and 43,102 (26%) individuals were on statins.

**Fig. 1:**
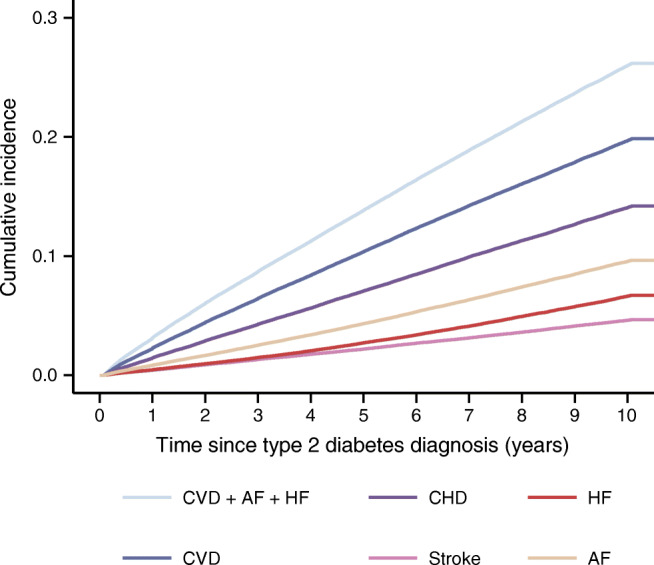
Kaplan–Meier estimates of the 10 year cumulative incidence of CVD after a diagnosis of type 2 diabetes.

During a median follow-up time of 10 years since type 2 diabetes diagnosis, 38,335 (22.70%) individuals suffered a CVD, AF or HF event. Of these, 29,025 (17.19%) had a CVD event, 20,628 (12.22%) CHD, 13,826 (8.19%) AF, 9465 (5.6%) HF and 6727 (3.98%) stroke (see Kaplain-Meier estimates in Fig. 1, ESM Table 11).

### Predicting cardiovascular risk in individuals with type 2 diabetes

**Fig. 2:**
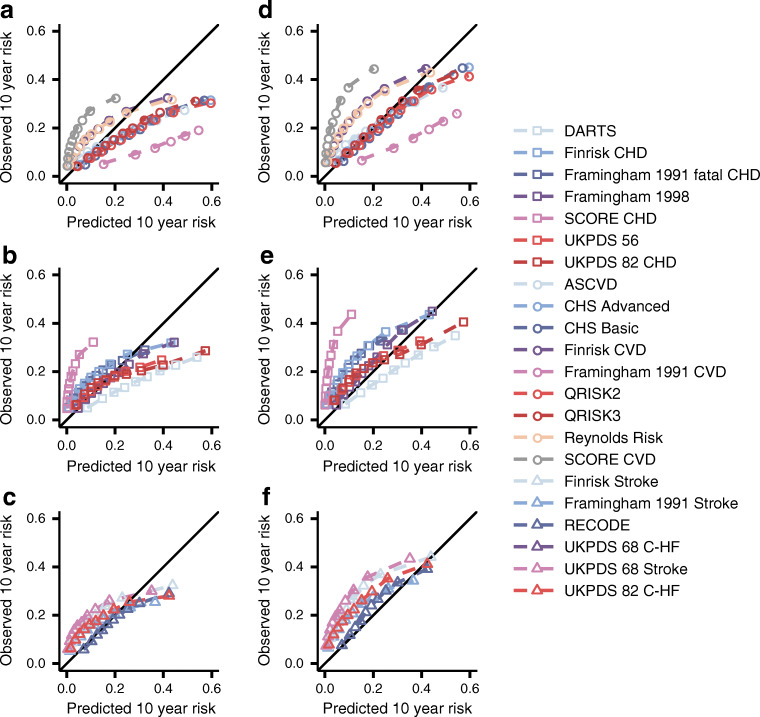
Calibration plots of 22 prediction rules for 10 year CVD risk, evaluated in individuals with type 2 diabetes. Estimates are based on imputed data. Depicted performance is based on 90% of the data used for external validation. The observed 10 year risk (*y* axes) is plotted against the mean predicted 10 year risk (*x* axes) within groups defined by quintiles of predicted risk. (**a**–**c**) The scores were evaluated against CVD; (**d**–**f**) scores were evaluated against CVD+. Scores were grouped by the type of outcome they originally attempted to predict: CVD (subplots **a**, **d**), CHD (subplots **b**, **e**) or other (including stroke, C-HF; subplots **c**, **f**). The diagonal line reflects perfect calibration

Results obtained from the complete case-analyses (see ESM Results) were similar to results from the multiple-imputation analysis. Nevertheless, because the complete-case analysis slightly overestimated model performance (ESM Figs. 3, 4, ESM Tables 12, 13), we present the later, more conservative, results in the main text (Fig. 2, ESM Figs. 5, 6, ESM Tables 14, 15).

Most models achieved similar calibration in CVD prediction (CS: from 0.38 to 0.74; CIL: from −1.89 to 2.26) (Fig. 2, ESM Table 14). Models designed to predict stroke and/or HF did not substantially underperform compared with CVD-derived models. The scores almost uniformly underestimated the risk of CVD+ (CS: from 0.41 to 0.88, CIL: from −1.50 to 2.69) (ESM Table 14), the exceptions being the Framingham 1991 CVD and DARTS scores which systematically overestimated risk.

**Fig. 3:**
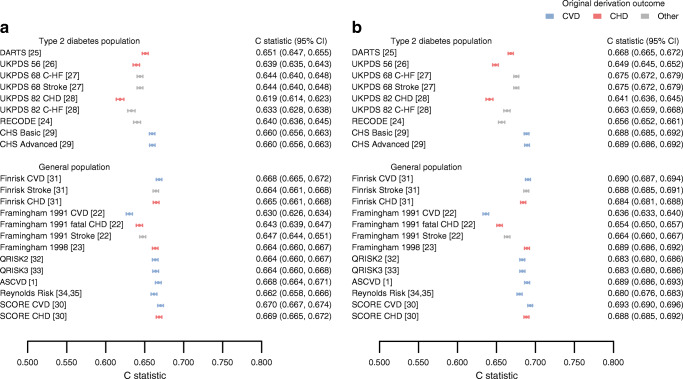
C statistics (discrimination) of 22 CVD risk prediction tools externally validated in a UK-based type 2 diabetes sample. Scores were split by the derivation population and the reported type of CVD outcome. Point estimates are presented alongside 95% CI. Results were based on imputed data and based on a 90% ‘test’ set of the total data used for external validation. (**a**) Scores evaluated against CVD; (**b**) scores evaluated against CVD+

The CHD Basic (CS: 0.86; CIL: −0.22), ASCVD (CS: 0.46; CIL: −0.19), QRISK2 (CS: 0.69; CIL: −0.25) and QRISK3 (CS: 0.72; CIL: −0.05) models (originally derived to predict any CVD) generally showed near-perfect calibration for CVD+. Focusing on scores not originally intended to predict CVD, we found that the Framingham 1998 score (a CHD score) could accurately predict both CVD (CS: 0.74 [95% CI 0.72, 0.76]; CIL: −0.15 [95% CI -0.16, −0.13]) and CVD+ (CS: 0.88 [95% CI 0.86, 0.90]; CIL: 0.23 [95% CI 0.22, 0.25]). For the ‘other’ group (including stroke and HF-derived scores), we found that RECODE for CVD (CS: 0.73 [95% CI 0.70, 0.76]; CIL: −0.2 [95% CI -0.21, −0.19]) and for CVD+ (CS: 0.85 [95% CI 0.82, 0.87]; CIL: 0.17 [95% CI 0.16, 0.18]) calibrated well (Fig. 2). Despite observing reasonable external calibration, models had more difficulty discriminating between individuals who experienced an event within 10 years of follow-up and those who remained event free: the C statistic ranged from 0.62 to 0.67 (95% CI 0.67, 0.67) for SCORE CVD (Fig. 3). Similar patterns of discrimination were observed when predicting CVD+, with this combined endpoint showing a minimally improved C statistic (from 0.64 to 0.69) compared with CVD, and again with SCORE CVD having the largest C statistic (0.69 [95% CI 0.69, 0.70]). Testing for the pairwise difference in C statistics (ESM Fig. 7) indicated that SCORE CVD outperformed all other scores aside from the ASCVD, Finrisk CVD and SCORE CHD. SCORE CVD also performed better than the nine diabetes-specific scores (Fig. 3, ESM Fig. 7). A net reclassification comparison (applied after model recalibration, see below) showed that SCORE CVD performed slightly better than QRISK2 and QRISK3 by assigning a lower risk to individuals who did not experience CVD in the available 10 years of follow-up (Table 2, ESM Table 16).

**Table 2:** A net reclassification table comparing the predicted CVD risk distributions of QRISK3 and SCORE CVD, among individuals with type 2 diabetes without and with a CVD event, during the available 10 years of follow-up

QRISK3	SCORE CVD			Total
	Low risk [0.0, 0.1)	Intermediate risk [0.1, 0.2)	High risk [0.2, 1.0]	
In participants without CVD				
Low risk	19,704	2618	2	22,324
Intermediate risk	9446	46,007	10,735	66,188
High risk	367	5721	31,178	37,266
Total	29,517	54,346	41,915	125,778
In participants with CVD				
Low risk	994	190	0	1184
Intermediate risk	903	7461	2732	11,096
High risk	48	1355	12,301	13,704
Total	1945	9006	15,033	25,984
NRI estimates				
NRI: 0.041 (0.036, 0.048)		Pr(Up|Event): 0.113 (0.109, 0.116)		
Event NRI: 0.024 (0.019, 0.031)		Pr(Down|Event): 0.089 (0.084, 0.091)		
Non-event NRI: 0.017 (0.015, 0.020)		Pr(Down|Non-event): 0.124 (0.122, 0.126)		
		Pr(Up|Non-event): 0.106 (0.105, 0.108)		

Calculations are based on the test data after recalibrating in an independent training dataset. Square brackets are used to indicate the endpoint is included, and parentheses to signal endpoint exclusion. Various NRI estimates are provided, including the probabilities of an increased (Up) or decreased (Down) predicted risk conditional on event status

Pr, probability

We observed that scores with more than ten predictors did not necessarily outperform scores with fewer variables: QRISK3 (19 variables) for CVD+ had a C statistic of 0.68 (95% CI 0.68, 0.69), compared with a C statistic of 0.69 (95% CI 0.69, 0.70) for SCORE CVD (six variables) and a C statistic of 0.69 (95% CI 0.69, 0.69) for the Framingham 1998 score (seven variables). Similar results were obtained for the CVD-only outcome. The scores derived from individuals with diabetes did not outperform scores derived in a population of non-diabetic individuals (Fig. 3).

### Predicting individual CVD endpoints and model recalibration

We additionally evaluated the ability of these 22 rules to predict individual CVD components: CHD, stroke, AF and HF. Here, we observed that, similar to CVD, predictions for CHD were slightly overestimated (ESM Figs. 3, 5). This overestimating was more severe when using these models to predict stroke, AF and HF (ESM Figs. 4, 6). Nevertheless, when considering AF and HF, the discriminative ability of these models slightly improved (C statistic ≥0.70) relative to CHD and CVD (ESM Figs. 8–11).

**Fig. 4:**
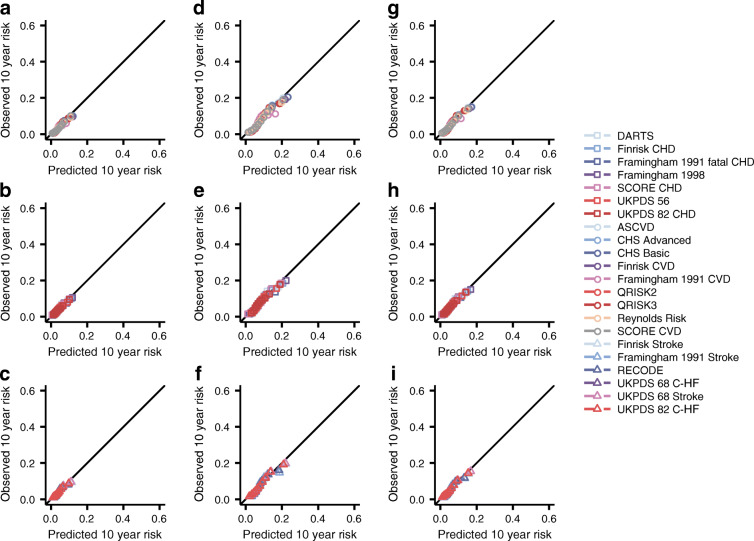
Calibration plots after recalibrating 22 prediction rules for 10 year CVD risk, evaluated in individuals with type 2 diabetes. Estimates are based on imputed data. Depicted performance is based on 90% of the data used for external validation, independent of the 10% hold-out sample used to recalibrate the models. The observed 10 year risk (*y* axes) is plotted against the mean predicted 10 year risk (*x* axes) within groups defined by quintiles of predicted risk. The scores were evaluated against (**a**–**c**) stroke; (**d**–**f**) AF; (**g**–**i**) HF. Scores were grouped by the derivation outcome CVD (subplots **a**, **d**, **g**), CHD (subplots **b**, **e**, **h**), or other (including stroke, C-HF; subplots **c**, **f**, **i**). The diagonal line reflects perfect calibration

**Fig. 5:**
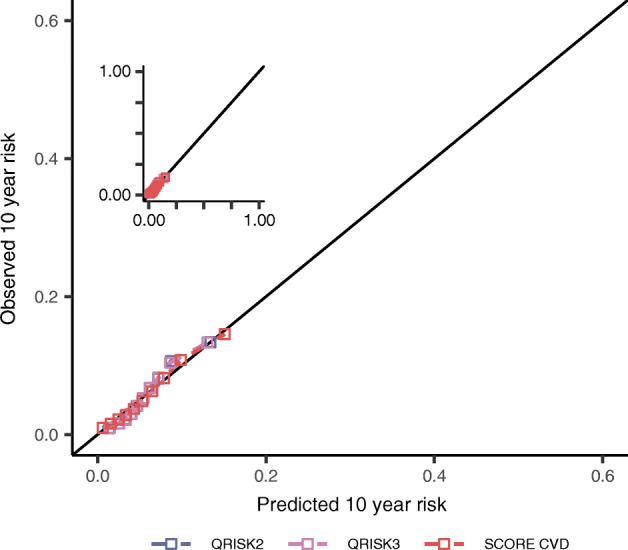
Calibration plots after recalibrating three prediction rules for the 10 year HF risk, evaluated in individuals with type 2 diabetes. Estimates are based on imputed data. Depicted performance is based on 90% of the data used for external validation, independent of the 10% hold-out sample used to recalibrate the models. The observed 10 year risk (*y* axes) is plotted against the mean predicted 10 year risk (*x* axes) within groups defined by quintiles of predicted risk

Recalibrating the 22 models using the 10% training dataset considerably improved calibration (CS: from 0.96 to 1.04) (ESM Figs. 12–14, ESM Tables 13, 15), with most rules showing near-perfect calibration in the test data. Given that most of these 22 rules were not designed to predict stroke, AF or HF, it was somewhat surprising to see that recalibration markedly improved performance for these endpoints as well, (Fig. 4). For example, after recalibration, QRISK3 could predict HF risk (CS: 1.09 [95% CI 1.02, 1.17]) and AF risk (CS: 1.08 [95% CI 1.00, 1.16]) remarkably well (Fig. 5).

### Subgroup analyses

**Fig. 6:**
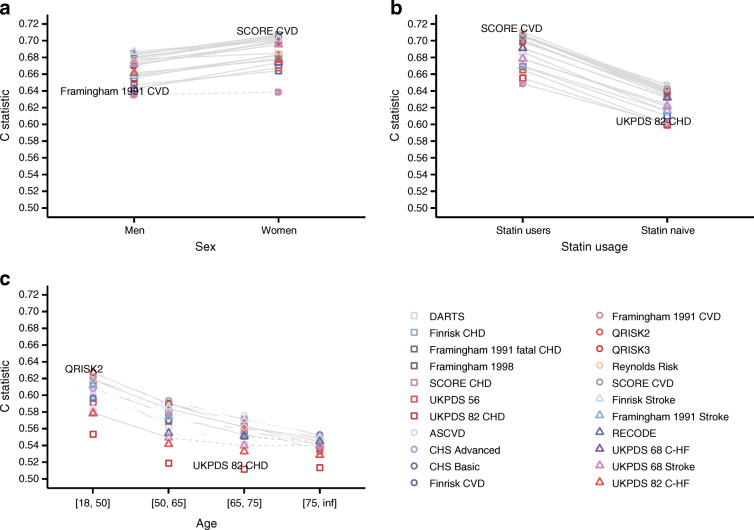
Subgroup-specific discrimination of 22 scores predicting CVD in individuals with type 2 diabetes without pre-existing CVD at the time of diagnosis. Results were based on imputed data (individuals with type 2 diabetes without pre-existing CVD) and based on 90% of the data used for external validation. Point estimates and 95% CI are presented in ESM Tables 17–20. Plots are stratified by (**a**) sex, (**b**) statin use and (**c**) age. Dashed lines indicate non-significant (at an α of 0.05) difference between subgroup-specific C statistics, with solid lines indicating significant differences. Inf, positive infinity

Next, in individuals with type 2 diabetes without CVD+ at baseline, we explored the discriminative ability of these CVD scores in subgroup analyses of age, sex and statin usage (Fig. 6, ESM Fig. 15). Subgroup changes in performance were shared across the various scores, where discriminative ability was lower for men and statin naive and older individuals (significance interaction tests indicated by a solid line in Fig. 6, ESM Tables 17–19).

**Fig. 7:**
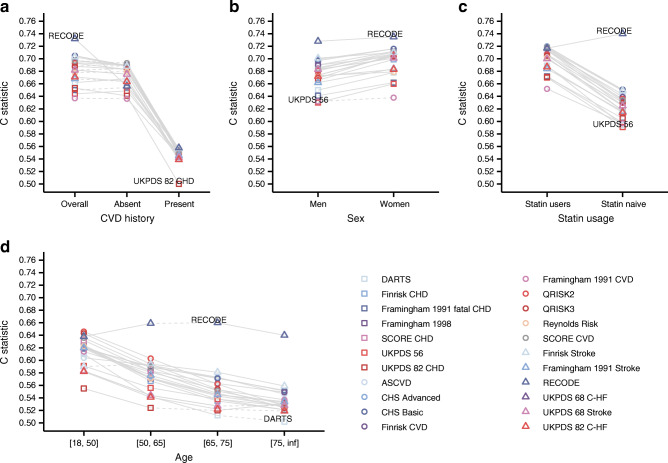
Subgroup-specific discrimination of 22 scores predicting CVD in individuals with type 2 diabetes irrespective of their history of CVD at the time of type 2 diabetes diagnosis. Results were based on imputed data (all individuals with type 2 diabetes irrespective of their baseline CVD status) and based on 90% of the data used for external validation. Point estimates and 95% CI are presented in ESM Tables 17–20. Plots are stratified by (**a**) CVD history at baseline, (**b**) sex, (**c**) statin use and (**d**) age. Dashed lines indicate non-significant (at an α of 0.05) difference between subgroup-specific C statistics, with solid lines indicating significant differences. Inf, positive infinity

We additionally performed similar subgroup analyses for individuals with type 2 diabetes irrespective of their baseline CVD+ status (see Clinical characteristics in ESM Table 20), finding similar patterns of discrimination as for individuals without CVD+ at baseline (Fig. 7, ESM Fig. 16, ESM Tables 21–24). The results showed that score performance was significantly worse for people with pre-existing CVD+ at the time of type 2 diabetes diagnosis. Finally, we observed that RECODE performed best (a C statistic of 0.73 [95% CI 0.73, 0.74] for CVD+) in a sample of people with type 2 diabetes including individuals with and without CVD+ history at the time of diagnosis.

## Discussion

We validated 22 cardiovascular risk scores for their ability to predict a range of macrovascular endpoints in a cohort of 168,871 people with type 2 diabetes. We report several unique findings. First, with discriminative abilities below 0.70 (C statistic), the scores performed universally poorly, which was compounded in individuals with diabetes and established CVD at baseline (C statistics close to 0.50). Second, diabetes-specific scores did not appear to be superior to scores derived for the general population, and in fact were outperformed by, for example, the general population SCORE CVD rule. Third, scores with many additional features did not outperform those with fewer and more readily available (in primary care) predictors. Finally, a simple recalibration step markedly improved score performance, repurposing scores intended to predict any CVD or CHD to accurately predict stroke, AF and HF risk (see Fig. 4).

We externally evaluated two risk prediction scores widely used in the UK (QRISK2 and QRISK3), which had good discriminatory ability in the general population (C statistics for QRISK2 of 0.82 in women and 0.79 in men, and for QRISK3 0.88 in women and 0.86 in men), and found considerable attenuations in their discriminative ability when applied in individuals with type 2 diabetes (e.g., C statistic 0.66 [95% CI 0.66, 0.67] for QRISK2). This poor performance may be somewhat surprising given that the QRISK scores were derived in a similar, but independent, sample of English individuals. While the difference in discrimination between SCORE CVD (0.69 [95% CI 0.69, 0.70]), QRISK2 (0.68 [95% CI 0.68, 0.69]) and QRISK3 (0.68 [95% CI 0.68, 0.69]) was statistically significant (interaction p value <0.001 for CVD and CVD+), we note that the magnitude of the difference in the C statistic was small and is unlikely to have clinical implications. This may be better appreciated when considering the net reclassification analysis: comparing SCORE CVD with QRISK2 (non-event NRI 0.009, ESM Table 16) and with QRISK3 (non-event NRI 0.017, Table 2) resulted in very modest improvements.

The predictive performance of the risk scores was markedly poorer in individuals with pre-existing CVD at the time of type 2 diabetes diagnosis (C statistic ranged from 0.50 to 0.54 for the CVD outcome). Most of the prediction models were developed in individuals without clinical manifestations of CVD and were not validated in people with established CVD. Moreover, these risk scores lack predictors that are of particular importance to individuals with established disease, such as time since the first diagnosis of CVD, history of CVD and renal function [36]. The improved performance of the RECODE score (C statistic >0.70), when considering all participants with type 2 diabetes irrespective of their history of CVD, is likely related to its development in participants with diabetes with similar mixed histories of CVD. Here, we show that the inclusion of people with a mixed history of disease at the time of diabetes diagnosis, combined with appropriate modelling choices, may improve (instead of worsening) score performance.

The necessity for a diabetes-specific CVD score has often been discussed [7] and revolves around the need to account for excess risk unexplained by conventional risk factors, and the desire to include diabetes-specific variables such as HbA_1c_ and diabetes duration, which are known observationally to predict CVD risk [37]. It has been suggested that a diabetes-specific score can better deal with exposure and outcome associations specific to individuals with type 2 diabetes [25]. Despite these arguments, we did not observe a general benefit of diabetes-specific rules (including diabetes-specific variables) compared with scores derived in samples with a mixture of individuals with type 2 diabetes and the general population. This suggests that the presence of other risk factors which may often correlate among themselves, such as diabetes duration and HbA_1c_, does not markedly contribute to model performance. Similarly, we note that despite comparing risk prediction tools derived over the course of more than three decades, during which health and healthcare have generally improved, the almost uniform performance of these scores in the present contemporary sample of individuals with type 2 diabetes illustrates that these healthcare changes have not affected the external performance of these models.

Due to the inherent limitations of EHR data, some predictor variables were infrequently measured (ESM Table 3), which we attempted to address through multiple imputation. Possibly this reliance on imputed data biased results; however, we did not observe a meaningful difference in performance between complex models such as QRISK3 (requiring 19 variables) and more straightforward models such as SCORE CVD and CHD (requiring only six variables). Furthermore, while medical history and prescription data could be readily extracted from prior to the time of type 2 diabetes diagnosis, measured risk factors, such as blood pressure, were extracted using a window of 12 months before and 1 week after diagnosis. While this does reflect data availability in a real-world setting, medical professionals intending to use the risk prediction tools will likely actively measure key variables, especially if these are readily obtained, such as BMI and blood pressure. Thus, we may have underestimated the true performance of these risk prediction scores in a more ideal setting.

Performing analyses in primary care records, where such risk scores are deployed in practice, provides an appropriate platform for validation studies. We acknowledge that such data involve issues with missing data and coding errors, but this better reflects their ‘true’ value in contrast to the more artificial situation of a research cohort study or clinical trial. Furthermore, the use of real-life clinical data takes a ‘whole population’ approach, whereas cohorts and trials apply restrictions to entry.

The calibration (agreement between observed and predicted risk) was generally reasonable for all scores and could readily be improved by recalibrating the models in an independent training set. This recalibration was also successful in repurposing models to predict endpoints outside their intended use. Here, we reiterate that all performance metrics, discrimination (C statistic) and calibration, were estimated in an independent test dataset fairly assessing performance without the over-optimism observed when calculating these metrics in the same training data used to recalibrate the scores (or when deriving a model de novo). The near-optimal calibration additionally highlights that the recalibrated models were not overfitted and utilised a sufficiently large training sample size, which would otherwise result in over- or under-estimating the true risk in an independent test dataset. This is perhaps most clearly shown by comparing the calibration plots presented by van der Leeuw and colleagues [8] derived in a small sample of 584 individuals with type 2 diabetes with the calibration observed in the current analysis using more than 16,000 individuals with diabetes (Figs 2, 4, 5). Previous studies have typically focused on any CVD outcome, or individual endpoints such as CHD and any stroke. Here, we show that such models can be used to identify individuals with type 2 diabetes at increased risk for the composite endpoint CVD + HF + AF (since AF and HF occur much more frequently in these individuals [38]). While we showed reasonable out-of-the-box calibration, recalibration improved performance to near-perfect agreement and we propose that recalibration is more frequently considered before applying any model to local settings. Given the modest sample size (a few hundred cases) required to accurately recalibrate a model [39], combined with the increased availability of EHR data, such recalibration could be readily applied by healthcare commissioners at a local level. The adjustment of prediction models to local settings, so-called model updating (recalibration), typically requires a fraction of the time and data (often 100–200 cases should be sufficient [39]), and provides an attractive and efficient alternative to the derivation of a completely new model for each local setting, particularly if one also considers the need for independent replication data to fairly assess model performance. Furthermore, reclassification analyses similar to the ones presented here presuppose that the models are reasonably calibrated. To facilitate model recalibration to local clinical settings, we have appended a straightforward computer application (https://gitlab.com/cvd_in_t2dm/recalibration), which we are committed to support and adjust pending local requirements.

In summary, we have shown that CVD risk scores derived in the general population performed worse in people with type 2 diabetes. CVD risk scores derived in individuals with diabetes did not in general perform better, emphasising the difficulties of accurately predicting CVD in a relatively high-risk population. The performance of the scores was also similar for the wider outcome definition of CVD, which includes HF and AF which occur more frequently in individuals with diabetes.

## Supplementary Information

ESM 1 (PDF 3.67 mb)

## Data Availability

The data that support the findings of this study are available from the Clinical Practice Research Datalink (https://www.cprd.com/), but restrictions apply to the availability of these data, which were used under license for the current study, and so they are not publicly available. HDR UK CALIBER Phenotype Library: https://portal.caliberresearch.org/.

## Abbreviations

AF: Atrial fibrillation
ASCVD: Atherosclerotic Cardiovascular Disease
CALIBER: Cardiovascular disease research using Linked Bespoke studies and Electronic health Records
C-HF: Congestive Heart Failure
CHS: Cardiovascular Health Study
CIL: Calibration-in-the-large
CS: Calibration slope
CVD+: CVD including heart failure and/or atrial fibrillation.
DARTS: Diabetes Audit and Research in Tayside
EHR: Electronic health records
FPG: Fasting plasma glucose
HES: Hospital Episodes Statistics
HF: Heart failure
NRI: Net reclassification index
PAD: Peripheral artery disease
RECODE: Risk Equations for Complications Of type 2 Diabetes
SBP: Systolic blood pressure
SCORE: Systemic Coronary Risk Evaluation
UKPDS: UK Prospective Diabetes Study
