# Supplementary material for: Cardiovascular risk prediction in type 2 diabetes: a comparison of 22 risk scores in primary care settings
Source: Diabetologia. 2022 Jan 15;65(4):644–56. doi: 10.1007/s00125-021-05640-y (PMC8894164; doi:10.1007/s00125-021-05640-y)
Supplement: Supplementary file 1 — (PDF 3.67 mb) [file 125_2021_5640_MOESM1_ESM.pdf]

Electronic supplementary material (ESM)

Abbreviations used in the ESM

**Abbreviation**	**Full text**
AF	Atrial Fibrillation
ASCVD	Atherosclerotic Cardiovascular Disease
CALIBER	Cardiovascular disease research using Linked Bespoke studies and Electronic health Records
C-HF	Congestive Heart Failure
CHD	Coronary Heart Disease
CHS	Cardiovascular Health Study
CIL	Calibration-in-the-large
Cox's PH model	Cox Proportional-Hazards Model
CS	Calibration Slope
CVD	Cardiovascular disease
CVD+ or CVD+AF+HF	Cardiovascular disease including heart failure (HF) and/or atrial fibrillation (AF)
DARTS	Diabetes Audit and Research in Tayside
DBP	Diastolic Blood Pressure
ECG	Electrocardiography
FPG	Fasting plasma glucose
HbA_{1c}	Glycated haemoglobin
HES	Hospital Episodes Statistics
HF	Heart Failure
HIV	Human Immunodeficiency Virus
MI	Myocardial Infarction
SBP	Systolic Blood Pressure
T2DM	Type 2 diabetes
Weibull AFT model	Weibull Accelerated Failure Time model

ESM Methods

Literature review

A comprehensive literature search for CVD risk assessment tools was performed using MEDLINE. The search strategy focused on key words including "CVD", "type 2 diabetes", "risk assessment" or "risk score" and names of known risk scores. In addition to the previous search strategy [1], we used the following MEDLINE search query:

(diabetes mellitus, **type** 2[Majr:NoExp] **OR** diabetes mellitus **type** 2[Title/Abstract] **OR** Diabetic Angiopathies[Majr:NoExp] **OR** diabetic angiopath*[Title/**Abstract**])

AND

(risk assessment[Majr:NoExp] **OR** risk assessment*[Title/**Abstract**] **OR** predictive value **of** tests[Majr] **OR** "predictive value of test*"[Title/**Abstract**] **OR** sensitivity **and** specificity[Majr] **OR** reproducibility **of** results[MeSH Terms] **OR** risk score*[Title/**Abstract**] **OR** risk calculat*[Title/**Abstract**] **OR** risk engine* **OR** risk equation*[Title/**Abstract**] **OR** risk algorithm*[Title/**Abstract**] **OR** risk table*[Title/**Abstract**] **OR** risk predict*[Title/**Abstract**] **OR** risk **function***[Title/**Abstract**] **OR** framingham risk*[Title/**Abstract**] **OR** framingham equation*[Title/**Abstract**] **OR** framingham model*[Title/**Abstract**] **OR** PROCAM[Title/**Abstract**] **OR** decode[Title/**Abstract**] **OR** ukpds[Title/**Abstract**] **OR** copenhagen risk*[Title/**Abstract**] **OR** British Regional Heart Study[Title/**Abstract**] **OR** Swedish National Diabetes Register[Title/**Abstract**] **OR** Tayside[Title/**Abstract**] **OR** qrisk[Title/**Abstract**] **OR** Reynolds risk[Title/**Abstract**] **OR** Dundee risk[Title/**Abstract**] **OR** brhs score*[Title/**Abstract**] **OR** British Family Heart Study[Title/**Abstract**] **OR** **New** Zealand chart[Title/**Abstract**] **OR** seven countries study[Title/**Abstract**] **OR** Progetto CUORE[Title/**Abstract**]) **AND** (Cardiovascular Diseases[MeSH Terms] **OR** cardiovascular disease*[Title/**Abstract**] **OR** cardiovascular disorder*[Title/**Abstract**] **OR** Coronary Artery Disease[MeSH Terms] **OR** coronary artery disease[Title/**Abstract**] **OR** coronary artery disorder*[Title/**Abstract**] **OR** Stroke[MeSH Terms] **OR** stroke[Title/**Abstract**])

In order to limit the number of irrelevant articles, we altered most of the Mesh headings to only search for major entries, excluding subheadings. The search query was limited to articles published between 2008-06-30 and 2019-01-16. Reference lists of papers and validation publications such as [1] [2] [3] were screened to identify additional publications reporting risk prediction models. Two reviewers (K.Dziopa, J. Gratton) independently reviewed the identified titles and abstracts, followed by full-text papers. Publications before 2008-06-30 were searched for using a previous review [1].

Risk prediction models were included if they: (1) were derived from prospective cohort studies or randomized trials; (2) were derived in a general (with or without the exclusion of people with diabetes) or in a diabetic population; (3) reported a measure of performance and assessed the 10-year risk of CVD, stroke, CHD, AF, HF or any combination, and (4) contained sufficient information to be applied in the validation dataset. The following information was extracted from the publications: the derivation population, the statistical model (e.g., Cox, logistic regression, Weibull), year of publication, type of CVD, follow-up time and predictor definitions. For presentation purposes, rules were grouped based on their derivation outcome: CVD, CHD, or other (including stroke and HF).

Cohort study of patients with type 2 diabetes

The CALIBER platform contains 'research ready' variables extracted from linked National Health Service (NHS) electronic health records and administrative health data. Data sources accessible through this platform include primary care, secondary care, national registry of social deprivation and mortality data from the Office of National Statistics [4]. In 2018, 10.6 million patients from 411 English general practices were included; records for 9.1 million (86%) patients were considered research quality, of which 8 million (88%) had a valid NHS number and were eligible for linkage in the Clinical Practice Research Datalink (CPRD) [5]. While this linked set of patient records has national coverage, subjects can be lost to follow-up when they leave the country or opt-out from sharing their health records, and those patients are excluded from the database [5].

The type 2 diabetes and CVD phenotype definitions utilized in the current study have previously been validated using cross-EHR, prognostic data, as well as through replication of aetiology associations [6].

Patient characteristics

The following patient characteristics and measurements were extracted (see ESM Table 3): sex, age (years), smoking status, glycated hemoglobin (HbA_{1c}), fasting plasma glucose (FPG), body mass index (BMI), HDL and LDL cholesterol, total cholesterol, triglycerides, systolic blood pressure (SBP) and diastolic blood pressure (DBP), urine albumin to creatinine ratio, serum creatinine, C-reactive protein, total white blood cell count, and electrocardiogram (ECG) results. Baseline predictor values were defined as measurements recorded closest to baseline (type 2 diabetes diagnosis date) and no more than 1 year prior or 1 week after the date of diagnosis of diabetes. The impact of a more liberal time window is described in ESM Table 3; and the cut off values applied for the continuous variables are described in ESM Table 4. Predictors without a measurement within this time frame were defined as missing. Information was also collected on the presence or absence of rheumatoid arthritis, renal disease, foot amputation, systemic lupus erythematosus, Human Immunodeficiency Virus (HIV) infection, mental disorders, microalbuminuria, erectile dysfunction, hypertension, and migraine at baseline. Prescriptions of the following drugs were extracted: anticoagulants, diuretics, corticosteroids, statins, and blood pressure lowering medication. Finally, information on social deprivation (Townsend score) and family history of CVD, CHD, MI and stroke were sourced.

Statistical Analysis

The calibration process describes the agreement between the predicted 10-year risk and the observed 10-year risk and consists of two complementary measures: calibration-in-the-large and the calibration slope. Calibration-in-the-large compares the mean predicted risk and mean observed risks, the calibration slope represent the amount a model over or underpredicts the risk in either the low- or high-risk groups. Perfect calibration is achieved when calibration-in-the-large is value 0 and the calibration slope is 1.

A model's discriminative performance represents the model's ability to distinguish between patients who will develop an event and patients who will not get an event during the considered follow-up time. Here we estimated Harrell's c-statistic as a measure of discrimination, where ac-statistic value of 1 denotes perfect discrimination, and a value of 0.5 denotes random discrimination.

Analyses were carried out in R version 4.0.3, calibration plots were generated using the *ggplot2* package [7], statistical analyses were performed using *Hmisc*[8], and forest plots were generated using the *metafor* package [9].

ESM Results

Literature review

The literature review retrieved 1,171 potentially relevant articles, of which 42 were retained after title and abstract screening. The majority were excluded due to poor or no validation, lack of performance metrics, unspecified or too short follow-up time, or other missing information necessary to implement the models. After screening the full texts, we excluded 14 publications due to

short follow-up time (less than 10 years), 4 due to the absence of predictors in the CALIBER database, 2 due to the lack of information provided for model implementation, 2 without internal validation results, 3 with point risk scores, 1 which was not published in English, and 1 which did not present a new risk score (ESM Fig. 1).

Predicting cardiovascular risk in patients with type 2 diabetes.

In the complete-case CALIBER dataset, most risk scores had moderate discrimination. The c-statistic ranged from 0.55 (UKPDS 68) to 0.67 (QRISK2) for the CVD outcome; from 0.55 (UKPDS 68) to 0.65 (QRISK2) for the CHD outcome; from 0.58 (UKPDS 68) to 0.69 (ASCVD) for the CVD, AF and HF outcomes; from 0.54 (UKPDS 68) to 0.70 (Framingham 1998, ASCVD, SCORE CVD, Finrisk CVD) for the Stroke outcome; from 0.59 to 0.73 (CHS Advanced) for the AF outcome; from 0.62 (UKPDS 68) to 0.72 (Framingham 1998) for the HF outcome.

ESM References

1. Chamnan P, Simmons RK, Sharp SJ, Griffin SJ, Wareham NJ (2009) Cardiovascular risk assessment scores for people with diabetes: a systematic review. Diabetologia 52(10):2001–2014. https://doi.org/10.1007/s00125-009-1454-0

2. Read SH, van Diepen M, Colhoun HM, et al (2018) Performance of Cardiovascular Disease Risk Scores in People Diagnosed With Type 2 Diabetes: External Validation Using Data From the National Scottish Diabetes Register. Diabetes Care 41(9):2010–2018. https://doi.org/10.2337/dc18-0578

3. van der Leeuw J, van Dieren S, Beulens JWJ, et al (2015) The validation of cardiovascular risk scores for patients with type 2 diabetes mellitus. Heart Br Card Soc 101(3):222–229. https://doi.org/10.1136/heartjnl-2014-306068

4. CPRD linked data | CPRD. https://www.cprd.com/linked-data. Accessed 25 Apr 2021

5. Padmanabhan S, Carty L, Cameron E, Ghosh RE, Williams R, Strongman H (2019) Approach to record linkage of primary care data from Clinical Practice Research Datalink to other health-related patient data: overview and implications. Eur J Epidemiol 34(1):91–99. https://doi.org/10.1007/s10654-018-0442-4

6. Denaxas S, Gonzalez-Izquierdo A, Direk K, et al (2019) UK phenomics platform for developing and validating electronic health record phenotypes: CALIBER. J Am Med Inform Assoc 26(12):1545–1559. https://doi.org/10.1093/jamia/ocz105

7. Wickham H, Chang W, Henry L, et al (2019) ggplot2: Create Elegant Data Visualisations Using the Grammar of Graphics

8. Jr FEH, others with contributions from CD and many (2020) Hmisc: Harrell Miscellaneous

9. Viechtbauer W (2010) Conducting Meta-Analyses in R with the metafor Package. J Stat Softw 36(1):1–48. https://doi.org/10.18637/jss.v036.i03

10. Vartiainen E, Laatikainen T, Peltonen M, Puska P (2016) Predicting Coronary Heart Disease and Stroke: The FINRISK Calculator. Glob Heart 11(2):213–216. https://doi.org/10.1016/j.gheart.2016.04.007

11. Donnan PT, Donnelly L, New JP, Morris AD (2006) Derivation and validation of a prediction score for major coronary heart disease events in a U.K. type 2 diabetic population. Diabetes Care 29(6):1231–1236. https://doi.org/10.2337/dc05-1911

12. Anderson KM, Odell PM, Wilson PW, Kannel WB (1991) Cardiovascular disease risk profiles. Am Heart J 121(1 Pt 2):293–298. https://doi.org/10.1016/0002-8703(91)90861-b

13. Prediction of Coronary Heart Disease Using Risk Factor Categories | Circulation. https://www.ahajournals.org/doi/full/10.1161/01.cir.97.18.1837. Accessed 10 Mar 2020

14. Hippisley-Cox J, Coupland C, Vinogradova Y, et al (2008) Predicting cardiovascular risk in England and Wales: prospective derivation and validation of QRISK2. BMJ 336(7659):1475–1482. https://doi.org/10.1136/bmj.39609.449676.25

15. Development and validation of QRISK3 risk prediction algorithms to estimate future risk of cardiovascular disease: prospective cohort study | The BMJ. https://www.bmj.com/content/357/bmj.j2099. Accessed 10 Mar 2020

16. Estimation of ten-year risk of fatal cardiovascular disease in Europe: the SCORE project | European Heart Journal | Oxford Academic. https://academic.oup.com/eurheartj/article/24/11/987/427645. Accessed 10 Mar 2020

17. Stevens RJ, Kothari V, Adler AI, Stratton IM, United Kingdom Prospective Diabetes Study (UKPDS) Group (2001) The UKPDS risk engine: a model for the risk of coronary heart disease in Type II diabetes (UKPDS 56). Clin Sci Lond Engl 1979 101(6):671–679

18. Clarke PM, Gray AM, Briggs A, et al (2004) A model to estimate the lifetime health outcomes of patients with type 2 diabetes: the United Kingdom Prospective Diabetes Study (UKPDS) Outcomes Model (UKPDS no. 68). Diabetologia 47(10):1747–1759. https://doi.org/10.1007/s00125-004-1527-z

19. Hayes AJ, Leal J, Gray AM, Holman RR, Clarke PM (2013) UKPDS outcomes model 2: a new version of a model to simulate lifetime health outcomes of patients with type 2 diabetes mellitus using data from the 30 year United Kingdom Prospective Diabetes Study: UKPDS 82. Diabetologia 56(9):1925–1933. https://doi.org/10.1007/s00125-013-2940-y

20. Goff DC, Lloyd-Jones DM, Bennett G, et al (2014) 2013 ACC/AHA guideline on the assessment of cardiovascular risk: a report of the American College of Cardiology/American Heart Association Task Force on Practice Guidelines. Circulation 129(25 Suppl 2):S49-73. https://doi.org/10.1161/01.cir.0000437741.48606.98

21. Basu S, Sussman JB, Berkowitz SA, Hayward RA, Yudkin JS (2017) Development and validation of Risk Equations for Complications Of type 2 Diabetes (RECODe) using individual participant data from randomised trials. Lancet Diabetes Endocrinol 5(10):788–798. https://doi.org/10.1016/S2213-8587(17)30221-8

22. Ridker PM, Paynter NP, Rifai N, Gaziano JM, Cook NR (2008) C-Reactive Protein and Parental History Improve Global Cardiovascular Risk Prediction: The Reynolds Risk Score for Men. Circulation 118(22):2243–2251. https://doi.org/10.1161/CIRCULATIONAHA.108.814251

23. Ridker PM, Buring JE, Rifai N, Cook NR (2007) Development and validation of improved algorithms for the assessment of global cardiovascular risk in women: the Reynolds Risk Score. JAMA 297(6):611–619. https://doi.org/10.1001/jama.297.6.611

24. Mukamal KJ, Kizer JR, Djoussé L, et al (2013) Prediction and classification of cardiovascular disease risk in older adults with diabetes. Diabetologia 56(2):275–283. https://doi.org/10.1007/s00125-012-2772-1

ESM Tables

ESM Table 1 Type 2 diabetes definition based on the CALIBER dataset. This is a composite of GP diagnosis of diabetes, diabetes diagnoses from primary and secondary care.

Definition / Data Source	Link to CALIBER research platform
**Diabetes Phenotype definition**	https://www.caliberresearch.org/portal/show/phenotype_diabetes (extract patients with type 2 diabetes)
GP diagnosis of diabetes	https://www.caliberresearch.org/portal/show/diabdiag_gprd
Diabetes diagnosis (primary care)	https://www.caliberresearch.org/portal/show/dm_gprd
Diabetes diagnosis (secondary care)	https://www.caliberresearch.org/portal/show/dm_hes<br>ICD-10 codes: E10-13, O240-243, G590, G632, H280, H360, M142, N083

ESM Table 2 Clinical characteristics of the continuous variables around the time (1 year before to 1 week after) of type 2 diabetes diagnosis.

Clinical characteristics	Mean (SD) or N (%)	Median (Q1; Q3)	Missing data (% of total no. of individuals)
Total no. of individuals	168,871		
Total cholesterol (mmol / L)	5.4 (1.3)	5.3 (4.6; 6.2)	37.9
Triglycerides (serum and plasma) (mmol / L)	2.4 (2.3)	1.9 (1.4; 2.8)	48.2
DBP (mmHg)	82 (10)	80 (76; 89)	25.8
Urine albumin to creatinine ratio	3.5 (10.6)	1.1 (0.6; 2.3)	90.0
Serum creatinine (micromol / L)	83.5 (23.4)	81.0 (70.0; 94.0)	33.5
C reactive protein (mg / L)	12.3 (25.3)	6.0 (3.0; 11.6)	91.7
Total white blood cell count (10^9 / L)	7.6 (2.3)	7.3 (6.1; 8.8)	52.7

ESM Table 3 Comparison of the clinical characteristics of the continuous variables around the time of type 2 diabetes diagnosis, in patients without pre-existing CVD at diagnosis.

	**Timeframe: 1 year before and 1 week after the diagnosis of type 2 diabetes**			**Timeframe: 1 year before and 3 months after the diagnosis of type 2 diabetes**		
**Clinical characteristics**	**Mean (SD) or N (%)**	**Median (Q1; Q3)**	**Missing data** (% of total no. of individuals)	**Mean (SD) or N (%)**	**Median (Q1; Q3)**	**Missing data** (% of total no. of individuals)
Total no. of individuals	168,871			168,871		
Follow-up time (years)		9.0 (5.3; 10.0)			9.0 (5.3; 10.0)	
Women (%)	78,204 (46.3)		0.0	78,204 (46.3)		0.0
Age (years)	59.3 (13.9)	60.0 (50.0; 69.0)	0.0	59.3 (13.9)	60.0 (50.0; 69.0)	0.0
HbA_{1c} (mmol/mol)<br>HbA_{1c} (%)	64.1 (20.6)<br>8.0 (4)	57.0 (49.0; 76.0)<br>7.4 (6.6; 9.1)	55.2	60.5 (18.6)<br>7.7 (3.9)	55.0 (47.5; 69.4)<br>7.2 (6.5; 8.5)	39.2
FPG (mmol / L)	9.7 (3.9)	8.1 (7.1; 11.0)	68.4	9.4 (3.8)	8.0 (7.0; 10.5)	67.0
BMI (kg / m^2)	31.9 (6.8)	30.9 (27.2; 35.5)	40.1	31.5 (6.6)	30.5 (26.9; 35.0)	29.1
HDL cholesterol (mmol / L)	1.2 (0.4)	1.2 (1.0; 1.4)	47.9	1.2 (0.4)	1.2 (1.0; 1.4)	41.5
LDL cholesterol (mmol / L)	3.2 (1.0)	3.1 (2.5; 3.9)	59.3	3.0 (1.0)	3.0 (2.3; 3.7)	53.7
Total cholesterol (mmol / L)	5.4 (1.3)	5.3 (4.6; 6.2)	37.9	5.2 (1.2)	5.1 (4.3; 5.9)	30.7
SBP (mmHg)	140 (18)	140 (130; 150)	25.8	137 (17)	136 (127; 146)	20.3

ESM Table 4 Summary of the cut-offs applied to the continuous variables.

Clinical characteristics	Valid data ranges (applied cut-offs)
Age (years)	[18.0, 100.0]
HbA_{1c} (mmol / mol)<br>HbA_{1c} (%)	[20.0, 120.0]<br>[4.0, 13.1]
HDL cholesterol (mmol / L)	[0.0, 20.0]
Total cholesterol (mmol / L)	[0.0, 20.0]
SBP (mmHg)	[70, 250]
DBP (mmHg)	[40, 150]
Urine albumin / creatinine ratio (mg / mmol)	[0.0, 200]
Serum creatinine<br>(micromol / L)	[0.0 - 900]

ESM Table 5 Outcome definitions based on the CALIBER dataset.

Type of cardiovascular disease	Link to the CALIBER research platform
Fatal and non-fatal MI	https://www.caliberresearch.org/portal/show/phenotype_mi<br><br>https://www.caliberresearch.org/portal/show/phenotype_chd_nos
Fatal or non-fatal Stroke	https://www.caliberresearch.org/portal/show/phenotype_stroke_intracerebral_haem,<br>https://www.caliberresearch.org/portal/show/phenotype_stroke_ischaemic,<br>https://www.caliberresearch.org/portal/show/phenotype_stroke_nos,<br>https://www.caliberresearch.org/portal/show/phenotype_stroke_subarachnoid
Fatal peripheral vascular disease	https://www.caliberresearch.org/portal/show/phenotype_pad
Sudden Cardiac Death	https://www.caliberresearch.org/portal/show/phenotype_scd
HF	https://www.caliberresearch.org/portal/show/phenotype_hf
AF	https://www.caliberresearch.org/portal/show/phenotype_af
Ischemic stroke	https://www.caliberresearch.org/portal/show/phenotype_stroke_ischaemic<br><br>https://www.caliberresearch.org/portal/show/phenotype_stroke_nos
Haemorrhagic stroke	https://www.caliberresearch.org/portal/show/phenotype_stroke_intracerebral_haem,<br><br>https://www.caliberresearch.org/portal/show/phenotype_stroke_subarachnoid

ESM Table 6 Baseline characteristics of patients with type 2 diabetes, without pre-existing CVD at the time of diagnosis.

Clinical characteristics	Available sample (%)
Total no. of individuals	168,871
Rheumatoid arthritis	1,746 (1.0)
Renal disease	8,137 (4.8)
Exercise ECG results	3,180 (1.9)
Anticoagulants use	505 (0.3)
Diuretic use	48,154 (28.5)
Corticosteroids use	8,440 (5.0)
Foot amputation	75 (0.0)
Systemic lupus erythematosus	170 (0.1)
HIV positive	116 (0.1)
Mental disorders	1,249 (0.7)
Microalbuminuria	3 (0.0)
Erectile dysfunction disorder	6,485 (3.8)
Erectile dysfunction drugs	5,846 (3.5)
Hypertension	108,924 (64.5)
Migraine	827 (0.5)
Treated hypertension (blood lowering medication)	74,158 (43.9)
Family history of CVD	42,298 (25.0)
Family history of CHD	391 (0.2)
Family history of MI	46 (0.0)
Family history of Stroke	335 (0.2)

ESM Table 7 Ethnic origin of patients with type 2 diabetes.

Clinical characteristics	Available sample (%)
Total no. of individuals	168,871
White British	37,284 (22.1)
White Irish	908 (0.5)
White Other	3,100 (1.8)
White NOS	6,930 (4.1)
Mixed White and Black	311 (0.2)
Mixed White and Asian	131 (0.1)
Mixed Asian and Black	81 (0.0)
Mixed Other	252 (0.1)
Mixed NOS	31 (0.0)
Indian	3,933 (2.3)
Pakistani	2,087 (1.2)
Bangladeshi	831 (0.5)
Other Asian	2,060 (1.2)
Black Caribbean	1,689 (1.0)
Black African	2,497 (1.5)
Black Other	676 (0.4)
Chinese	381 (0.2)
Other Ethnic Group	1,906 (1.1)
Ethnic Group not specified	103,783 (61.5)

ESM Table 8 Characteristics of the evaluated CVD risk scores.

Name	Presented models (number of predictors ESM Table 8)	Year of publication	Derivation sample	Study design	Derived with/ without T2DM individuals	Statistical model	Type of predicted CVD reported	Type of CVD evaluated
FINRISK [10]	Finrisk Stroke (8)<br>Finrisk CHD (8)<br>Finrisk CVD (8)	2016	National FINRISK Study: random population sample of individuals aged 30 to 64 - North Karelia (Finland); n = 9,391 men and 10,056 women	Randomized population sample	with T2DM (diabetes included as a risk factor)	Regression model	CHD (MI, unstable angina pectoris, death from CHD), Stroke (infractions, haemorrhage), CVD	Stroke, CHD, CVD
DARTS (Diabetes Audit and Research in Tayside, Scotland) [11]	DARTS (10)	2006	Tayside, Scotland UK, T2DM without history of CHD	EHR (participants were registered with general practices and the Diabetes Audit and Research in Tayside, Scotland)	with T2DM (100% T2DM individuals)	Weibull AFT model	First major CHD event (fatal or non-fatal acute MI or CHD death)	CHD
Framingham 1991 [12]	Framingham 1991 Fatal CHD (8)<br>Framingham 1991 CVD (8)<br>Framingham 1991 Stroke (8)	1991	5,573 US white men and women aged 30 - 74 years without cancer and CVD	Cohort study: Framingham Heart Study, Framingham Offspring Study	with T2DM (diabetes included as a risk factor)	Weibull AFT model	Myocardial infarction, CHD, fatal CHD, stroke, CVD, death from CVD	CHD, CVD, Stroke
Framingham 1998 [13]	Framingham 1998 (7)	1998	5,345 US white men and women aged 30 - 74	Cohort study: Framingham Heart Study	with T2DM (diabetes included as a risk factor)	Cox's PH model	CHD (angina pectoris, recognised and unrecognised MI, coronary insufficiency, CHD death) Hard CHD included total CHD without angina pectoris	CHD
QRISK 2 [14]	QRISK 2 (13)	2008	2.3 million people, aged 35 - 74, England and Wales, people without previous CVD	EHR (QResearch database)	with T2DM (diabetes included as a risk factor)	Cox's PH model	CHD, stroke, or transient ischemic attack; not peripheral	CVD
QRISK 3 [15]	QRISK 3 (19)	2017	7.89 million people, aged 25 -84 years, without CHD and not prescribed statins	EHR (QResearch general practices in England)	with T2DM (diabetes included as a risk factor)	Cox's PH model	CVD defined as a composite outcome of coronary heart disease, ischaemic stroke, or transient ischaemic attack	CVD
SCORE [16]	SCORE CHD (6)	2003	205,178 European men and women,	Cohort studies,	with T2DM	Weibull PH	CHD, CVD, CVD	CHD, CVD

	SCORE CVD (6)		aged 19 - 80 years without history of MI	mainly carried out in general population settings		model	mortality	
UKPDS 56 [17]	UKPDS 56 (9)	2001	4,540 people from U.K. Prospective Diabetes Study, aged 25 - 65 years, without MI, angina or heart failure	Prospective Diabetes Study	with T2DM (100% T2DM individuals)	Cox's PH model	Fatal or non-fatal MI or sudden death	CHD
UKPDS 68 [18]	UKPDS 68 C-HF (5)<br>UKPDS 68 Stroke (10)	2004	3,641 people with diagnosed type 2 diabetes, aged 25 - 65 years	Prospective Diabetes Study	with T2DM (100% T2DM individuals)	Weibull PH model	CVD: MI (fatal and non-fatal myocardial infarction and sudden death), IHD, CHF, Cerebrovascular disease:<br>first non-fatal stroke	HF, Stroke
UKPDS 82 [19]	UKPDS 82 C-HF (10)<br>UKPDS 82 CHD (15)	2013	5,102 people with diagnosed type 2 diabetes, age 25 - 65 years	Prospective Diabetes Study	with T2DM (100% T2DM individuals)	Weibull PH model	MI: acute myocardial infarction or sudden death; Stroke: non-fatal or fatal stroke; chronic ischemic heart disease (IHD); Congestive heart failure (CHF)	HF, CHD
ASCVD Pooled Cohort Risk Equations [20]	ASCVD (9)	2013	24,626 people, white and African American, aged 40 - 79 years, without CVD	Cohort studies ARIC study, Cardiovascular Health Study, CARDIA, Framingham Original and Offspring Study	with T2DM (diabetes included as a risk factor)	Cox's PH model	ASCVD event (CHD death, nonfatal myocardial infarction, or fatal or nonfatal stroke)	CVD
RECODE [21]	RECODE (13)	2017	9,635 people from Action to Control Cardiovascular Risk in Diabetes Study (ACCORD)	Randomized population sample	with T2DM (100% T2DM individuals)	Cox PH model	MI, stroke, CHF, cardiovascular mortality	CVD + HF
Reynolds Risk ** [22] [23]	Reynolds Risk (9)	2007, 2008	Reynolds Risk Score for Men: 10,724 healthy men without diabetes and CVD, aged over 50 years<br>Reynolds Risk Score for Women: 24,558 healthy women, aged over 45 years	Female: Women's Health Study, a national cohort of US women who passed eligibility criteria<br><br>Male: Physicians Health Study II, a nationwide cohort of US men, passed eligibility criteria	with T2DM (diabetes included as a risk factor)	Cox PH model	CVD, CHD, MI, stroke, coronary revascularization, cardiovascular death	CVD

CHS (Cardiovascular Health Study) * [24]	CHS Basic (8)<br>CHS Advanced (9)	2013	782 people with diabetes, without CVD, CHF and AF, aged over 65 years	Prospective study	with T2DM (100% T2DM individuals)	Cox PH model	MI, stroke and death	CVD

T2DM – type 2 diabetes

*- based on the implementation provided from the Utrecht research team

**- Combined two risk scores: Reynolds Risk Score for Men, Reynolds Risk Score for Women

ESM Table 9 Characteristics of the evaluated CVD risk scores.

Name	Non-linear terms	Interaction effects	Derivation sample characteristics<br>(with/without patients with CVD; patients on/off statins; patients on BP lowering agents)		
			Patients without CVD history	Patients on statins	Patients on/off BP lowering agents
FINRISK [4]	no	no interaction effects<br>separate scores for women/men: different coefficient values, similar form of the score	✔	✔	✔
DARTS (Diabetes Audit and Research in Tayside, Scotland) [5]	log(Duration of diabetes)<br>log(HbA_{1c})	SBP x treated hypertension<br>Log(HbA_{1c}) x follow-up (<=5 vs. > 5 years)<br>no separate scores for women/men	✔	✔	✔
Framingham 1991 [6]	Female/Male: Log(SBP); Log(Total cholesterol/HDL cholesterol); Log(Age); power(Log(age), 2)	Log(Total cholesterol / HDL cholesterol)<br><br>separate scores for women/men: different coefficient values, similar form of the score	✔	✔	✔
Framingham 1998 [7]	Female/ Male: power(Age, 2);	no interaction effects<br><br>separate scores for women/men: different coefficient values, similar form of the score	✔	✔	✔
QRISK 2 [8]	Female: power(Age/10, 0.5); power(BMI/10, -2); log(BMI/10); power(BMI/10, -2)<br><br>Male: power(Age/10, -1); power(BMI/10, -2); power(BMI/10, -2); log(BMI/10)	Female:<br>Age/10 x (Smoking status; AF; CKD; Treated Hypertension; Type 1 diabetes; Type 2 diabetes; power(BMI, -2); power(BMI, -2) x Log(BMI/10); Family history of CVD; SBP; Townsend Score)<br><br>power(Age/10, 0.5) x (Smoking status; AF; CKD; Treated Hypertension; Type 1 diabetes; Type 2 diabetes; power(BMI, -2); power(BMI, -2) x Log(BMI/10); Family history of CVD; SBP; Townsend Score)<br><br>Male:<br>power(Age/10, -1) x (Smoking status; AF; CKD; Treated Hypertension; Type 1 diabetes; Type 2 diabetes; power(BMI, -2); power(BMI, -2) x Log(BMI/10); Family history of CVD; SBP; Townsend Score)<br><br>power(Age/10, 2) x (Smoking status; AF; CKD; Treated Hypertension; Type 1 diabetes; Type 2 diabetes; power(BMI, -2); power(BMI, -2) x Log(BMI/10); Family history of CVD; SBP; Townsend Score)	✔		✔
QRISK 3 [9]	Female: power(Age/10, -2); power(BMI/10, -2); power(BMI/10, -2) x log(BMI/10)	Female:<br>power(Age/10, -2) x (Smoking status; AF; Corticosteroids use; Migraine; CKD; Systemic lupus; Treated hypertension; Type 1 diabetes; Type 2 diabetes, power(BMI, -2) x log(BMI/10); power(BMI, -2); Family history of CVD; SBP; Townsend Score)	✔		✔

	Male: power(Age/10, -1); power(Age/10, 3); power(BMI/10, -2) x log(BMI/10); power(BMI/10, -2)	Age/10 x (Smoking status; AF; Corticosteroids use; Migraine; CKD; Systemic lupus; Treated hypertension; Type 1 diabetes; Type 2 diabetes; power(BMI, -2) x log(BMI/10); power(BMI, -2); Family history of CVD; SBP; Townsend Score)<br><br>Male:<br>power(Age/10, -1) x (Smoking status; AF; Corticosteroids use; Migraine; CKD; Systemic lupus; Treated hypertension; Type 1 diabetes; Type 2 diabetes; power(BMI, -2) x log(BMI/10); power(BMI, -2); Family history of CVD; SBP; Townsend Score)<br><br>power(Age/10, 3) x (Smoking status; AF; Corticosteroids use; Migraine; CKD; Systemic lupus; Treated hypertension; Type 1 diabetes; Type 2 diabetes; power(BMI, -2) x log(BMI/10); power(BMI, -2); Family history of CVD; SBP; Townsend Score)<br><br>separate scores for women/men			
SCORE [10]	power(age - 10, p); power(age - 20, p) where p is from 4.62 to 6.42	no interaction effects<br><br>no separate scores for women/men, only different model parameters for women/men	✔	✔	✔
UKPDS 56 [11]	Log(Total cholesterol/HDL cholesterol); Total cholesterol / HDL cholesterol	Log(Total cholesterol/HDL cholesterol); Total cholesterol / HDL cholesterol<br><br>no separate scores for women/men	✔	✔	✔
UKPDS 68 [12]	Log(Total cholesterol / HDL cholesterol); Total cholesterol / HDL cholesterol	Log(Total cholesterol/HDL cholesterol)<br><br>no separate scores for women/men	✔	✔	✔
UKPDS 82 [13]	no	no interaction effects<br><br>no separate scores for women/men	✔	✔	✔
ASCVD Pooled Cohort Risk Equations [14]	Female/Male: Log(Age); power(Log(Age), 2); Log(Total cholesterol); Log(HDL cholesterol); Log(SBP); Log(Treated SBP)	separate scores for women/men, and for White and African American<br><br>Log(Age) x Log(Total cholesterol); Log(Age) x Log(HDL cholesterol); Log(Age) x Log(Treated SBP); Log(Age) x Log(Untreated SBP); Log(Age) x Smoking status	✔	✔	✔
RECODE [15]	no non-linear terms	no interaction effects<br>no separate scores for women/men		✔	✔

ESM Table 10 Predictor variables included by the considered cardiovascular prediction models. (*) Type 2 diabetes either refers to type 2 diabetes as a risk factor or to a type 2 diabetes derivation population for a risk score.

	DARTS [5](a)	UKPDS 56 [11](a)	UKPDS 68 [12](a)	UKPDS 82 [13](a)	RECODE [15](a)	CHS [18](a)	FINRISK [4](b)	Framingham 1991 [6] (b)	Framingham 1998 [7](b)	QRISK 2 [8](b)	QRISK 3 [9](b)	ASCVD [14](b)	Reynolds Risk [16][17](b)	SCORE [10](c)	**Number of publications**
**Age**	✔	✔	✔	✔	✔	✔	✔	✔	✔	✔	✔	✔	✔	✔	14
**Sex**	✔	✔	✔	✔	✔		✔	✔	✔	✔	✔	✔	✔	✔	13
**Smoking status**	✔	✔	✔	✔	✔	✔	✔	✔	✔	✔	✔	✔	✔	✔	14
**SBP or DBP**	✔	✔	✔	✔	✔	✔	✔	✔	✔	✔	✔	✔	✔	✔	14
**Total cholesterol**	✔	✔	✔		✔	✔	✔	✔	✔	✔	✔	✔	✔	✔	13
**HDL cholesterol**		✔	✔	✔	✔	✔	✔	✔	✔	✔		✔	✔	✔	12
**LDL cholesterol**				✔											1
**Type 2 diabetes***	✔	✔	✔	✔	✔	✔	✔	✔	✔	✔	✔	✔	✔		13
**Duration of diabetes**	✔			✔											2
**HbA$_{1c}$**	✔	✔	✔	✔	✔										5
**Treated hypertension**	✔				✔					✔	✔	✔			5
**Height**	✔														1
**Left Ventricular Hypertrophy (LVH) ECG**								✔							1
**Ethnicity**		✔		✔	✔					✔	✔	✔			6
**BMI**			✔	✔						✔					3

**AF**			✔	✔						✔	✔				4
**Townsend Social Deprivation Score**										✔	✔				2
**Rheumatoid Arthritis (RA)**										✔	✔				2
**Family history of CVD**											✔		✔		2
**Type 1 diabetes**											✔				1
**Chronic kidney disease (CKD)**											✔				1
**Migraine**											✔				1
**Corticosteroid use**											✔				1
**Systemic lupus**											✔				1
**Atypical antipsychotic**											✔				1
**Severe mental illness**											✔				1
**History of CVD**			✔	✔	✔		✔								4
**Peripheral vascular disease (PVD)**				✔											1
**White Blood Count (WBC)**				✔											1
**Presence of micro- or macro albuminuria**				✔											1
**Serum Creatinine**					✔	✔									2
**Urine albumin to creatinine ratio**					✔										1

**High-sensitivity C-reactive protein (hsCRP)**						✔							✔		2
**Use of glucose-lowering medications**						✔									1
**Use of statins or anticoagulants**					✔										1
**Total number of predictors**	10	9	11	16	13	9	8	8	7	13	19	9	9	6	

n.b Derivation population: (a) Population with type 2 diabetes, (b) General population, (c) General population excluding individuals with type 2 diabetes

ESM Table 11 The 10-year incidence of cardiovascular disease after a diagnosis of type 2 diabetes.

Type of cardiovascular disease	No. events (%)
Total no. of individuals	168,871
CVD, HF, or AF	38,335 (22.70)
CVD	29,025 (17.19)
CHD	20,628 (12.22)
Any stroke	6,727 (3.98)
HF	9,465 (5.6)
AF	13,826 (8.19)

n.b. One patient could have more than one event, but the event type is unique.

ESM Table 12 Model calibration (calibration-in-the-large (CIL) and calibration slope (CS)) based on a complete-cases analysis, predicting 10-years risk of CVD in type 2 diabetes patients.

	CVD		CHD		CVD + AF + HF		Stroke		AF		HF	
	CS	CIL	CS	CIL	CS	CIL	CS	CIL	CS	CIL	CS	CIL
ASCVD	0.437 (0.420;0.454)	-0.862 (-0.885;-0.838)	0.365 (0.347;0.384)	-1.320 (-1.347;-1.293)	0.516 (0.500;0.532)	-0.415 (-0.436;-0.394)	0.453 (0.423;0.482)	-2.866 (-2.913;-2.819)	0.528 (0.506;0.550)	-1.800 (-1.831;-1.768)	0.514 (0.488;0.539)	-2.355 (-2.394;-2.317)
CHS Advanced	0.617 (0.592;0.643)	-0.734 (-0.757;-0.712)	0.524 (0.496;0.552)	-1.142 (-1.168;-1.116)	0.760 (0.736;0.783)	-0.336 (-0.356;-0.316)	0.744 (0.694;0.794)	-2.527 (-2.572;-2.483)	0.941 (0.906;0.976)	-1.582 (-1.612;-1.552)	0.879 (0.837;0.921)	-2.074 (-2.110;-2.037)
CHS Basic	0.721 (0.691;0.751)	-0.810 (-0.833;-0.788)	0.617 (0.584;0.650)	-1.208 (-1.234;-1.183)	0.885 (0.857;0.912)	-0.423 (-0.443;-0.403)	0.857 (0.799;0.916)	-2.571 (-2.615;-2.527)	1.099 (1.057;1.140)	-1.639 (-1.669;-1.609)	1.022 (0.973;1.071)	-2.123 (-2.159;-2.087)
DARTS	0.585 (0.542;0.628)	-1.168 (-1.208;-1.129)	0.497 (0.449;0.544)	-1.578 (-1.623;-1.533)	0.646 (0.606;0.686)	-0.777 (-0.813;-0.742)	0.621 (0.544;0.699)	-2.940 (-3.017;-2.863)	0.609 (0.553;0.666)	-2.066 (-2.119;-2.012)	0.654 (0.588;0.721)	-2.519 (-2.584;-2.455)
Finrisk CHD	0.535 (0.515;0.555)	0.246 (0.223;0.268)	0.452 (0.430;0.474)	-0.177 (-0.202;-0.151)	0.593 (0.575;0.612)	0.661 (0.640;0.681)	0.631 (0.590;0.671)	-1.603 (-1.646;-1.559)	0.641 (0.613;0.668)	-0.642 (-0.672;-0.612)	0.657 (0.624;0.691)	-1.152 (-1.188;-1.115)
Finrisk CVD	0.560 (0.539;0.580)	0.260 (0.238;0.283)	0.474 (0.452;0.497)	-0.161 (-0.187;-0.136)	0.636 (0.618;0.655)	0.674 (0.654;0.695)	0.654 (0.613;0.695)	-1.584 (-1.628;-1.541)	0.715 (0.686;0.743)	-0.626 (-0.656;-0.596)	0.706 (0.672;0.740)	-1.134 (-1.170;-1.098)
Finrisk Stroke	0.492 (0.473;0.511)	0.283 (0.260;0.305)	0.416 (0.395;0.437)	-0.151 (-0.176;-0.125)	0.576 (0.558;0.593)	0.707 (0.687;0.728)	0.562 (0.526;0.598)	-1.611 (-1.655;-1.567)	0.662 (0.637;0.688)	-0.628 (-0.658;-0.598)	0.627 (0.597;0.658)	-1.150 (-1.187;-1.114)
Framingham 1991 CVD	0.571 (0.546;0.595)	-2.076 (-2.098;-2.054)	0.497 (0.470;0.525)	-2.479 (-2.504;-2.454)	0.575 (0.553;0.597)	-1.679 (-1.698;-1.659)	0.525 (0.476;0.574)	-3.868 (-3.911;-3.824)	0.481 (0.449;0.514)	-2.921 (-2.951;-2.892)	0.558 (0.517;0.598)	-3.415 (-3.451;-3.380)
Framingham 1991 fatal CHD	0.577 (0.552;0.601)	0.206 (0.184;0.228)	0.498 (0.470;0.525)	-0.193 (-0.218;-0.168)	0.593 (0.571;0.615)	0.600 (0.580;0.620)	0.603 (0.551;0.655)	-1.569 (-1.612;-1.526)	0.570 (0.536;0.604)	-0.630 (-0.659;-0.601)	0.642 (0.598;0.685)	-1.120 (-1.155;-1.084)
Framingham 1991 Stroke	0.400 (0.384;0.416)	-0.377 (-0.401;-0.354)	0.329 (0.311;0.347)	-0.831 (-0.857;-0.804)	0.447 (0.432;0.462)	0.069 (0.048;0.090)	0.403 (0.374;0.432)	-2.392 (-2.438;-2.346)	0.418 (0.397;0.439)	-1.328 (-1.359;-1.297)	0.450 (0.425;0.475)	-1.884 (-1.922;-1.846)
Framingham 1998	0.785 (0.755;0.815)	-0.321 (-0.342;-0.299)	0.644 (0.611;0.678)	-0.714 (-0.739;-0.689)	0.923 (0.895;0.951)	0.065 (0.045;0.084)	0.979 (0.919;1.039)	-2.082 (-2.125;-2.039)	1.061 (1.019;1.102)	-1.147 (-1.176;-1.118)	1.087 (1.036;1.137)	-1.634 (-1.670;-1.599)
QRISK 2	0.689 (0.660;0.719)	-0.854 (-0.878;-0.829)	0.594 (0.562;0.627)	-1.249 (-1.277;-1.221)	0.775 (0.748;0.802)	-0.469 (-0.491;-0.447)	0.674 (0.619;0.729)	-2.663 (-2.712;-2.614)	0.767 (0.727;0.806)	-1.720 (-1.753;-1.687)	0.783 (0.736;0.831)	-2.215 (-2.255;-2.174)
QRISK 3	0.629 (0.580;0.678)	-0.887 (-0.928;-0.846)	0.523 (0.470;0.577)	-1.276 (-1.323;-1.230)	0.736 (0.690;0.782)	-0.490 (-0.528;-0.453)	0.555 (0.464;0.645)	-2.752 (-2.837;-2.668)	0.735 (0.669;0.801)	-1.728 (-1.784;-1.673)	0.735 (0.657;0.812)	-2.200 (-2.267;-2.133)

RECODE	0.816 (0.725;0.907)	-0.459 (-0.516;-0.402)	0.730 (0.626;0.835)	-0.895 (-0.962;-0.828)	0.907 (0.823;0.990)	-0.068 (-0.119;-0.018)	0.776 (0.609;0.942)	-2.085 (-2.195;-1.975)	0.899 (0.780;1.018)	-1.250 (-1.327;-1.173)	0.953 (0.813;1.093)	-1.674 (-1.766;-1.582)
Reynolds Risk	0.082 (0.074;0.089)	0.656 (0.631;0.682)	0.087 (0.078;0.095)	0.210 (0.181;0.238)	0.074 (0.068;0.081)	1.104 (1.081;1.128)	0.038 (0.023;0.052)	-1.314 (-1.362;-1.265)	0.066 (0.056;0.076)	-0.316 (-0.350;-0.283)	0.045 (0.033;0.058)	-0.852 (-0.892;-0.811)
SCORE CHD	0.452 (0.436;0.468)	2.127 (2.107;2.147)	0.381 (0.364;0.399)	1.699 (1.676;1.722)	0.504 (0.489;0.518)	2.548 (2.529;2.566)	0.545 (0.511;0.579)	0.255 (0.217;0.294)	0.587 (0.564;0.610)	1.216 (1.189;1.243)	0.595 (0.567;0.624)	0.725 (0.693;0.757)
SCORE CVD	0.434 (0.419;0.449)	1.516 (1.495;1.536)	0.361 (0.344;0.377)	1.079 (1.056;1.102)	0.497 (0.483;0.511)	1.945 (1.927;1.964)	0.540 (0.508;0.571)	-0.390 (-0.429;-0.351)	0.590 (0.568;0.612)	0.587 (0.560;0.614)	0.598 (0.571;0.624)	0.088 (0.055;0.120)
UKPDS 56	0.490 (0.464;0.517)	-0.560 (-0.588;-0.532)	0.439 (0.410;0.468)	-0.972 (-1.004;-0.940)	0.504 (0.480;0.528)	-0.162 (-0.188;-0.137)	0.452 (0.403;0.501)	-2.415 (-2.471;-2.359)	0.468 (0.434;0.503)	-1.485 (-1.524;-1.447)	0.493 (0.452;0.534)	-1.955 (-2.001;-1.908)
UKPDS 68 C-HF	0.267 (0.194;0.339)	0.188 (0.133;0.242)	0.237 (0.155;0.320)	-0.321 (-0.383;-0.258)	0.397 (0.329;0.464)	0.748 (0.699;0.798)	0.200 (0.075;0.325)	-1.473 (-1.567;-1.379)	0.419 (0.335;0.502)	-0.389 (-0.453;-0.325)	0.556 (0.460;0.651)	-0.871 (-0.946;-0.796)
UKPDS 68 Stroke	0.267 (0.194;0.339)	0.188 (0.133;0.242)	0.237 (0.155;0.320)	-0.321 (-0.383;-0.258)	0.397 (0.329;0.464)	0.748 (0.699;0.798)	0.200 (0.075;0.325)	-1.473 (-1.567;-1.379)	0.419 (0.335;0.502)	-0.389 (-0.453;-0.325)	0.556 (0.460;0.651)	-0.871 (-0.946;-0.796)
UKPDS 82 C-HF	0.450 (0.409;0.490)	-0.060 (-0.101;-0.018)	0.412 (0.366;0.458)	-0.465 (-0.512;-0.418)	0.581 (0.544;0.618)	0.350 (0.313;0.387)	0.465 (0.381;0.548)	-1.887 (-1.970;-1.804)	0.778 (0.719;0.836)	-0.907 (-0.963;-0.852)	0.819 (0.749;0.890)	-1.369 (-1.436;-1.302)
UKPDS 82 CHD	0.447 (0.406;0.489)	-0.626 (-0.667;-0.585)	0.387 (0.340;0.434)	-1.031 (-1.078;-0.984)	0.518 (0.480;0.555)	-0.217 (-0.253;-0.180)	0.481 (0.396;0.565)	-2.454 (-2.538;-2.371)	0.583 (0.526;0.640)	-1.474 (-1.529;-1.418)	0.664 (0.596;0.733)	-1.936 (-2.002;-1.869)

ESM Table 13 Calibration (calibration-in-the-large (CIL) and calibration slope (CS)) after model recalibration, based on a complete-cases analysis, predicting 10-years risk of CVD in type 2 diabetes patients.

	CVD		CHD		CVD + AF + HF		Stroke		AF		HF	
	CS	CIL	CS	CIL	CS	CIL	CS	CIL	CS	CIL	CS	CIL
ASCVD	0.982 (0.944;1.020)	-0.014 (-0.036;0.007)	0.969 (0.920;1.018)	0.012 (-0.013;0.037)	1.055 (1.022;1.088)	-0.016 (-0.035;0.004)	0.939 (0.878;1.000)	-0.137 (-0.181;-0.093)	1.091 (1.046;1.137)	-0.084 (-0.114;-0.055)	0.973 (0.925;1.022)	-0.005 (-0.041;0.031)
CHS Advanced	1.006 (0.964;1.047)	-0.011 (-0.033;0.011)	1.058 (1.001;1.116)	-0.021 (-0.046;0.004)	0.979 (0.949;1.010)	-0.001 (-0.021;0.019)	0.947 (0.884;1.010)	-0.067 (-0.111;-0.023)	0.973 (0.937;1.010)	0.023 (-0.007;0.053)	0.932 (0.888;0.976)	-0.022 (-0.058;0.015)
CHS Basic	1.004 (0.963;1.045)	-0.011 (-0.033;0.011)	1.059 (1.002;1.116)	-0.021 (-0.046;0.004)	0.978 (0.947;1.008)	-0.001 (-0.021;0.018)	0.943 (0.878;1.007)	-0.067 (-0.111;-0.023)	0.975 (0.938;1.011)	0.022 (-0.008;0.052)	0.934 (0.889;0.979)	-0.022 (-0.058;0.014)
DARTS	0.981 (0.908;1.053)	-0.026 (-0.064;0.012)	0.904 (0.817;0.991)	0.010 (-0.034;0.054)	1.012 (0.949;1.074)	-0.048 (-0.082;-0.013)	1.388 (1.215;1.561)	-0.067 (-0.142;0.009)	1.039 (0.943;1.136)	-0.053 (-0.105;-0.000)	1.023 (0.919;1.127)	-0.060 (-0.123;0.003)
Finrisk CHD	1.034 (0.996;1.073)	-0.037 (-0.058;-0.016)	1.011 (0.962;1.061)	-0.027 (-0.051;-0.003)	1.021 (0.989;1.052)	-0.010 (-0.029;0.009)	1.227 (1.149;1.304)	-0.033 (-0.076;0.010)	1.061 (1.016;1.107)	0.059 (0.030;0.088)	0.874 (0.830;0.919)	0.046 (0.010;0.082)
Finrisk CVD	1.057 (1.018;1.095)	-0.037 (-0.059;-0.016)	1.031 (0.982;1.080)	-0.027 (-0.051;-0.003)	1.039 (1.008;1.070)	-0.011 (-0.030;0.009)	1.198 (1.124;1.273)	-0.033 (-0.076;0.010)	1.072 (1.030;1.114)	0.059 (0.030;0.088)	0.908 (0.864;0.952)	0.047 (0.011;0.083)
Finrisk Stroke	1.078 (1.037;1.119)	-0.037 (-0.058;-0.016)	1.052 (0.999;1.104)	-0.027 (-0.051;-0.003)	1.054 (1.023;1.086)	-0.011 (-0.030;0.009)	1.147 (1.073;1.221)	-0.033 (-0.075;0.010)	1.066 (1.025;1.107)	0.059 (0.030;0.088)	0.934 (0.888;0.979)	0.047 (0.012;0.083)
Framingham 1991 CVD	1.082 (1.035;1.129)	-0.053 (-0.074;-0.032)	1.081 (1.020;1.141)	-0.041 (-0.065;-0.016)	1.057 (1.016;1.097)	0.005 (-0.014;0.024)	1.069 (0.969;1.169)	-0.110 (-0.152;-0.067)	0.946 (0.882;1.010)	0.099 (0.070;0.127)	0.859 (0.797;0.921)	0.087 (0.052;0.122)
Framingham 1991 fatal CHD	1.091 (1.044;1.138)	-0.055 (-0.076;-0.033)	1.080 (1.021;1.140)	-0.042 (-0.066;-0.018)	1.066 (1.027;1.106)	0.003 (-0.016;0.022)	1.031 (0.942;1.120)	-0.112 (-0.155;-0.069)	0.969 (0.911;1.026)	0.097 (0.068;0.125)	0.818 (0.763;0.873)	0.084 (0.048;0.119)
Framingham 1991 Stroke	1.098 (1.053;1.143)	-0.056 (-0.077;-0.035)	1.177 (1.113;1.241)	-0.042 (-0.066;-0.018)	1.068 (1.032;1.105)	0.001 (-0.018;0.020)	1.044 (0.968;1.120)	-0.111 (-0.154;-0.068)	0.988 (0.938;1.037)	0.097 (0.068;0.126)	0.955 (0.903;1.008)	0.086 (0.051;0.122)
Framingham 1998	1.082 (1.041;1.123)	-0.063 (-0.085;-0.042)	1.102 (1.045;1.159)	-0.048 (-0.072;-0.024)	1.047 (1.016;1.079)	-0.008 (-0.027;0.012)	1.018 (0.956;1.081)	-0.128 (-0.171;-0.084)	0.942 (0.905;0.979)	0.082 (0.053;0.111)	0.916 (0.874;0.959)	0.067 (0.032;0.103)
QRISK 2	0.982 (0.940;1.024)	0.001 (-0.023;0.025)	0.930 (0.880;0.981)	0.026 (-0.001;0.053)	1.003 (0.968;1.038)	0.020 (-0.001;0.042)	1.007 (0.925;1.089)	0.005 (-0.044;0.053)	0.975 (0.924;1.025)	0.047 (0.015;0.080)	0.993 (0.933;1.054)	-0.008 (-0.048;0.032)
QRISK 3	0.930	-0.018 (-	0.868	0.075	0.941	0.010 (-	0.710	-0.161 (-0.245;-	0.973	-0.036 (-	1.022	0.113

	(0.858;1.003)	0.059;0.022)	(0.779;0.957)	(0.029;0.121)	(0.883;1.000)	0.027;0.046)	(0.595;0.826)	0.077)	(0.886;1.060)	0.091;0.018)	(0.914;1.130)	(0.047;0.179)
RECODE	1.182 (1.050;1.315)	0.066 (0.009;0.122)	1.233 (1.057;1.409)	-0.013 (-0.079;0.053)	1.046 (0.950;1.143)	0.108 (0.058;0.159)	0.819 (0.643;0.994)	0.112 (0.002;0.222)	0.976 (0.846;1.105)	0.162 (0.085;0.239)	1.041 (0.888;1.194)	0.264 (0.172;0.356)
Reynolds Risk	0.819 (0.745;0.893)	-0.011 (-0.034;0.012)	0.952 (0.858;1.046)	-0.027 (-0.053;-0.001)	0.833 (0.760;0.905)	0.013 (-0.007;0.034)	1.708 (1.043;2.373)	0.068 (0.021;0.115)	0.908 (0.768;1.047)	0.046 (0.015;0.077)	0.808 (0.592;1.025)	-0.007 (-0.046;0.031)
SCORE CHD	0.940 (0.907;0.973)	0.035 (0.016;0.054)	0.899 (0.857;0.940)	0.062 (0.041;0.084)	0.962 (0.934;0.990)	0.043 (0.026;0.060)	1.159 (1.086;1.231)	-0.027 (-0.065;0.011)	0.991 (0.952;1.031)	0.067 (0.042;0.093)	0.969 (0.922;1.015)	0.094 (0.063;0.126)
SCORE CVD	0.937 (0.904;0.969)	0.035 (0.016;0.054)	0.897 (0.856;0.938)	0.062 (0.041;0.084)	0.960 (0.933;0.987)	0.043 (0.026;0.061)	1.150 (1.083;1.218)	-0.027 (-0.065;0.012)	1.000 (0.963;1.038)	0.068 (0.042;0.094)	0.975 (0.931;1.018)	0.095 (0.064;0.127)
UKPDS 56	1.301 (1.231;1.371)	0.041 (0.014;0.067)	1.310 (1.222;1.397)	0.054 (0.024;0.085)	1.148 (1.093;1.203)	0.013 (-0.011;0.037)	1.129 (1.006;1.251)	0.041 (-0.013;0.096)	1.120 (1.037;1.202)	-0.034 (-0.071;0.003)	0.942 (0.864;1.020)	-0.012 (-0.057;0.033)
UKPDS 68 C-HF	1.116 (0.811;1.420)	0.032 (-0.020;0.084)	0.807 (0.526;1.088)	0.070 (0.010;0.130)	1.301 (1.080;1.521)	0.036 (-0.011;0.083)	0.747 (0.281;1.213)	-0.108 (-0.200;-0.016)	2.563 (2.053;3.073)	0.030 (-0.031;0.092)	0.998 (0.826;1.169)	0.073 (0.000;0.146)
UKPDS 68 Stroke	1.116 (0.811;1.420)	0.032 (-0.020;0.084)	0.807 (0.526;1.088)	0.070 (0.010;0.130)	1.301 (1.080;1.521)	0.036 (-0.011;0.083)	0.747 (0.281;1.213)	-0.108 (-0.200;-0.016)	2.563 (2.053;3.073)	0.030 (-0.031;0.092)	0.998 (0.826;1.169)	0.073 (0.000;0.146)
UKPDS 82 C-HF	0.805 (0.733;0.878)	-0.021 (-0.061;0.019)	0.876 (0.778;0.974)	0.007 (-0.038;0.053)	0.814 (0.762;0.866)	0.032 (-0.004;0.068)	0.694 (0.570;0.818)	-0.009 (-0.091;0.074)	0.877 (0.811;0.943)	0.009 (-0.046;0.064)	0.832 (0.761;0.904)	0.134 (0.067;0.201)
UKPDS 82 CHD	1.065 (0.966;1.165)	-0.029 (-0.069;0.010)	1.358 (1.192;1.523)	0.000 (-0.045;0.045)	0.979 (0.908;1.050)	0.020 (-0.015;0.056)	0.672 (0.554;0.789)	-0.033 (-0.115;0.050)	0.945 (0.853;1.036)	-0.015 (-0.069;0.039)	0.975 (0.874;1.075)	0.096 (0.030;0.161)

ESM Table 14 Model calibration (calibration-in-the-large (CIL) and calibration slope (CS)) using the imputed datasets, predicting 10-years risk of CVD in type 2 diabetes patients.

Risk Score Name	**CVD**		**CHD**		**CVD + AF + HF**		**Stroke**		**AF**		**HF**	
	**CS**	**CIL**	**CS**	**CIL**	**CS**	**CIL**	**CS**	**CIL**	**CS**	**CIL**	**CS**	**CIL**
ASCVD	0.383 (0.373;0.393)	-0.639 (-0.656;-0.622)	0.31 (0.299;0.321)	-1.144 (-1.163;-1.125)	0.461 (0.451;0.471)	-0.191 (-0.207;-0.176)	0.405 (0.387;0.423)	-2.636 (-2.665;-2.607)	0.454 (0.44;0.468)	-1.701 (-1.722;-1.679)	0.449 (0.434;0.465)	-2.195 (-2.22;-2.17)
CHS Advanced	0.595 (0.58;0.61)	-0.53 (-0.545;-0.516)	0.491 (0.475;0.508)	-0.978 (-0.994;-0.961)	0.731 (0.717;0.745)	-0.134 (-0.147;-0.12)	0.752 (0.724;0.779)	-2.287 (-2.313;-2.26)	0.84 (0.819;0.86)	-1.469 (-1.488;-1.449)	0.824 (0.8;0.848)	-1.903 (-1.926;-1.88)
CHS Basic	0.699 (0.682;0.716)	-0.607 (-0.621;-0.593)	0.58 (0.561;0.6)	-1.041 (-1.057;-1.024)	0.856 (0.839;0.872)	-0.224 (-0.237;-0.211)	0.878 (0.845;0.911)	-2.32 (-2.347;-2.294)	0.988 (0.964;1.013)	-1.519 (-1.538;-1.5)	0.967 (0.938;0.995)	-1.944 (-1.966;-1.921)
DARTS	0.5 (0.48;0.52)	-1.049 (-1.066;-1.032)	0.401 (0.377;0.426)	-1.504 (-1.523;-1.484)	0.585 (0.567;0.603)	-0.65 (-0.665;-0.634)	0.499 (0.453;0.544)	-2.855 (-2.884;-2.825)	0.529 (0.501;0.557)	-2.007 (-2.029;-1.985)	0.543 (0.502;0.584)	-2.455 (-2.481;-2.429)
Finrisk CHD	0.475 (0.462;0.488)	0.401 (0.383;0.418)	0.398 (0.384;0.412)	-0.073 (-0.092;-0.053)	0.542 (0.53;0.554)	0.824 (0.807;0.841)	0.568 (0.544;0.592)	-1.434 (-1.462;-1.405)	0.575 (0.557;0.593)	-0.588 (-0.609;-0.566)	0.598 (0.577;0.619)	-1.038 (-1.063;-1.013)
Finrisk CVD	0.509 (0.496;0.521)	0.437 (0.42;0.453)	0.426 (0.412;0.44)	-0.033 (-0.051;-0.015)	0.589 (0.577;0.601)	0.857 (0.842;0.872)	0.608 (0.584;0.632)	-1.385 (-1.413;-1.357)	0.64 (0.623;0.658)	-0.545 (-0.566;-0.524)	0.651 (0.63;0.672)	-0.992 (-1.016;-0.968)
Finrisk Stroke	0.462 (0.451;0.474)	0.488 (0.472;0.504)	0.387 (0.374;0.4)	0.006 (-0.012;0.024)	0.546 (0.535;0.557)	0.917 (0.902;0.932)	0.542 (0.521;0.564)	-1.383 (-1.411;-1.355)	0.599 (0.583;0.615)	-0.519 (-0.54;-0.499)	0.591 (0.573;0.61)	-0.98 (-1.004;-0.956)
Framingham 1991 CVD	0.498 (0.48;0.515)	-1.888 (-1.906;-1.869)	0.433 (0.414;0.452)	-2.33 (-2.35;-2.31)	0.521 (0.506;0.536)	-1.495 (-1.513;-1.478)	0.478 (0.448;0.508)	-3.626 (-3.655;-3.596)	0.468 (0.445;0.49)	-2.816 (-2.839;-2.793)	0.519 (0.49;0.547)	-3.246 (-3.272;-3.22)
Framingham 1991 fatal CHD	0.49 (0.473;0.508)	0.391 (0.374;0.409)	0.424 (0.406;0.442)	-0.047 (-0.067;-0.028)	0.526 (0.511;0.541)	0.782 (0.765;0.799)	0.553 (0.52;0.587)	-1.329 (-1.357;-1.301)	0.544 (0.52;0.568)	-0.528 (-0.55;-0.506)	0.596 (0.567;0.625)	-0.953 (-0.978;-0.928)
Framingham 1991 Stroke	0.359 (0.347;0.371)	-0.165 (-0.185;-0.144)	0.292 (0.279;0.304)	-0.668 (-0.691;-0.644)	0.41 (0.399;0.421)	0.28 (0.261;0.298)	0.368 (0.349;0.387)	-2.144 (-2.18;-2.108)	0.381 (0.367;0.395)	-1.221 (-1.248;-1.194)	0.404 (0.387;0.421)	-1.711 (-1.742;-1.679)
Framingham 1998	0.738 (0.719;0.756)	-0.147 (-0.162;-0.132)	0.599 (0.579;0.619)	-0.579 (-0.596;-0.562)	0.877 (0.86;0.895)	0.234 (0.22;0.248)	0.939 (0.904;0.973)	-1.854 (-1.881;-1.827)	0.965 (0.94;0.991)	-1.055 (-1.075;-1.035)	1.003 (0.972;1.033)	-1.478 (-1.501;-1.455)
QRISK2	0.595 (0.576;0.613)	-0.64 (-0.655;-0.624)	0.505 (0.483;0.527)	-1.083 (-1.1;-1.066)	0.686 (0.67;0.703)	-0.248 (-0.263;-0.234)	0.614 (0.57;0.658)	-2.389 (-2.417;-2.362)	0.688 (0.659;0.717)	-1.571 (-1.592;-1.551)	0.71 (0.67;0.75)	-2.005 (-2.028;-1.981)
QRISK3	0.63 (0.611;0.65)	-0.436 (-0.452;-0.421)	0.532 (0.508;0.556)	-0.875 (-0.892;-0.858)	0.723 (0.706;0.741)	-0.049 (-0.063;-0.034)	0.652 (0.603;0.7)	-2.172 (-2.199;-2.145)	0.715 (0.684;0.746)	-1.359 (-1.379;-1.339)	0.74 (0.697;0.783)	-1.79 (-1.813;-1.766)

RECODE	0.731 (0.704;0.759)	-0.2 (-0.214;-0.185)	0.626 (0.598;0.654)	-0.622 (-0.638;-0.606)	0.845 (0.82;0.87)	0.171 (0.158;0.184)	0.747 (0.701;0.793)	-1.885 (-1.911;-1.858)	0.854 (0.821;0.886)	-1.092 (-1.111;-1.072)	0.847 (0.806;0.889)	-1.511 (-1.533;-1.488)
Reynolds Risk	0.461 (0.448;0.473)	0.401 (0.384;0.419)	0.388 (0.374;0.401)	-0.074 (-0.093;-0.054)	0.522 (0.511;0.534)	0.824 (0.808;0.841)	0.526 (0.503;0.549)	-1.448 (-1.478;-1.419)	0.539 (0.521;0.557)	-0.592 (-0.614;-0.57)	0.553 (0.533;0.573)	-1.047 (-1.073;-1.022)
SCORE CHD	0.416 (0.405;0.427)	2.256 (2.24;2.273)	0.356 (0.344;0.368)	1.781 (1.763;1.799)	0.468 (0.458;0.479)	2.685 (2.67;2.701)	0.511 (0.489;0.533)	0.424 (0.396;0.452)	0.54 (0.524;0.556)	1.267 (1.246;1.288)	0.546 (0.527;0.566)	0.818 (0.794;0.842)
SCORE CVD	0.4 (0.39;0.411)	1.647 (1.631;1.664)	0.337 (0.325;0.348)	1.161 (1.143;1.179)	0.46 (0.45;0.47)	2.087 (2.072;2.102)	0.504 (0.484;0.525)	-0.224 (-0.252;-0.196)	0.534 (0.519;0.549)	0.635 (0.614;0.656)	0.542 (0.524;0.56)	0.177 (0.153;0.201)
UKPDS 56	0.446 (0.431;0.46)	-0.386 (-0.405;-0.367)	0.383 (0.368;0.398)	-0.851 (-0.872;-0.829)	0.488 (0.475;0.501)	0.024 (0.007;0.042)	0.448 (0.422;0.473)	-2.217 (-2.249;-2.185)	0.461 (0.442;0.48)	-1.363 (-1.388;-1.338)	0.471 (0.448;0.494)	-1.816 (-1.845;-1.788)
UKPDS 68 C-HF	0.389 (0.376;0.402)	0.877 (0.853;0.9)	0.321 (0.308;0.334)	0.392 (0.366;0.418)	0.489 (0.475;0.502)	1.31 (1.289;1.331)	0.511 (0.489;0.533)	-1.001 (-1.04;-0.962)	0.584 (0.565;0.603)	-0.135 (-0.165;-0.105)	0.598 (0.574;0.622)	-0.596 (-0.631;-0.562)
UKPDS 68 Stroke	0.389 (0.376;0.402)	0.877 (0.853;0.9)	0.321 (0.308;0.334)	0.392 (0.366;0.418)	0.489 (0.475;0.502)	1.31 (1.289;1.331)	0.511 (0.489;0.533)	-1.001 (-1.04;-0.962)	0.584 (0.565;0.603)	-0.135 (-0.165;-0.105)	0.598 (0.574;0.622)	-0.596 (-0.631;-0.562)
UKPDS 82 C-HF	0.433 (0.411;0.455)	0.258 (0.232;0.284)	0.369 (0.346;0.391)	-0.198 (-0.227;-0.168)	0.549 (0.525;0.572)	0.663 (0.64;0.687)	0.543 (0.508;0.578)	-1.529 (-1.573;-1.485)	0.665 (0.631;0.699)	-0.698 (-0.732;-0.664)	0.669 (0.625;0.712)	-1.139 (-1.179;-1.1)
UKPDS 82 CHD	0.419 (0.402;0.436)	-0.383 (-0.399;-0.367)	0.338 (0.319;0.358)	-0.837 (-0.854;-0.819)	0.51 (0.494;0.527)	0.019 (0.004;0.033)	0.556 (0.528;0.585)	-2.16 (-2.188;-2.132)	0.575 (0.554;0.596)	-1.334 (-1.355;-1.313)	0.602 (0.579;0.625)	-1.773 (-1.797;-1.749)
SCORE CHD	0.383 (0.373;0.393)	-0.639 (-0.656;-0.622)	0.31 (0.299;0.321)	-1.144 (-1.163;-1.125)	0.461 (0.451;0.471)	-0.191 (-0.207;-0.176)	0.405 (0.387;0.423)	-2.636 (-2.665;-2.607)	0.454 (0.44;0.468)	-1.701 (-1.722;-1.679)	0.449 (0.434;0.465)	-2.195 (-2.22;-2.17)

ESM Table 15 Calibration (calibration-in-the-large (CIL) and calibration slope (CS)) after model recalibration, using the imputed datasets, predicting 10-years risk of CVD in type 2 diabetes patients.

	CVD		CHD		CVD + AF + HF		Stroke		AF		HF	
	**CIL**	**CS**	**CIL**	**CS**	**CIL**	**CS**	**CIL**	**CS**	**CIL**	**CS**	**CIL**	**CS**
ASCVD	-0.026 (-0.04;-0.012)	0.974 (0.935;1.013)	-0.013 (-0.029;0.002)	1.012 (0.959;1.066)	-0.04 (-0.053;-0.027)	0.977 (0.949;1.006)	-0.073 (-0.1;-0.047)	0.976 (0.921;1.031)	-0.046 (-0.065;-0.027)	1.048 (1.012;1.084)	-0.023 (-0.046;0)	0.975 (0.937;1.014)
CHS Advanced	-0.028 (-0.041;-0.014)	0.97 (0.946;0.995)	-0.014 (-0.03;0.001)	1.023 (0.988;1.058)	-0.042 (-0.055;-0.03)	0.976 (0.957;0.995)	-0.078 (-0.104;-0.052)	0.963 (0.927;1)	-0.051 (-0.07;-0.032)	1.036 (1.009;1.062)	-0.028 (-0.05;-0.006)	0.957 (0.928;0.986)
CHS Basic	-0.027 (-0.041;-0.014)	0.973 (0.948;0.998)	-0.014 (-0.03;0.001)	1.025 (0.989;1.06)	-0.042 (-0.054;-0.029)	0.977 (0.957;0.997)	-0.077 (-0.103;-0.051)	0.964 (0.926;1.003)	-0.05 (-0.069;-0.032)	1.034 (1.006;1.061)	-0.027 (-0.05;-0.005)	0.958 (0.928;0.988)
DARTS	-0.021 (-0.035;-0.007)	0.98 (0.91;1.051)	-0.01 (-0.026;0.006)	1.012 (0.906;1.117)	-0.034 (-0.047;-0.021)	0.979 (0.933;1.024)	-0.067 (-0.094;-0.04)	1.004 (0.895;1.112)	-0.04 (-0.059;-0.021)	1.035 (0.941;1.128)	-0.016 (-0.039;0.007)	0.992 (0.894;1.089)
Finrisk CHD	-0.023 (-0.037;-0.009)	0.993 (0.96;1.026)	-0.011 (-0.027;0.005)	1.033 (0.984;1.083)	-0.037 (-0.05;-0.024)	0.99 (0.958;1.022)	-0.07 (-0.096;-0.043)	0.977 (0.922;1.032)	-0.043 (-0.062;-0.023)	1.025 (0.972;1.079)	-0.019 (-0.042;0.004)	0.995 (0.947;1.043)
Finrisk CVD	-0.025 (-0.039;-0.011)	0.979 (0.952;1.007)	-0.012 (-0.028;0.003)	1.021 (0.979;1.064)	-0.038 (-0.051;-0.025)	0.982 (0.956;1.007)	-0.071 (-0.098;-0.045)	0.971 (0.924;1.019)	-0.044 (-0.063;-0.025)	1.026 (0.986;1.067)	-0.02 (-0.043;0.003)	0.998 (0.956;1.041)
Finrisk Stroke	-0.025 (-0.038;-0.011)	0.962 (0.937;0.987)	-0.012 (-0.028;0.003)	1.006 (0.967;1.044)	-0.039 (-0.051;-0.026)	0.97 (0.948;0.992)	-0.071 (-0.097;-0.045)	0.965 (0.922;1.008)	-0.045 (-0.064;-0.026)	1.024 (0.992;1.056)	-0.02 (-0.043;0.002)	1.002 (0.965;1.039)
Framingham 1991 CVD	-0.019 (-0.033;-0.005)	1.008 (0.957;1.059)	-0.008 (-0.024;0.008)	1.042 (0.958;1.125)	-0.031 (-0.043;-0.018)	0.974 (0.926;1.021)	-0.064 (-0.091;-0.038)	0.979 (0.868;1.091)	-0.037 (-0.056;-0.018)	0.93 (0.864;0.995)	-0.014 (-0.036;0.008)	1.022 (0.94;1.105)
Framingham 1991 fatal CHD	-0.02 (-0.034;-0.006)	1.016 (0.967;1.066)	-0.009 (-0.024;0.007)	1.07 (0.99;1.151)	-0.032 (-0.045;-0.019)	0.992 (0.95;1.035)	-0.066 (-0.092;-0.04)	0.967 (0.856;1.077)	-0.039 (-0.058;-0.02)	0.964 (0.899;1.03)	-0.015 (-0.038;0.007)	1.029 (0.948;1.111)
Framingham 1991 Stroke	-0.021 (-0.035;-0.007)	0.99 (0.947;1.034)	-0.009 (-0.025;0.007)	1.038 (0.97;1.106)	-0.034 (-0.047;-0.021)	0.953 (0.915;0.99)	-0.066 (-0.093;-0.039)	1.002 (0.939;1.066)	-0.04 (-0.059;-0.02)	0.975 (0.928;1.023)	-0.016 (-0.038;0.007)	0.991 (0.932;1.05)
Framingham 1998	-0.027 (-0.041;-0.014)	0.986 (0.954;1.018)	-0.014 (-0.03;0.001)	1.032 (0.987;1.077)	-0.042 (-0.055;-0.03)	0.975 (0.948;1.002)	-0.076 (-0.102;-0.05)	0.982 (0.945;1.019)	-0.049 (-0.068;-0.03)	1.013 (0.976;1.051)	-0.026 (-0.049;-0.003)	0.963 (0.925;1.001)
QRISK 2	-0.02 (-0.034;-0.006)	1.022 (0.966;1.079)	-0.008 (-0.024;0.007)	1.073 (0.998;1.149)	-0.034 (-0.047;-0.021)	1.011 (0.969;1.053)	-0.063 (-0.09;-0.037)	1.036 (0.913;1.159)	-0.038 (-0.057;-0.019)	1.068 (0.995;1.142)	-0.013 (-0.036;0.01)	1.066 (0.994;1.138)
QRISK 3	-0.022 (-0.036;-	1.038	-0.01 (-	1.097	-0.036 (-0.048;-	1.025	-0.065 (-0.092;-	1.052	-0.04 (-0.059;-	1.084	-0.015 (-	1.094

	0.008)	(0.979;1.098)	0.025;0.006)	(1.014;1.18)	0.023)	(0.981;1.069)	0.038)	(0.92;1.184)	0.021)	(1.003;1.164)	0.038;0.008)	(1.015;1.173)
RECODE	-0.023 (-0.037;-0.009)	0.999 (0.938;1.059)	-0.011 (-0.027;0.005)	1.032 (0.937;1.127)	-0.035 (-0.048;-0.022)	1.018 (0.982;1.053)	-0.07 (-0.096;-0.043)	0.947 (0.813;1.08)	-0.042 (-0.061;-0.023)	1.044 (0.957;1.13)	-0.018 (-0.041;0.004)	0.99 (0.883;1.096)
Reynolds Risk	-0.023 (-0.037;-0.008)	0.995 (0.96;1.03)	-0.011 (-0.027;0.005)	1.034 (0.98;1.088)	-0.036 (-0.049;-0.022)	0.994 (0.96;1.027)	-0.069 (-0.096;-0.042)	0.976 (0.911;1.041)	-0.042 (-0.061;-0.022)	1.034 (0.967;1.101)	-0.018 (-0.041;0.005)	1.008 (0.949;1.067)
SCORE CHD	-0.023 (-0.037;-0.009)	0.986 (0.955;1.016)	-0.011 (-0.027;0.004)	1.011 (0.972;1.05)	-0.037 (-0.05;-0.024)	0.986 (0.96;1.012)	-0.07 (-0.096;-0.044)	0.995 (0.947;1.042)	-0.043 (-0.062;-0.024)	1.04 (1;1.08)	-0.019 (-0.041;0.003)	0.975 (0.934;1.016)
SCORE CVD	-0.024 (-0.038;-0.011)	0.98 (0.95;1.011)	-0.012 (-0.028;0.004)	1.014 (0.973;1.054)	-0.038 (-0.051;-0.025)	0.981 (0.955;1.007)	-0.072 (-0.098;-0.045)	0.988 (0.947;1.03)	-0.044 (-0.063;-0.025)	1.042 (1.004;1.08)	-0.021 (-0.043;0.001)	0.969 (0.929;1.01)
UKPDS 56	-0.02 (-0.033;-0.006)	1.007 (0.962;1.052)	-0.008 (-0.024;0.007)	1.031 (0.968;1.094)	-0.032 (-0.045;-0.02)	1.018 (0.98;1.056)	-0.064 (-0.09;-0.038)	0.987 (0.885;1.089)	-0.038 (-0.056;-0.019)	1.057 (0.966;1.148)	-0.014 (-0.036;0.008)	1.064 (0.977;1.151)
UKPDS 68 C-HF	-0.024 (-0.037;-0.01)	0.979 (0.941;1.018)	-0.012 (-0.027;0.004)	1.037 (0.986;1.088)	-0.038 (-0.051;-0.025)	0.974 (0.946;1.002)	-0.071 (-0.097;-0.045)	0.976 (0.922;1.029)	-0.045 (-0.064;-0.026)	1.026 (0.984;1.068)	-0.022 (-0.044;0.001)	0.943 (0.9;0.986)
UKPDS 68 Stroke	-0.024 (-0.037;-0.01)	0.979 (0.941;1.018)	-0.012 (-0.027;0.004)	1.037 (0.986;1.088)	-0.038 (-0.051;-0.025)	0.974 (0.946;1.002)	-0.071 (-0.097;-0.045)	0.976 (0.922;1.029)	-0.045 (-0.064;-0.026)	1.026 (0.984;1.068)	-0.022 (-0.044;0.001)	0.943 (0.9;0.986)
UKPDS 82 C-HF	-0.022 (-0.036;-0.008)	0.988 (0.952;1.023)	-0.01 (-0.026;0.006)	1.051 (0.994;1.107)	-0.035 (-0.048;-0.022)	0.972 (0.943;1.002)	-0.067 (-0.094;-0.041)	0.98 (0.918;1.041)	-0.042 (-0.061;-0.022)	1.011 (0.967;1.055)	-0.017 (-0.04;0.006)	0.951 (0.901;1.002)
UKPDS 82 CHD	-0.025 (-0.038;-0.011)	0.992 (0.947;1.037)	-0.012 (-0.028;0.003)	1.073 (1.011;1.135)	-0.038 (-0.05;-0.025)	0.978 (0.945;1.012)	-0.075 (-0.101;-0.048)	0.941 (0.859;1.022)	-0.046 (-0.065;-0.028)	1 (0.946;1.055)	-0.024 (-0.047;-0.002)	0.958 (0.909;1.008)

ESM Table 16 A net reclassification index (NRI) table comparing the predicted CVD risk distributions of QRISK2 and SCORE CVD among type 2 diabetes patients with and without a CVD event during 10 years of follow-up.

QRISK2	SCORE CVD<br>Low risk [0.0, 0.1)	Intermediate risk [0.1, 0.2)	High risk [0.2, 1.0]	**Total**
**In participants without CVD**				
Low risk	20,030	2,815	2	22,847
Intermediate risk	9,120	45,915	11,107	57,934
High risk	367	5,614	30,806	36,787
**Total**	21,309	54,344	41,915	117,568
**In participants with CVD**				
Low risk	1,023	195	0	1,218
Intermediate risk	874	37,471	2,869	41,214
High risk	48	1,340	12,164	13,552
**Total**	1,945	39,006	15,033	55,984

**NRI estimates**	Pr(Up|Event): 0.118 (0.114; 0.123)
NRI: 0.040 (0.034; 0.046)	Pr(Down|Event): 0.087 (0.084; 0.090)
Event NRI: 0.031 (0.025; 0.036)	Pr(Down|Non-event): 0.120 (0.118; 0.122)
Non-event NRI: 0.009 (0.006; 0.012)	Pr(Up|Non-event): 0.111 (0.109; 0.112)

N.b. Calculations are based on the test data after recalibrating the models using an independent training dataset. Various NRI estimates are provided, including the probabilities of an increased (Up) or decreased (Down) predicted risk conditional on event status. CVD: cardiovascular disease; NRI: net reclassification index, Pr: probability.

ESM Table 17 Discrimination (c-statistic) and calibration (calibration-in-the-large (CIL) and calibration slope (CS)) of the risk scores against the CVD and CVD + AF + HF outcomes for the imputed dataset (only including type 2 diabetes patients without pre-existing CVD at baseline) stratified by age subgroups.

Risk Score Name	Age-group (years)	**CVD**			**CVD + AF + HF**		
		**C-stat**	**CS**	**CIL**	**C-stat**	**CS**	**CIL**
ASCVD	[18, 50)	0.613 (0.601;0.625)	0.305 (0.262;0.348)	-0.015 (-0.065;0.036)	0.601 (0.59;0.611)	0.273 (0.235;0.311)	0.237 (0.189;0.285)
	[50, 65)	0.594 (0.587;0.602)	0.413 (0.381;0.445)	-0.212 (-0.241;-0.184)	0.591 (0.584;0.598)	0.398 (0.368;0.428)	0.089 (0.062;0.116)
	[65, 75)	0.579 (0.572;0.587)	0.46 (0.415;0.506)	-0.59 (-0.619;-0.56)	0.576 (0.569;0.583)	0.442 (0.4;0.484)	-0.162 (-0.189;-0.135)
	[75, 105]	0.541 (0.533;0.55)	0.078 (0.053;0.103)	-1.555 (-1.588;-1.522)	0.552 (0.544;0.56)	0.105 (0.08;0.13)	-0.883 (-0.914;-0.853)
	[18, 50)	0.605 (0.595;0.616)	1.022 (0.915;1.13)	-0.038 (-0.076;0)	0.598 (0.589;0.607)	0.94 (0.843;1.037)	0.189 (0.154;0.223)
	[50, 65)	0.561 (0.555;0.568)	0.669 (0.597;0.741)	-0.289 (-0.312;-0.266)	0.571 (0.565;0.577)	0.773 (0.706;0.839)	-0.008 (-0.029;0.014)
	[65, 75)	0.553 (0.545;0.56)	0.681 (0.58;0.782)	-0.569 (-0.595;-0.543)	0.56 (0.553;0.567)	0.78 (0.688;0.872)	-0.163 (-0.187;-0.139)
CHS Advanced	[75, 105]	0.531 (0.522;0.539)	0.168 (0.105;0.23)	-1.032 (-1.062;-1.002)	0.546 (0.538;0.554)	0.282 (0.223;0.341)	-0.446 (-0.474;-0.418)
	[18, 50)	0.604 (0.594;0.614)	1.121 (1.001;1.24)	-0.342 (-0.38;-0.304)	0.596 (0.587;0.606)	1.031 (0.921;1.14)	-0.116 (-0.151;-0.082)
	[50, 65)	0.561 (0.555;0.568)	0.716 (0.638;0.795)	-0.458 (-0.481;-0.435)	0.57 (0.564;0.576)	0.819 (0.747;0.891)	-0.177 (-0.199;-0.156)
	[65, 75)	0.55 (0.542;0.558)	0.672 (0.565;0.779)	-0.622 (-0.649;-0.596)	0.556 (0.549;0.563)	0.762 (0.664;0.859)	-0.217 (-0.241;-0.193)
CHS Basic	[75, 105]	0.531 (0.522;0.539)	0.207 (0.134;0.28)	-0.927 (-0.957;-0.897)	0.544 (0.537;0.552)	0.337 (0.268;0.406)	-0.348 (-0.376;-0.321)
	[18, 50)	0.622 (0.61;0.634)	0.513 (0.455;0.57)	-0.912 (-0.963;-0.861)	0.611 (0.6;0.622)	0.478 (0.424;0.532)	-0.675 (-0.723;-0.627)
	[50, 65)	0.567 (0.558;0.576)	0.276 (0.19;0.363)	-0.984 (-1.015;-0.953)	0.563 (0.555;0.57)	0.268 (0.192;0.345)	-0.686 (-0.715;-0.657)
	[65, 75)	0.549 (0.541;0.557)	0.238 (0.17;0.306)	-1.016 (-1.048;-0.984)	0.541 (0.534;0.549)	0.207 (0.151;0.263)	-0.582 (-0.611;-0.554)
DARTS	[75, 105]	0.53 (0.519;0.54)	0.118 (0.074;0.163)	-1.26 (-1.298;-1.222)	0.53 (0.522;0.539)	0.117 (0.078;0.156)	-0.633 (-0.668;-0.598)
	[18, 50)	0.631 (0.62;0.643)	0.475 (0.43;0.519)	1.062 (1.01;1.114)	0.619 (0.608;0.629)	0.427 (0.386;0.468)	1.304 (1.255;1.353)
Finrisk CHD	[50, 65)	0.587 (0.579;0.594)	0.374 (0.338;0.409)	0.61 (0.579;0.642)	0.584 (0.577;0.591)	0.363 (0.328;0.398)	0.913 (0.883;0.943)

	[65, 75)	0.569 (0.56;0.578)	0.352 (0.306;0.397)	0.328 (0.293;0.363)	0.562 (0.555;0.57)	0.319 (0.276;0.363)	0.764 (0.731;0.796)
	[75, 105]	0.542 (0.532;0.552)	0.19 (0.143;0.238)	-0.085 (-0.118;-0.052)	0.545 (0.536;0.554)	0.198 (0.156;0.24)	0.54 (0.508;0.572)
	[18, 50)	0.628 (0.617;0.64)	0.621 (0.562;0.68)	1.219 (1.173;1.265)	0.619 (0.608;0.629)	0.57 (0.514;0.625)	1.454 (1.41;1.497)
	[50, 65)	0.589 (0.581;0.596)	0.482 (0.442;0.522)	0.691 (0.663;0.719)	0.589 (0.582;0.596)	0.484 (0.446;0.521)	0.985 (0.959;1.011)
	[65, 75)	0.578 (0.57;0.586)	0.45 (0.4;0.499)	0.308 (0.277;0.339)	0.572 (0.565;0.579)	0.414 (0.367;0.46)	0.737 (0.708;0.766)
Finrisk CVD	[75, 105]	0.549 (0.54;0.559)	0.239 (0.192;0.285)	-0.079 (-0.11;-0.047)	0.553 (0.544;0.562)	0.251 (0.208;0.294)	0.541 (0.511;0.571)
	[18, 50)	0.587 (0.576;0.598)	0.449 (0.392;0.506)	1.427 (1.386;1.468)	0.584 (0.574;0.594)	0.426 (0.373;0.479)	1.659 (1.621;1.697)
	[50, 65)	0.582 (0.575;0.589)	0.513 (0.468;0.557)	0.785 (0.76;0.811)	0.586 (0.58;0.593)	0.544 (0.503;0.585)	1.075 (1.052;1.099)
	[65, 75)	0.575 (0.568;0.583)	0.426 (0.382;0.47)	0.296 (0.267;0.325)	0.57 (0.564;0.577)	0.4 (0.36;0.44)	0.727 (0.7;0.753)
Finrisk Stroke	[75, 105]	0.547 (0.538;0.555)	0.185 (0.15;0.22)	-0.078 (-0.109;-0.046)	0.549 (0.541;0.558)	0.198 (0.165;0.232)	0.576 (0.547;0.606)
	[18, 50)	0.618 (0.607;0.63)	0.402 (0.361;0.442)	-2.123 (-2.176;-2.07)	0.608 (0.598;0.619)	0.364 (0.327;0.4)	-1.879 (-1.93;-1.827)
	[50, 65)	0.583 (0.575;0.592)	0.348 (0.313;0.383)	-2.077 (-2.109;-2.046)	0.579 (0.571;0.587)	0.328 (0.296;0.359)	-1.77 (-1.8;-1.74)
	[65, 75)	0.565 (0.556;0.573)	0.312 (0.273;0.351)	-1.798 (-1.832;-1.763)	0.558 (0.55;0.566)	0.28 (0.243;0.316)	-1.353 (-1.385;-1.321)
Framingham 1991 CVD	[75, 105]	0.541 (0.531;0.55)	0.204 (0.156;0.253)	-1.489 (-1.522;-1.456)	0.541 (0.533;0.55)	0.207 (0.164;0.25)	-0.874 (-0.904;-0.844)
	[18, 50)	0.625 (0.613;0.637)	0.313 (0.28;0.346)	0.5 (0.444;0.557)	0.615 (0.604;0.625)	0.28 (0.251;0.31)	0.751 (0.696;0.805)
	[50, 65)	0.58 (0.571;0.589)	0.33 (0.293;0.367)	0.258 (0.227;0.289)	0.576 (0.568;0.585)	0.313 (0.28;0.347)	0.56 (0.531;0.59)
	[65, 75)	0.56 (0.551;0.569)	0.33 (0.283;0.378)	0.391 (0.359;0.423)	0.553 (0.545;0.562)	0.292 (0.247;0.336)	0.821 (0.791;0.852)
Framingham 1991 fatal CHD	[75, 105]	0.536 (0.526;0.546)	0.218 (0.155;0.282)	0.566 (0.535;0.598)	0.537 (0.528;0.545)	0.222 (0.168;0.276)	1.162 (1.133;1.192)
	[18, 50)	0.619 (0.608;0.631)	0.369 (0.333;0.405)	0.369 (0.311;0.427)	0.612 (0.602;0.622)	0.344 (0.311;0.377)	0.621 (0.566;0.676)
	[50, 65)	0.574 (0.565;0.583)	0.249 (0.221;0.278)	-0.143 (-0.179;-0.107)	0.575 (0.567;0.582)	0.248 (0.224;0.273)	0.187 (0.153;0.222)
	[65, 75)	0.554 (0.546;0.562)	0.182 (0.155;0.208)	-0.273 (-0.315;-0.232)	0.553 (0.546;0.561)	0.18 (0.156;0.204)	0.214 (0.177;0.252)
Framingham 1991 Stroke	[75, 105]	0.536 (0.527;0.545)	0.1 (0.073;0.126)	-0.324 (-0.36;-0.288)	0.541 (0.533;0.549)	0.111 (0.086;0.136)	0.358 (0.325;0.39)
Framingham 1998	[18, 50)	0.626 (0.615;0.637)	0.979 (0.891;1.068)	0.019 (-0.019;0.058)	0.62 (0.61;0.63)	0.923 (0.84;1.006)	0.247 (0.212;0.283)

	[50, 65)	0.575 (0.567;0.583)	0.65 (0.584;0.716)	-0.054 (-0.08;-0.028)	0.579 (0.572;0.587)	0.687 (0.625;0.748)	0.23 (0.206;0.254)
	[65, 75)	0.56 (0.552;0.569)	0.572 (0.488;0.656)	-0.144 (-0.172;-0.117)	0.562 (0.554;0.57)	0.576 (0.5;0.652)	0.266 (0.241;0.292)
	[75, 105]	0.538 (0.53;0.547)	0.271 (0.203;0.34)	-0.368 (-0.397;-0.338)	0.549 (0.541;0.557)	0.347 (0.284;0.41)	0.216 (0.188;0.244)
	[18, 50)	0.636 (0.625;0.648)	0.485 (0.441;0.53)	-0.492 (-0.537;-0.448)	0.627 (0.616;0.638)	0.457 (0.415;0.499)	-0.25 (-0.291;-0.208)
	[50, 65)	0.593 (0.585;0.601)	0.448 (0.388;0.508)	-0.597 (-0.623;-0.571)	0.591 (0.583;0.598)	0.447 (0.393;0.5)	-0.301 (-0.326;-0.276)
	[65, 75)	0.564 (0.557;0.572)	0.398 (0.339;0.458)	-0.61 (-0.642;-0.579)	0.561 (0.554;0.569)	0.385 (0.33;0.44)	-0.188 (-0.217;-0.159)
QRISK2	[75, 105]	0.526 (0.516;0.536)	0.13 (0.07;0.19)	-0.808 (-0.839;-0.777)	0.533 (0.524;0.542)	0.169 (0.111;0.226)	-0.21 (-0.24;-0.181)
	[18, 50)	0.636 (0.625;0.647)	0.503 (0.457;0.548)	-0.343 (-0.387;-0.298)	0.627 (0.616;0.638)	0.475 (0.432;0.518)	-0.101 (-0.143;-0.059)
	[50, 65)	0.591 (0.582;0.599)	0.449 (0.387;0.511)	-0.424 (-0.451;-0.398)	0.59 (0.583;0.598)	0.454 (0.399;0.51)	-0.13 (-0.154;-0.105)
	[65, 75)	0.567 (0.56;0.575)	0.432 (0.369;0.495)	-0.397 (-0.428;-0.366)	0.564 (0.556;0.571)	0.417 (0.358;0.476)	0.024 (-0.004;0.053)
QRISK3	[75, 105]	0.537 (0.527;0.547)	0.205 (0.14;0.271)	-0.541 (-0.572;-0.51)	0.541 (0.532;0.55)	0.225 (0.163;0.287)	0.052 (0.023;0.081)
	[18, 50)	0.587 (0.575;0.599)	0.586 (0.509;0.664)	-0.469 (-0.508;-0.429)	0.579 (0.568;0.59)	0.546 (0.473;0.618)	-0.239 (-0.275;-0.203)
	[50, 65)	0.558 (0.549;0.567)	0.394 (0.332;0.456)	-0.254 (-0.278;-0.23)	0.555 (0.547;0.563)	0.381 (0.323;0.439)	0.034 (0.012;0.055)
	[65, 75)	0.556 (0.548;0.565)	0.371 (0.31;0.432)	-0.094 (-0.122;-0.065)	0.551 (0.544;0.559)	0.339 (0.281;0.396)	0.326 (0.3;0.352)
RECODE	[75, 105]	0.541 (0.532;0.55)	0.217 (0.151;0.282)	-0.044 (-0.074;-0.013)	0.545 (0.537;0.553)	0.257 (0.198;0.315)	0.549 (0.52;0.578)
	[18, 50)	0.63 (0.618;0.641)	0.505 (0.456;0.554)	1.084 (1.035;1.134)	0.619 (0.609;0.63)	0.457 (0.412;0.503)	1.322 (1.276;1.369)
	[50, 65)	0.586 (0.578;0.594)	0.369 (0.337;0.402)	0.532 (0.501;0.562)	0.584 (0.577;0.591)	0.358 (0.329;0.388)	0.834 (0.805;0.862)
	[65, 75)	0.57 (0.562;0.579)	0.307 (0.268;0.347)	0.304 (0.269;0.34)	0.562 (0.555;0.57)	0.273 (0.235;0.311)	0.749 (0.717;0.782)
Reynolds Risk	[75, 105]	0.547 (0.537;0.556)	0.163 (0.127;0.198)	0.02 (-0.012;0.053)	0.548 (0.539;0.557)	0.165 (0.133;0.198)	0.67 (0.639;0.702)
	[18, 50)	0.633 (0.623;0.644)	0.306 (0.278;0.333)	3.331 (3.277;3.385)	0.619 (0.61;0.629)	0.268 (0.244;0.292)	3.584 (3.533;3.635)
	[50, 65)	0.593 (0.586;0.6)	0.363 (0.335;0.392)	2.452 (2.423;2.481)	0.591 (0.584;0.597)	0.353 (0.327;0.379)	2.76 (2.733;2.787)
	[65, 75)	0.575 (0.566;0.583)	0.383 (0.34;0.427)	2.11 (2.079;2.141)	0.567 (0.56;0.575)	0.348 (0.308;0.387)	2.546 (2.518;2.575)
SCORE CHD	[75, 105]	0.546 (0.537;0.556)	0.253 (0.203;0.303)	1.789 (1.755;1.822)	0.549 (0.541;0.557)	0.261 (0.217;0.305)	2.399 (2.368;2.43)

	[18, 50)	0.635 (0.625;0.646)	0.332 (0.302;0.362)	3.001 (2.95;3.053)	0.622 (0.613;0.632)	0.292 (0.266;0.319)	3.251 (3.201;3.3)
	[50, 65)	0.594 (0.587;0.601)	0.404 (0.373;0.435)	1.968 (1.94;1.996)	0.594 (0.588;0.6)	0.403 (0.374;0.431)	2.272 (2.245;2.298)
	[65, 75)	0.576 (0.568;0.584)	0.426 (0.379;0.472)	1.477 (1.447;1.508)	0.571 (0.564;0.579)	0.403 (0.361;0.446)	1.909 (1.88;1.937)
SCORE CVD	[75, 105]	0.546 (0.537;0.555)	0.232 (0.185;0.278)	1 (0.968;1.033)	0.552 (0.544;0.56)	0.252 (0.209;0.294)	1.615 (1.585;1.646)
	[18, 50)	0.605 (0.593;0.617)	0.411 (0.364;0.458)	-0.38 (-0.426;-0.335)	0.592 (0.58;0.604)	0.361 (0.318;0.404)	-0.14 (-0.183;-0.097)
	[50, 65)	0.575 (0.567;0.584)	0.294 (0.262;0.327)	-0.403 (-0.434;-0.372)	0.569 (0.561;0.577)	0.273 (0.242;0.304)	-0.093 (-0.122;-0.063)
	[65, 75)	0.562 (0.554;0.57)	0.236 (0.204;0.269)	-0.344 (-0.383;-0.305)	0.551 (0.544;0.559)	0.2 (0.169;0.23)	0.117 (0.082;0.152)
UKPDS 56	[75, 105]	0.543 (0.533;0.553)	0.15 (0.115;0.184)	-0.4 (-0.433;-0.366)	0.542 (0.534;0.551)	0.146 (0.116;0.177)	0.264 (0.233;0.296)
	[18, 50)	0.574 (0.563;0.586)	0.31 (0.258;0.362)	1.739 (1.69;1.788)	0.579 (0.569;0.589)	0.335 (0.29;0.38)	1.977 (1.931;2.023)
	[50, 65)	0.532 (0.524;0.54)	0.163 (0.126;0.2)	1.201 (1.167;1.235)	0.549 (0.543;0.556)	0.25 (0.218;0.282)	1.5 (1.468;1.532)
	[65, 75)	0.527 (0.519;0.536)	0.148 (0.094;0.202)	0.779 (0.735;0.823)	0.54 (0.532;0.548)	0.226 (0.169;0.283)	1.211 (1.171;1.251)
UKPDS 68 C-HF	[75, 105]	0.522 (0.513;0.531)	0.091 (0.049;0.134)	0.216 (0.169;0.263)	0.54 (0.531;0.549)	0.165 (0.122;0.207)	0.843 (0.8;0.886)
	[18, 50)	0.574 (0.563;0.586)	0.31 (0.258;0.362)	1.739 (1.69;1.788)	0.579 (0.569;0.589)	0.335 (0.29;0.38)	1.977 (1.931;2.023)
	[50, 65)	0.532 (0.524;0.54)	0.163 (0.126;0.2)	1.201 (1.167;1.235)	0.549 (0.543;0.556)	0.25 (0.218;0.282)	1.5 (1.468;1.532)
	[65, 75)	0.527 (0.519;0.536)	0.148 (0.094;0.202)	0.779 (0.735;0.823)	0.54 (0.532;0.548)	0.226 (0.169;0.283)	1.211 (1.171;1.251)
UKPDS 68 Stroke	[75, 105]	0.522 (0.513;0.531)	0.091 (0.049;0.134)	0.216 (0.169;0.263)	0.54 (0.531;0.549)	0.165 (0.122;0.207)	0.843 (0.8;0.886)
	[18, 50)	0.573 (0.56;0.586)	0.307 (0.25;0.363)	0.606 (0.561;0.652)	0.578 (0.567;0.589)	0.335 (0.284;0.386)	0.844 (0.802;0.887)
	[50, 65)	0.525 (0.514;0.535)	0.133 (0.076;0.189)	0.403 (0.366;0.439)	0.542 (0.533;0.551)	0.218 (0.172;0.265)	0.7 (0.666;0.734)
	[65, 75)	0.519 (0.509;0.529)	0.11 (0.046;0.174)	0.226 (0.18;0.271)	0.533 (0.525;0.541)	0.184 (0.119;0.249)	0.655 (0.614;0.695)
UKPDS 82 C-HF	[75, 105]	0.51 (0.5;0.521)	0.049 (-0.01;0.107)	-0.077 (-0.127;-0.027)	0.529 (0.518;0.539)	0.13 (0.071;0.189)	0.534 (0.489;0.58)

	[18, 50)	0.554 (0.542;0.567)	0.294 (0.228;0.36)	-0.222 (-0.263;-0.181)	0.554 (0.542;0.565)	0.29 (0.23;0.35)	0.01 (-0.028;0.049)
	[50, 65)	0.514 (0.504;0.524)	0.099 (0.048;0.15)	-0.259 (-0.284;-0.234)	0.519 (0.509;0.529)	0.123 (0.072;0.174)	0.036 (0.013;0.059)
	[65, 75)	0.508 (0.496;0.519)	0.068 (0.016;0.121)	-0.376 (-0.407;-0.345)	0.512 (0.502;0.522)	0.085 (0.037;0.133)	0.059 (0.03;0.089)
UKPDS 82 CHD	[75, 105]	0.507 (0.497;0.516)	0.038 (-0.002;0.078)	-0.663 (-0.697;-0.629)	0.514 (0.506;0.522)	0.066 (0.03;0.102)	-0.032 (-0.064;0.001)

ESM Table 18 Discrimination (c-statistic) and calibration (calibration-in-the-large (CIL) and calibration slope (CS)) of the risk scores against the CVD and CVD + AF + HF outcomes for the imputed dataset (only including type 2 diabetes patients without pre-existing CVD at baseline) stratified by sex.

Risk Score Name	Sex (male vs female)	CVD			CVD + AF + HF		
		C-stat	CS	CIL	C-stat	CS	CIL
ASCVD	male	0.658 (0.653;0.663)	0.477 (0.46;0.494)	-0.571 (-0.593;-0.549)	0.681 (0.676;0.685)	0.559 (0.542;0.575)	-0.2 (-0.221;-0.179)
	female	0.678 (0.673;0.683)	0.329 (0.317;0.342)	-0.73 (-0.755;-0.706)	0.701 (0.696;0.706)	0.405 (0.392;0.418)	-0.166 (-0.188;-0.144)
CHD Advanced	male	0.651 (0.646;0.656)	0.576 (0.556;0.597)	-0.39 (-0.409;-0.371)	0.679 (0.675;0.684)	0.714 (0.694;0.733)	-0.038 (-0.055;-0.02)
	female	0.678 (0.673;0.683)	0.645 (0.622;0.668)	-0.694 (-0.716;-0.672)	0.706 (0.701;0.71)	0.774 (0.753;0.795)	-0.239 (-0.259;-0.219)
CHS Basic	male	0.649 (0.644;0.654)	0.666 (0.642;0.689)	-0.497 (-0.516;-0.478)	0.677 (0.673;0.681)	0.824 (0.801;0.846)	-0.157 (-0.174;-0.139)
	female	0.677 (0.671;0.682)	0.763 (0.736;0.789)	-0.737 (-0.758;-0.715)	0.705 (0.7;0.709)	0.914 (0.889;0.939)	-0.297 (-0.317;-0.278)
DARTS	male	0.643 (0.638;0.648)	0.482 (0.448;0.515)	-0.942 (-0.966;-0.918)	0.659 (0.654;0.664)	0.563 (0.533;0.592)	-0.588 (-0.61;-0.566)
	female	0.665 (0.659;0.67)	0.536 (0.51;0.563)	-1.179 (-1.203;-1.156)	0.682 (0.677;0.687)	0.622 (0.597;0.647)	-0.718 (-0.739;-0.696)
Finrisk CHD	male	0.654 (0.649;0.659)	0.497 (0.478;0.516)	0.452 (0.427;0.478)	0.674 (0.67;0.679)	0.573 (0.555;0.59)	0.82 (0.796;0.843)
	female	0.678 (0.672;0.683)	0.462 (0.445;0.479)	0.338 (0.313;0.363)	0.698 (0.693;0.703)	0.526 (0.51;0.542)	0.839 (0.817;0.862)
Finrisk CVD	male	0.661 (0.656;0.666)	0.468 (0.452;0.484)	0.422 (0.399;0.445)	0.685 (0.681;0.689)	0.551 (0.536;0.566)	0.803 (0.782;0.825)
	female	0.679 (0.674;0.685)	0.582 (0.561;0.603)	0.464 (0.44;0.487)	0.701 (0.696;0.705)	0.667 (0.648;0.686)	0.932 (0.912;0.953)
Finrisk Stroke	male	0.661 (0.656;0.665)	0.388 (0.375;0.4)	0.405 (0.383;0.426)	0.687 (0.682;0.691)	0.464 (0.451;0.476)	0.814 (0.794;0.834)
	female	0.68 (0.674;0.685)	0.736 (0.709;0.762)	0.596 (0.574;0.618)	0.702 (0.698;0.707)	0.851 (0.826;0.875)	1.039 (1.019;1.059)
Framingham 1991 CVD	male	0.626 (0.621;0.632)	0.481 (0.459;0.503)	-1.909 (-1.933;-1.885)	0.635 (0.63;0.64)	0.515 (0.496;0.535)	-1.559 (-1.582;-1.536)
	female	0.632 (0.626;0.638)	0.516 (0.49;0.543)	-1.859 (-1.883;-1.835)	0.639 (0.633;0.644)	0.544 (0.518;0.57)	-1.413 (-1.434;-1.391)
Framingham 1991 fatal CHD	male	0.636 (0.631;0.642)	0.472 (0.45;0.494)	0.453 (0.429;0.477)	0.647 (0.642;0.652)	0.509 (0.489;0.529)	0.801 (0.778;0.824)
	female	0.653 (0.647;0.659)	0.516 (0.492;0.54)	0.315 (0.292;0.338)	0.664 (0.658;0.67)	0.554 (0.531;0.576)	0.763 (0.742;0.784)
Framingham 1991 Stroke	male	0.642 (0.637;0.647)	0.359 (0.344;0.374)	0.041 (0.016;0.066)	0.658 (0.653;0.662)	0.406 (0.392;0.42)	0.437 (0.413;0.46)
	female	0.664 (0.659;0.67)	0.386 (0.369;0.403)	-0.404 (-0.432;-0.376)	0.679 (0.674;0.684)	0.439 (0.422;0.455)	0.106 (0.081;0.131)
Framingham 1998	male	0.66 (0.655;0.664)	0.759 (0.734;0.785)	0.048 (0.029;0.068)	0.683 (0.679;0.687)	0.896 (0.872;0.92)	0.385 (0.367;0.403)
	female	0.683 (0.678;0.689)	0.801 (0.774;0.829)	-0.372 (-0.394;-0.35)	0.707 (0.702;0.712)	0.933 (0.907;0.959)	0.066 (0.047;0.086)

QRISK2	male	0.653 (0.648;0.658)	0.568 (0.545;0.591)	-0.567 (-0.589;-0.545)	0.671 (0.666;0.675)	0.652 (0.63;0.673)	-0.22 (-0.241;-0.2)
	female	0.678 (0.672;0.683)	0.631 (0.605;0.656)	-0.728 (-0.751;-0.706)	0.699 (0.694;0.704)	0.733 (0.709;0.756)	-0.275 (-0.295;-0.255)
QRISK3	male	0.653 (0.648;0.658)	0.589 (0.564;0.614)	-0.36 (-0.382;-0.337)	0.67 (0.666;0.675)	0.674 (0.651;0.697)	-0.015 (-0.036;0.006)
	female	0.679 (0.674;0.685)	0.684 (0.656;0.713)	-0.529 (-0.551;-0.507)	0.7 (0.695;0.705)	0.788 (0.762;0.814)	-0.083 (-0.103;-0.063)
RECODE	male	0.625 (0.619;0.631)	0.655 (0.621;0.689)	-0.2 (-0.219;-0.18)	0.642 (0.637;0.648)	0.779 (0.746;0.813)	0.13 (0.112;0.147)
	female	0.655 (0.649;0.662)	0.821 (0.782;0.86)	-0.197 (-0.218;-0.175)	0.674 (0.668;0.679)	0.949 (0.916;0.982)	0.228 (0.209;0.248)
Reynolds Risk	male	0.657 (0.652;0.662)	0.505 (0.487;0.524)	0.416 (0.393;0.44)	0.678 (0.673;0.682)	0.58 (0.563;0.598)	0.782 (0.76;0.804)
	female	0.668 (0.662;0.673)	0.426 (0.409;0.443)	0.386 (0.362;0.411)	0.685 (0.68;0.69)	0.484 (0.469;0.499)	0.891 (0.869;0.913)
SCORE CHD	male	0.658 (0.653;0.663)	0.472 (0.455;0.49)	2.176 (2.154;2.198)	0.679 (0.675;0.684)	0.541 (0.525;0.557)	2.545 (2.524;2.566)
	female	0.681 (0.676;0.686)	0.386 (0.372;0.4)	2.384 (2.36;2.408)	0.704 (0.7;0.709)	0.442 (0.429;0.455)	2.898 (2.877;2.92)
SCORE CVD	male	0.66 (0.655;0.665)	0.44 (0.424;0.455)	1.684 (1.662;1.706)	0.683 (0.679;0.687)	0.51 (0.495;0.525)	2.063 (2.043;2.084)
	female	0.683 (0.678;0.688)	0.374 (0.36;0.387)	1.607 (1.583;1.63)	0.707 (0.703;0.712)	0.43 (0.418;0.443)	2.131 (2.11;2.153)
UKPDS 56	male	0.63 (0.624;0.635)	0.432 (0.412;0.452)	-0.588 (-0.617;-0.559)	0.643 (0.638;0.648)	0.49 (0.471;0.508)	-0.226 (-0.252;-0.199)
	female	0.652 (0.646;0.658)	0.527 (0.502;0.552)	-0.08 (-0.103;-0.057)	0.668 (0.662;0.673)	0.596 (0.575;0.617)	0.38 (0.359;0.401)
UKPDS 68 C-HF	male	0.643 (0.638;0.648)	0.419 (0.403;0.436)	1.238 (1.208;1.268)	0.672 (0.667;0.677)	0.518 (0.501;0.535)	1.617 (1.588;1.646)
	female	0.666 (0.66;0.671)	0.433 (0.414;0.452)	0.477 (0.436;0.517)	0.695 (0.69;0.7)	0.529 (0.507;0.551)	0.975 (0.94;1.01)
UKPDS 68 Stroke	male	0.643 (0.638;0.648)	0.419 (0.403;0.436)	1.238 (1.208;1.268)	0.672 (0.667;0.677)	0.518 (0.501;0.535)	1.617 (1.588;1.646)
	female	0.666 (0.66;0.671)	0.433 (0.414;0.452)	0.477 (0.436;0.517)	0.695 (0.69;0.7)	0.529 (0.507;0.551)	0.975 (0.94;1.01)
UKPDS 82 C-HF	male	0.632 (0.626;0.638)	0.488 (0.465;0.51)	0.529 (0.499;0.558)	0.661 (0.656;0.667)	0.614 (0.591;0.637)	0.881 (0.853;0.908)
	female	0.649 (0.641;0.656)	0.444 (0.413;0.475)	-0.056 (-0.101;-0.01)	0.677 (0.671;0.683)	0.547 (0.513;0.58)	0.416 (0.376;0.456)
UKPDS 82 CHD	male	0.636 (0.63;0.642)	0.655 (0.626;0.684)	0.177 (0.158;0.196)	0.655 (0.65;0.661)	0.765 (0.736;0.794)	0.511 (0.494;0.529)
	female	0.663 (0.655;0.671)	0.535 (0.506;0.564)	-0.953 (-0.977;-0.929)	0.683 (0.676;0.69)	0.615 (0.589;0.642)	-0.496 (-0.518;-0.474)

ESM Table 19 Discrimination (c-statistic) and calibration (calibration-in-the-large (CIL) and calibration slope (CS)) of the risk scores against the CVD and CVD + AF + HF outcomes for the imputed dataset (only including type 2 diabetes patients without pre-existing CVD at baseline) stratified by statin usage at baseline.

Risk Score Name	Statins usage at baseline (statin naïve vs statin users)	CVD			CVD + AF + HF		
		C-stat	CS	CIL	C-stat	CS	CIL
ASCVD	statin naïve	0.682 (0.678;0.686)	0.4 (0.388;0.411)	-0.602 (-0.622;-0.581)	0.704 (0.7;0.708)	0.481 (0.469;0.492)	-0.128 (-0.148;-0.109)
	statin users	0.624 (0.617;0.631)	0.334 (0.312;0.356)	-0.72 (-0.749;-0.692)	0.647 (0.641;0.654)	0.417 (0.396;0.438)	-0.325 (-0.351;-0.299)
CHD Advanced	statin naïve	0.677 (0.673;0.681)	0.633 (0.616;0.649)	-0.503 (-0.52;-0.486)	0.706 (0.703;0.71)	0.772 (0.756;0.788)	-0.087 (-0.102;-0.071)
	statin users	0.607 (0.6;0.614)	0.469 (0.436;0.502)	-0.595 (-0.621;-0.568)	0.64 (0.633;0.646)	0.625 (0.594;0.656)	-0.238 (-0.262;-0.213)
CHS Basic	statin naïve	0.677 (0.673;0.681)	0.745 (0.726;0.765)	-0.585 (-0.602;-0.568)	0.706 (0.702;0.709)	0.906 (0.887;0.924)	-0.185 (-0.2;-0.169)
	statin users	0.607 (0.6;0.614)	0.546 (0.508;0.585)	-0.66 (-0.687;-0.634)	0.639 (0.633;0.645)	0.726 (0.69;0.762)	-0.312 (-0.336;-0.288)
DARTS	statin naïve	0.668 (0.664;0.673)	0.527 (0.505;0.55)	-1.066 (-1.087;-1.046)	0.686 (0.683;0.69)	0.617 (0.598;0.637)	-0.646 (-0.666;-0.627)
	statin users	0.6 (0.592;0.607)	0.394 (0.361;0.427)	-1.004 (-1.031;-0.977)	0.615 (0.608;0.622)	0.471 (0.439;0.503)	-0.645 (-0.67;-0.62)
Finrisk CHD	statin naïve	0.681 (0.677;0.686)	0.497 (0.482;0.512)	0.397 (0.376;0.419)	0.701 (0.697;0.705)	0.565 (0.552;0.579)	0.845 (0.824;0.865)
	statin users	0.614 (0.607;0.622)	0.394 (0.367;0.421)	0.413 (0.385;0.441)	0.634 (0.627;0.641)	0.467 (0.442;0.492)	0.788 (0.763;0.814)
Finrisk CVD	statin naïve	0.685 (0.68;0.689)	0.532 (0.518;0.547)	0.452 (0.432;0.472)	0.707 (0.703;0.71)	0.614 (0.6;0.628)	0.896 (0.877;0.915)
	statin users	0.62 (0.612;0.627)	0.426 (0.398;0.453)	0.403 (0.375;0.43)	0.643 (0.636;0.649)	0.518 (0.492;0.544)	0.776 (0.751;0.801)
Finrisk Stroke	statin naïve	0.68 (0.676;0.684)	0.49 (0.476;0.503)	0.524 (0.504;0.543)	0.704 (0.701;0.708)	0.576 (0.563;0.588)	0.976 (0.958;0.993)
	statin users	0.617 (0.61;0.624)	0.377 (0.352;0.402)	0.406 (0.378;0.433)	0.642 (0.636;0.648)	0.471 (0.447;0.494)	0.788 (0.762;0.814)
Framingham 1991 CVD	statin naïve	0.641 (0.637;0.646)	0.521 (0.501;0.541)	-1.892 (-1.915;-1.87)	0.649 (0.644;0.653)	0.551 (0.533;0.568)	-1.483 (-1.505;-1.462)
	statin users	0.597 (0.59;0.605)	0.411 (0.378;0.443)	-1.875 (-1.902;-1.848)	0.601 (0.594;0.608)	0.428 (0.399;0.458)	-1.515 (-1.539;-1.49)
Framingham 1991 fatal CHD	statin naïve	0.657 (0.652;0.661)	0.51 (0.49;0.53)	0.393 (0.371;0.415)	0.669 (0.665;0.673)	0.549 (0.531;0.566)	0.801 (0.78;0.822)
	statin users	0.602 (0.595;0.61)	0.416 (0.383;0.449)	0.389 (0.362;0.416)	0.61 (0.603;0.617)	0.452 (0.422;0.482)	0.745 (0.72;0.769)
Framingham 1991 Stroke	statin naïve	0.66 (0.656;0.665)	0.374 (0.359;0.388)	-0.14 (-0.165;-0.114)	0.678 (0.674;0.682)	0.43 (0.417;0.444)	0.327 (0.304;0.35)
	statin users	0.608 (0.601;0.615)	0.305 (0.284;0.327)	-0.223 (-0.251;-0.194)	0.624 (0.618;0.63)	0.358 (0.338;0.378)	0.177 (0.151;0.204)
Framingham 1998	statin naïve	0.68 (0.676;0.684)	0.777 (0.756;0.798)	-0.139 (-0.157;-0.121)	0.706 (0.702;0.71)	0.923 (0.903;0.943)	0.259 (0.242;0.275)
	statin users	0.614 (0.607;0.621)	0.602 (0.562;0.641)	-0.163 (-0.19;-0.137)	0.64 (0.634;0.647)	0.754 (0.716;0.791)	0.184 (0.16;0.208)

QRISK2	statin naïve	0.678 (0.674;0.683)	0.62 (0.6;0.64)	-0.597 (-0.616;-0.578)	0.699 (0.695;0.703)	0.718 (0.7;0.737)	-0.186 (-0.204;-0.168)
	statin users	0.619 (0.612;0.626)	0.512 (0.476;0.548)	-0.744 (-0.771;-0.718)	0.638 (0.631;0.644)	0.611 (0.577;0.645)	-0.39 (-0.415;-0.366)
QRISK3	statin naïve	0.678 (0.674;0.683)	0.647 (0.626;0.668)	-0.414 (-0.433;-0.395)	0.698 (0.695;0.702)	0.744 (0.724;0.763)	-0.007 (-0.024;0.01)
	statin users	0.62 (0.613;0.627)	0.56 (0.52;0.601)	-0.491 (-0.517;-0.464)	0.638 (0.632;0.645)	0.664 (0.626;0.703)	-0.142 (-0.166;-0.117)
RECODE	statin naïve	0.654 (0.649;0.659)	0.776 (0.747;0.804)	-0.219 (-0.236;-0.202)	0.67 (0.665;0.674)	0.888 (0.862;0.914)	0.166 (0.151;0.182)
	statin users	0.6 (0.592;0.608)	0.579 (0.527;0.632)	-0.147 (-0.173;-0.12)	0.616 (0.609;0.624)	0.692 (0.644;0.741)	0.195 (0.171;0.219)
Reynolds Risk	statin naïve	0.678 (0.674;0.682)	0.481 (0.467;0.496)	0.39 (0.368;0.412)	0.696 (0.692;0.7)	0.545 (0.532;0.558)	0.837 (0.817;0.857)
	statin users	0.614 (0.606;0.621)	0.386 (0.359;0.413)	0.432 (0.404;0.459)	0.631 (0.624;0.638)	0.45 (0.425;0.475)	0.807 (0.782;0.833)
SCORE CHD	statin naïve	0.686 (0.682;0.69)	0.433 (0.421;0.446)	2.265 (2.245;2.285)	0.706 (0.702;0.709)	0.485 (0.474;0.497)	2.72 (2.701;2.739)
	statin users	0.616 (0.609;0.623)	0.356 (0.332;0.38)	2.24 (2.212;2.267)	0.637 (0.63;0.643)	0.423 (0.4;0.445)	2.617 (2.592;2.642)
SCORE CVD	statin naïve	0.687 (0.683;0.691)	0.415 (0.404;0.427)	1.667 (1.647;1.687)	0.71 (0.707;0.714)	0.474 (0.463;0.485)	2.135 (2.116;2.154)
	statin users	0.619 (0.612;0.626)	0.351 (0.328;0.374)	1.606 (1.578;1.634)	0.644 (0.637;0.65)	0.428 (0.407;0.45)	1.989 (1.964;2.015)
UKPDS 56	statin naïve	0.655 (0.651;0.66)	0.477 (0.46;0.493)	-0.43 (-0.453;-0.406)	0.665 (0.661;0.669)	0.522 (0.506;0.537)	-0.021 (-0.021;0.022)
	statin users	0.593 (0.585;0.601)	0.34 (0.31;0.37)	-0.27 (-0.298;-0.243)	0.6 (0.593;0.607)	0.371 (0.344;0.398)	0.101 (0.076;0.127)
UKPDS 68 C-HF	statin naïve	0.659 (0.655;0.664)	0.414 (0.4;0.427)	0.902 (0.877;0.928)	0.691 (0.687;0.695)	0.515 (0.501;0.528)	1.359 (1.336;1.383)
	statin users	0.597 (0.59;0.605)	0.304 (0.278;0.331)	0.819 (0.785;0.853)	0.632 (0.625;0.639)	0.423 (0.396;0.45)	1.204 (1.173;1.235)
UKPDS 68 Stroke	statin naïve	0.659 (0.655;0.664)	0.414 (0.4;0.427)	0.902 (0.877;0.928)	0.691 (0.687;0.695)	0.515 (0.501;0.528)	1.359 (1.336;1.383)
	statin users	0.597 (0.59;0.605)	0.304 (0.278;0.331)	0.819 (0.785;0.853)	0.632 (0.625;0.639)	0.423 (0.396;0.45)	1.204 (1.173;1.235)
UKPDS 82 C-HF	statin naïve	0.648 (0.642;0.653)	0.467 (0.444;0.489)	0.291 (0.261;0.32)	0.679 (0.674;0.683)	0.586 (0.563;0.609)	0.715 (0.688;0.742)
	statin users	0.588 (0.58;0.596)	0.318 (0.282;0.354)	0.182 (0.148;0.216)	0.621 (0.614;0.629)	0.451 (0.415;0.488)	0.549 (0.518;0.58)
UKPDS 82 CHD	statin naïve	0.632 (0.626;0.637)	0.451 (0.432;0.47)	-0.383 (-0.402;-0.365)	0.655 (0.65;0.66)	0.548 (0.529;0.566)	0.036 (0.019;0.053)
	statin users	0.578 (0.571;0.586)	0.311 (0.28;0.341)	-0.378 (-0.405;-0.35)	0.599 (0.592;0.607)	0.397 (0.368;0.425)	-0.01 (-0.035;0.015)

ESM Table 20 Clinical characteristics of the prediction score variables around the time (1 year before to 1 week after) of type 2 diabetes diagnosis (includes all type 2 diabetes patients irrespective of their baseline CVD status).

Clinical characteristics	Mean (SD) or N (%)	Median (Q1; Q3)	Missing data (%)
Total no. of individuals	203,172		
Follow-up time (years)		10.0 (6.6; 10.0)	
History of CVD	32,440 (16)		
Women (%)	91,008 (44.8)		0.0
Age (years)	60.9 (14.0)	61.0 (51.0; 71.0)	0.0
HbA_{1c} (mmol/mol)<br>HbA_{1c} (%)	63.3 (20.2)<br>7.9 (4)	56.3 (48.6; 74.0)<br>7.3 (6.6; 8.9)	55.0
FPG (mmol / L)	9.5 (3.9)	8.0 (7.1; 10.6)	68.5
BMI (kg / m^2)	31.7 (6.7)	30.7 (27.1; 35.2)	39.9
HDL cholesterol (mmol / L)	1.2 (0.4)	1.2 (1.0; 1.4)	47.4
LDL cholesterol (mmol / L)	3.1 (1.1)	3.0 (2.3; 3.8)	58.8
Total cholesterol (mmol / L)	5.3 (1.3)	5.2 (4.4; 6.1)	36.9
SBP (mmHg)	140 (18)	140 (130; 150)	24.8
Statin usage (before type 2 diabetes diagnosis)	64,292 (31.6)		
Smoking status			22.7
Never smoked	78,241 (38.5)		
Ex-smoker	46,944 (23.1)		
Current smoker	31,847 (15.7)		
Townsend score			0.0
1 (least deprived)	38,192 (18.8)		
2	42,253 (20.8)		
3	42,577 (21.0)		
4	45,312 (22.3)		
5 (most deprived)	34,692 (17.1)		

ESM Table 21 Discrimination (c-statistic) and calibration (calibration-in-the-large (CIL) and calibration slope (CS)) of the risk scores against the CVD and CVD + AF + HF outcomes for the imputed dataset (includes all type 2 diabetes patients irrespective of their CVD history at baseline) stratified by age subgroups.

Risk Score Name	Age-group (years)	**CVD**			**CVD + AF + HF**		
		**C-stat**	**CS**	**CIL**	**C-stat**	**CS**	**CIL**
ASCVD	[18, 50)	0.615 (0.605;0.625)	1.248 (0.83;1.665)	0.015 (-0.019;0.048)	0.604 (0.595;0.613)	1.286 (0.888;1.685)	0.012 (-0.019;0.043)
	[50, 65)	0.593 (0.588;0.599)	0.941 (0.844;1.038)	-0.018 (-0.037;0.001)	0.592 (0.587;0.597)	0.953 (0.854;1.052)	-0.011 (-0.029;0.007)
	[65, 75)	0.573 (0.567;0.579)	1.062 (0.929;1.195)	-0.043 (-0.064;-0.023)	0.573 (0.567;0.579)	1.165 (0.993;1.338)	-0.043 (-0.062;-0.023)
	[75, 105]	0.529 (0.522;0.536)	0.63 (0.307;0.952)	0.076 (0.054;0.098)	0.546 (0.539;0.552)	1.234 (0.877;1.591)	0.057 (0.035;0.079)
	[18, 50)	0.629 (0.619;0.639)	1.094 (0.898;1.289)	0.017 (-0.018;0.051)	0.619 (0.61;0.628)	1.118 (0.927;1.309)	0.014 (-0.017;0.046)
	[50, 65)	0.572 (0.566;0.578)	1.073 (0.884;1.261)	-0.018 (-0.036;0.001)	0.571 (0.565;0.576)	1.062 (0.879;1.246)	-0.011 (-0.028;0.007)
	[65, 75)	0.54 (0.533;0.546)	0.967 (0.718;1.217)	-0.038 (-0.058;-0.018)	0.538 (0.532;0.545)	1.009 (0.698;1.319)	-0.038 (-0.057;-0.019)
Framingham 1991 fatal CHD	[75, 105]	0.516 (0.508;0.523)	1.212 (-0.17;2.593)	0.076 (0.054;0.098)	0.521 (0.514;0.528)	0.922 (0.409;1.435)	0.057 (0.035;0.079)
	[18, 50)	0.623 (0.613;0.633)	1.097 (0.951;1.243)	0.017 (-0.017;0.052)	0.614 (0.605;0.623)	1.136 (0.958;1.313)	0.015 (-0.017;0.046)
	[50, 65)	0.583 (0.577;0.589)	1.057 (0.881;1.233)	-0.017 (-0.036;0.002)	0.581 (0.575;0.586)	1.056 (0.882;1.231)	-0.01 (-0.028;0.008)
	[65, 75)	0.557 (0.551;0.563)	1.069 (0.895;1.243)	-0.038 (-0.058;-0.018)	0.553 (0.547;0.559)	1.081 (0.863;1.299)	-0.038 (-0.057;-0.018)
Framingham 1991 CVD	[75, 105]	0.535 (0.528;0.542)	1.092 (0.62;1.564)	0.076 (0.054;0.099)	0.537 (0.53;0.543)	0.988 (0.663;1.314)	0.058 (0.036;0.08)
	[18, 50)	0.628 (0.619;0.638)	1.023 (0.93;1.116)	0.02 (-0.014;0.055)	0.62 (0.611;0.63)	1.061 (0.943;1.18)	0.017 (-0.014;0.049)
	[50, 65)	0.576 (0.57;0.582)	1.132 (0.941;1.324)	-0.019 (-0.038;0)	0.576 (0.57;0.583)	1.087 (0.916;1.257)	-0.012 (-0.029;0.006)
	[65, 75)	0.543 (0.537;0.55)	1.125 (0.849;1.4)	-0.039 (-0.059;-0.018)	0.545 (0.539;0.551)	1.069 (0.805;1.334)	-0.039 (-0.058;-0.019)
Framingham 1991 Stroke	[75, 105]	0.526 (0.519;0.533)	1.117 (0.603;1.632)	0.076 (0.054;0.098)	0.534 (0.527;0.541)	1.132 (0.697;1.566)	0.057 (0.035;0.079)
	[18, 50)	0.63 (0.621;0.639)	1.138 (1.047;1.229)	0.011 (-0.023;0.044)	0.62 (0.612;0.628)	1.158 (1.067;1.249)	0.009 (-0.022;0.04)
CHS Advanced	[50, 65)	0.585 (0.58;0.59)	1.011 (0.939;1.083)	-0.021 (-0.039;-0.002)	0.589 (0.584;0.594)	1.064 (0.997;1.131)	-0.014 (-0.031;0.004)

	[65, 75)	0.567 (0.561;0.574)	1.007 (0.893;1.121)	-0.035 (-0.055;-0.015)	0.572 (0.566;0.578)	1.083 (0.972;1.194)	-0.035 (-0.054;-0.015)
	[75, 105]	0.533 (0.526;0.539)	0.766 (0.538;0.994)	0.077 (0.054;0.099)	0.551 (0.544;0.557)	1.16 (0.936;1.384)	0.058 (0.036;0.08)
	[18, 50)	0.629 (0.62;0.638)	1.139 (1.045;1.233)	0.011 (-0.023;0.044)	0.619 (0.61;0.627)	1.156 (1.063;1.25)	0.009 (-0.022;0.04)
	[50, 65)	0.587 (0.582;0.592)	0.997 (0.922;1.072)	-0.02 (-0.039;-0.001)	0.59 (0.585;0.595)	1.051 (0.981;1.12)	-0.013 (-0.031;0.005)
	[65, 75)	0.569 (0.562;0.575)	1.008 (0.884;1.131)	-0.034 (-0.054;-0.014)	0.571 (0.565;0.577)	1.072 (0.951;1.193)	-0.034 (-0.053;-0.014)
CHS Basic	[75, 105]	0.536 (0.53;0.543)	0.799 (0.584;1.014)	0.077 (0.055;0.099)	0.552 (0.546;0.559)	1.144 (0.913;1.374)	0.058 (0.036;0.08)
	[18, 50)	0.62 (0.61;0.631)	1.235 (1.018;1.451)	0.008 (-0.025;0.042)	0.61 (0.601;0.62)	1.198 (1.003;1.394)	0.007 (-0.024;0.039)
	[50, 65)	0.542 (0.535;0.548)	0.919 (0.525;1.314)	-0.021 (-0.04;-0.003)	0.542 (0.535;0.549)	0.974 (0.555;1.394)	-0.014 (-0.032;0.004)
	[65, 75)	0.509 (0.491;0.526)	-0.452 (-10.744;9.839)	-0.038 (-0.058;-0.018)	0.512 (0.506;0.518)	2.436 (-2.858;7.731)	-0.038 (-0.057;-0.019)
DARTS	[75, 105]	0.502 (0.489;0.515)	0.477 (-3.223;4.177)	0.077 (0.054;0.099)	0.502 (0.488;0.515)	-0.487 (-5.277;4.304)	0.058 (0.036;0.079)
	[18, 50)	0.632 (0.623;0.642)	1.13 (0.964;1.296)	0.018 (-0.016;0.052)	0.622 (0.613;0.63)	1.192 (1.024;1.36)	0.015 (-0.016;0.046)
	[50, 65)	0.566 (0.56;0.571)	0.959 (0.806;1.111)	-0.02 (-0.038;-0.001)	0.567 (0.562;0.573)	0.947 (0.805;1.089)	-0.012 (-0.03;0.006)
	[65, 75)	0.534 (0.527;0.541)	0.972 (0.685;1.259)	-0.041 (-0.061;-0.021)	0.535 (0.529;0.542)	1.163 (0.8;1.526)	-0.04 (-0.059;-0.021)
Finrisk CHD	[75, 105]	0.509 (0.501;0.516)	0.807 (-0.498;2.112)	0.076 (0.054;0.098)	0.521 (0.514;0.528)	0.923 (0.379;1.467)	0.057 (0.035;0.078)
	[18, 50)	0.63 (0.621;0.64)	1.052 (0.91;1.194)	0.021 (-0.013;0.054)	0.622 (0.613;0.631)	1.103 (0.955;1.25)	0.017 (-0.014;0.048)
	[50, 65)	0.581 (0.575;0.586)	0.989 (0.869;1.11)	-0.02 (-0.038;-0.001)	0.583 (0.578;0.588)	0.987 (0.873;1.101)	-0.012 (-0.03;0.005)
	[65, 75)	0.565 (0.559;0.572)	1.075 (0.925;1.225)	-0.042 (-0.063;-0.022)	0.563 (0.557;0.569)	1.194 (1.007;1.381)	-0.042 (-0.061;-0.022)
Finrisk CVD	[75, 105]	0.54 (0.533;0.547)	0.869 (0.649;1.089)	0.076 (0.054;0.098)	0.549 (0.542;0.556)	0.938 (0.726;1.15)	0.057 (0.035;0.079)
	[18, 50)	0.593 (0.584;0.602)	0.88 (0.771;0.99)	0.014 (-0.019;0.048)	0.59 (0.581;0.599)	0.942 (0.812;1.072)	0.012 (-0.019;0.043)
	[50, 65)	0.591 (0.585;0.596)	1.05 (0.961;1.139)	-0.021 (-0.039;-0.002)	0.594 (0.589;0.599)	1.051 (0.965;1.138)	-0.013 (-0.031;0.004)
	[65, 75)	0.585 (0.579;0.591)	1.14 (1.039;1.241)	-0.042 (-0.062;-0.022)	0.581 (0.575;0.586)	1.227 (1.11;1.343)	-0.041 (-0.061;-0.022)
Finrisk Stroke	[75, 105]	0.557 (0.55;0.563)	0.903 (0.767;1.039)	0.077 (0.055;0.099)	0.559 (0.552;0.566)	0.952 (0.809;1.094)	0.058 (0.036;0.08)
Framingham 1998	[18, 50)	0.631 (0.621;0.64)	1.073 (0.947;1.198)	0.022 (-0.012;0.056)	0.624 (0.615;0.634)	1.106 (0.963;1.249)	0.02 (-0.012;0.051)

	[50, 65)	0.568 (0.561;0.574)	1.161 (0.92;1.402)	-0.021 (-0.04;-0.003)	0.572 (0.566;0.578)	1.127 (0.926;1.328)	-0.014 (-0.032;0.003)
	[65, 75)	0.539 (0.532;0.546)	1.219 (0.913;1.525)	-0.04 (-0.059;-0.02)	0.545 (0.538;0.552)	1.311 (0.918;1.705)	-0.04 (-0.059;-0.021)
	[75, 105]	0.516 (0.509;0.522)	0.924 (0.16;1.687)	0.076 (0.054;0.098)	0.533 (0.526;0.54)	1.181 (0.742;1.62)	0.057 (0.035;0.079)
	[18, 50)	0.658 (0.648;0.668)	1.053 (0.906;1.199)	0.022 (-0.012;0.056)	0.646 (0.638;0.655)	1.049 (0.916;1.183)	0.02 (-0.012;0.051)
	[50, 65)	0.605 (0.6;0.61)	0.984 (0.863;1.104)	-0.02 (-0.039;-0.002)	0.603 (0.598;0.608)	0.985 (0.863;1.106)	-0.013 (-0.031;0.005)
	[65, 75)	0.562 (0.556;0.569)	1.094 (0.844;1.344)	-0.04 (-0.06;-0.019)	0.562 (0.556;0.568)	1.211 (0.889;1.533)	-0.039 (-0.059;-0.02)
QRISK 2	[75, 105]	0.514 (0.507;0.521)	0.402 (0.077;0.728)	0.075 (0.053;0.098)	0.527 (0.52;0.534)	0.639 (0.416;0.863)	0.056 (0.034;0.078)
	[18, 50)	0.654 (0.644;0.663)	1.051 (0.901;1.201)	0.024 (-0.01;0.058)	0.643 (0.634;0.652)	1.04 (0.907;1.174)	0.022 (-0.01;0.054)
	[50, 65)	0.594 (0.589;0.6)	1.02 (0.881;1.16)	-0.02 (-0.039;-0.001)	0.594 (0.589;0.599)	0.989 (0.857;1.12)	-0.013 (-0.031;0.005)
QRISK 3	[65, 75)	0.553 (0.546;0.559)	1.069 (0.791;1.347)	-0.04 (-0.06;-0.019)	0.554 (0.548;0.56)	1.132 (0.817;1.448)	-0.04 (-0.059;-0.02)
	[75, 105]	0.512 (0.505;0.519)	0.506 (-0.005;1.018)	0.076 (0.054;0.098)	0.524 (0.517;0.531)	0.815 (0.441;1.189)	0.056 (0.035;0.078)
	[18, 50)	0.654 (0.644;0.663)	1.105 (0.955;1.254)	0.006 (-0.029;0.041)	0.638 (0.628;0.647)	1.099 (0.964;1.235)	0.005 (-0.027;0.037)
	[50, 65)	0.674 (0.667;0.681)	0.992 (0.908;1.076)	-0.018 (-0.038;0.003)	0.659 (0.652;0.665)	1.019 (0.932;1.107)	-0.01 (-0.029;0.009)
	[65, 75)	0.682 (0.676;0.688)	1.003 (0.947;1.058)	-0.042 (-0.067;-0.017)	0.66 (0.655;0.666)	1.008 (0.95;1.067)	-0.041 (-0.064;-0.018)
RECODE	[75, 105]	0.652 (0.645;0.659)	0.888 (0.776;0.999)	0.079 (0.055;0.103)	0.64 (0.633;0.647)	0.946 (0.821;1.072)	0.058 (0.035;0.082)
	[18, 50)	0.632 (0.623;0.642)	1.039 (0.899;1.18)	0.018 (-0.016;0.052)	0.623 (0.614;0.632)	1.094 (0.974;1.214)	0.015 (-0.017;0.046)
	[50, 65)	0.57 (0.564;0.576)	1.027 (0.873;1.18)	-0.02 (-0.038;-0.001)	0.571 (0.566;0.577)	0.994 (0.844;1.143)	-0.012 (-0.03;0.006)
	[65, 75)	0.544 (0.538;0.551)	0.951 (0.736;1.166)	-0.041 (-0.061;-0.021)	0.543 (0.537;0.549)	1.068 (0.75;1.386)	-0.041 (-0.06;-0.021)
Reynolds Risk	[75, 105]	0.517 (0.51;0.524)	0.902 (0.232;1.572)	0.076 (0.054;0.098)	0.527 (0.52;0.534)	0.981 (0.368;1.593)	0.057 (0.035;0.079)
	[18, 50)	0.645 (0.636;0.654)	1.236 (1.121;1.351)	0.015 (-0.019;0.048)	0.632 (0.623;0.64)	1.253 (1.137;1.369)	0.012 (-0.019;0.043)
	[50, 65)	0.584 (0.578;0.589)	0.946 (0.841;1.05)	-0.019 (-0.038;0)	0.583 (0.578;0.588)	0.939 (0.84;1.039)	-0.012 (-0.029;0.006)
	[65, 75)	0.547 (0.541;0.553)	1.045 (0.826;1.265)	-0.04 (-0.06;-0.02)	0.547 (0.541;0.553)	1.188 (0.874;1.502)	-0.04 (-0.059;-0.02)
SCORE CHD	[75, 105]	0.512 (0.506;0.519)	0.933 (0.178;1.689)	0.075 (0.053;0.097)	0.525 (0.518;0.531)	0.973 (0.569;1.377)	0.056 (0.034;0.078)

	[18, 50)	0.649 (0.64;0.658)	1.205 (1.101;1.309)	0.016 (-0.018;0.05)	0.636 (0.628;0.645)	1.221 (1.113;1.328)	0.013 (-0.018;0.045)
	[50, 65)	0.587 (0.582;0.593)	0.985 (0.88;1.09)	-0.019 (-0.038;-0.001)	0.589 (0.583;0.594)	0.971 (0.873;1.07)	-0.012 (-0.03;0.005)
	[65, 75)	0.549 (0.542;0.555)	1.024 (0.822;1.226)	-0.042 (-0.062;-0.021)	0.551 (0.545;0.557)	1.158 (0.893;1.423)	-0.041 (-0.06;-0.022)
SCORE CVD	[75, 105]	0.515 (0.509;0.522)	0.828 (0.373;1.283)	0.075 (0.053;0.097)	0.53 (0.523;0.536)	1.012 (0.681;1.343)	0.056 (0.033;0.078)
	[18, 50)	0.602 (0.592;0.612)	1.191 (0.911;1.471)	0.009 (-0.024;0.043)	0.591 (0.582;0.601)	1.214 (0.982;1.445)	0.007 (-0.024;0.039)
	[50, 65)	0.558 (0.551;0.564)	0.96 (0.768;1.151)	-0.019 (-0.038;0)	0.556 (0.55;0.563)	0.97 (0.782;1.158)	-0.012 (-0.03;0.006)
	[65, 75)	0.542 (0.535;0.549)	1.046 (0.782;1.309)	-0.04 (-0.06;-0.02)	0.538 (0.531;0.544)	1.173 (0.818;1.529)	-0.039 (-0.058;-0.02)
UKPDS 56	[75, 105]	0.522 (0.514;0.529)	0.871 (0.367;1.375)	0.076 (0.054;0.099)	0.527 (0.52;0.534)	0.84 (0.503;1.178)	0.057 (0.035;0.079)
	[18, 50)	0.58 (0.57;0.59)	0.92 (0.744;1.095)	0.01 (-0.024;0.044)	0.583 (0.573;0.592)	0.88 (0.732;1.028)	0.01 (-0.022;0.042)
	[50, 65)	0.531 (0.525;0.537)	2.013 (0.394;3.632)	-0.021 (-0.039;-0.003)	0.544 (0.539;0.55)	1.275 (0.75;1.8)	-0.014 (-0.031;0.004)
	[65, 75)	0.514 (0.506;0.521)	0.628 (0.006;1.249)	-0.039 (-0.059;-0.019)	0.527 (0.52;0.535)	0.918 (0.212;1.624)	-0.04 (-0.06;-0.021)
UKPDS 68 C-HF	[75, 105]	0.507 (0.498;0.515)	0.609 (-0.763;1.98)	0.076 (0.054;0.098)	0.528 (0.52;0.537)	1.379 (0.117;2.641)	0.057 (0.035;0.079)
	[18, 50)	0.58 (0.57;0.59)	0.92 (0.744;1.095)	0.01 (-0.024;0.044)	0.583 (0.573;0.592)	0.88 (0.732;1.028)	0.01 (-0.022;0.042)
	[50, 65)	0.531 (0.525;0.537)	2.013 (0.394;3.632)	-0.021 (-0.039;-0.003)	0.544 (0.539;0.55)	1.275 (0.75;1.8)	-0.014 (-0.031;0.004)
	[65, 75)	0.514 (0.506;0.521)	0.628 (0.006;1.249)	-0.039 (-0.059;-0.019)	0.527 (0.52;0.535)	0.918 (0.212;1.624)	-0.04 (-0.06;-0.021)
UKPDS 68 Stroke	[75, 105]	0.507 (0.498;0.515)	0.609 (-0.763;1.98)	0.076 (0.054;0.098)	0.528 (0.52;0.537)	1.379 (0.117;2.641)	0.057 (0.035;0.079)
	[18, 50)	0.58 (0.57;0.591)	1.052 (0.761;1.344)	0.01 (-0.024;0.044)	0.582 (0.573;0.592)	0.959 (0.758;1.16)	0.011 (-0.021;0.043)
	[50, 65)	0.528 (0.52;0.536)	1.286 (0.867;1.706)	-0.021 (-0.04;-0.003)	0.541 (0.533;0.548)	1.082 (0.826;1.338)	-0.014 (-0.032;0.004)
	[65, 75)	0.507 (0.498;0.515)	0.474 (-0.085;1.034)	-0.038 (-0.058;-0.018)	0.522 (0.514;0.53)	1.137 (0.365;1.908)	-0.038 (-0.057;-0.019)
UKPDS 82 C-HF	[75, 105]	0.497 (0.489;0.505)	-0.429 (-1.748;0.891)	0.076 (0.054;0.098)	0.519 (0.51;0.527)	0.874 (0.32;1.429)	0.057 (0.035;0.079)
	[18, 50)	0.557 (0.546;0.567)	0.822 (0.642;1.003)	0.006 (-0.027;0.04)	0.555 (0.545;0.565)	0.745 (0.603;0.887)	0.006 (-0.026;0.037)
	[50, 65)	0.519 (0.51;0.527)	1.925 (0.693;3.156)	-0.022 (-0.04;-0.003)	0.524 (0.515;0.533)	1.362 (0.908;1.816)	-0.015 (-0.032;0.003)
UKPDS 82 CHD	[65, 75)	0.513 (0.504;0.521)	0.713 (0.459;0.966)	-0.038 (-0.058;-0.017)	0.52 (0.512;0.528)	0.951 (0.709;1.193)	-0.038 (-0.057;-0.018)

	[75, 105]	0.515 (0.508;0.523)	0.779 (0.49;1.069)	0.074 (0.052;0.096)	0.531 (0.524;0.538)	1.297 (0.942;1.652)	0.055 (0.033;0.077)

ESM Table 22 Discrimination (c-statistic) and calibration (calibration-in-the-large (CIL) and calibration slope (CS)) of the risk scores against the CVD and CVD + AF + HF outcomes for the imputed dataset stratified by CVD history at baseline (overall vs absent vs present).

Risk Score Name	CVD history at baseline (overall vs absent vs present)	CVD			CVD + AF + HF		
		C-stat	CS	CIL	C-stat	CS	CIL
	overall	0.677 (0.674;0.68)	0.411 (0.402;0.419)	-0.153 (-0.167;-0.139)	0.697 (0.695;0.7)	0.489 (0.48;0.497)	0.213 (0.199;0.227)
	absent	0.668 (0.664;0.671)	0.383 (0.373;0.393)	-0.639 (-0.656;-0.622)	0.689 (0.686;0.693)	0.461 (0.451;0.471)	-0.191 (-0.207;-0.176)
ASCVD	present	0.52 (0.513;0.528)	0.277 (0.077;0.477)	0.01 (-0.014;0.035)	0.552 (0.544;0.56)	0.626 (0.51;0.743)	0.047 (0.021;0.073)
	overall	0.68 (0.678;0.683)	0.677 (0.664;0.689)	-0.087 (-0.099;-0.075)	0.705 (0.702;0.707)	0.799 (0.787;0.811)	0.237 (0.226;0.248)
	absent	0.66 (0.656;0.663)	0.595 (0.58;0.61)	-0.53 (-0.545;-0.516)	0.689 (0.686;0.692)	0.731 (0.717;0.745)	-0.134 (-0.147;-0.12)
CHS Advanced	present	0.513 (0.506;0.52)	0.364 (0.136;0.592)	0.011 (-0.013;0.035)	0.547 (0.54;0.554)	0.752 (0.627;0.876)	0.048 (0.023;0.074)
	overall	0.681 (0.678;0.684)	0.8 (0.786;0.815)	-0.165 (-0.176;-0.153)	0.705 (0.702;0.707)	0.94 (0.926;0.954)	0.146 (0.135;0.158)
	absent	0.66 (0.656;0.663)	0.699 (0.682;0.716)	-0.607 (-0.621;-0.593)	0.688 (0.685;0.692)	0.856 (0.839;0.872)	-0.224 (-0.237;-0.211)
CHS Basic	present	0.515 (0.507;0.522)	0.406 (0.199;0.612)	0.011 (-0.013;0.035)	0.548 (0.54;0.555)	0.748 (0.626;0.87)	0.048 (0.022;0.074)
	overall	0.643 (0.64;0.646)	0.495 (0.481;0.509)	-0.532 (-0.546;-0.518)	0.662 (0.659;0.665)	0.588 (0.574;0.602)	-0.211 (-0.224;-0.197)
	absent	0.651 (0.647;0.655)	0.5 (0.48;0.52)	-1.049 (-1.066;-1.032)	0.668 (0.665;0.672)	0.585 (0.567;0.603)	-0.65 (-0.665;-0.634)
DARTS	present	0.516 (0.507;0.524)	0.39 (0.095;0.685)	0.013 (-0.011;0.038)	0.542 (0.533;0.55)	0.723 (0.47;0.976)	0.053 (0.027;0.078)
	overall	0.661 (0.658;0.664)	0.476 (0.466;0.486)	0.913 (0.898;0.928)	0.682 (0.679;0.684)	0.547 (0.537;0.557)	1.259 (1.244;1.273)
	absent	0.665 (0.661;0.668)	0.475 (0.462;0.488)	0.401 (0.383;0.418)	0.684 (0.681;0.688)	0.542 (0.53;0.554)	0.824 (0.807;0.841)
Finrisk CHD	present	0.52 (0.513;0.528)	0.514 (0.296;0.732)	0.011 (-0.013;0.036)	0.548 (0.54;0.556)	0.788 (0.617;0.959)	0.05 (0.024;0.075)
	overall	0.677 (0.674;0.68)	0.543 (0.532;0.553)	0.913 (0.899;0.927)	0.697 (0.694;0.7)	0.619 (0.609;0.629)	1.258 (1.245;1.271)
	absent	0.668 (0.665;0.672)	0.509 (0.496;0.521)	0.437 (0.42;0.453)	0.69 (0.687;0.694)	0.589 (0.577;0.601)	0.857 (0.842;0.872)
Finrisk CVD	present	0.527 (0.52;0.535)	0.65 (0.468;0.831)	0.011 (-0.014;0.035)	0.554 (0.546;0.562)	0.843 (0.7;0.986)	0.048 (0.023;0.074)
	overall	0.683 (0.68;0.686)	0.522 (0.513;0.532)	0.944 (0.931;0.957)	0.703 (0.7;0.705)	0.596 (0.587;0.605)	1.297 (1.284;1.309)
	absent	0.664 (0.661;0.668)	0.462 (0.451;0.474)	0.488 (0.472;0.504)	0.688 (0.685;0.691)	0.546 (0.535;0.557)	0.917 (0.902;0.932)
Finrisk Stroke	present	0.533 (0.525;0.54)	0.761 (0.591;0.931)	0.011 (-0.014;0.035)	0.556 (0.549;0.564)	0.898 (0.759;1.037)	0.049 (0.023;0.074)

	overall	0.63 (0.627;0.634)	0.514 (0.499;0.529)	-1.379 (-1.394;-1.364)	0.637 (0.634;0.641)	0.543 (0.529;0.557)	-1.061 (-1.075;-1.046)
	absent	0.63 (0.626;0.634)	0.498 (0.48;0.515)	-1.888 (-1.906;-1.869)	0.636 (0.633;0.64)	0.521 (0.506;0.536)	-1.495 (-1.513;-1.478)
Framingham 1991 CVD	present	0.538 (0.53;0.546)	0.769 (0.565;0.974)	0.015 (-0.01;0.039)	0.545 (0.537;0.554)	0.835 (0.615;1.055)	0.054 (0.028;0.079)
	overall	0.638 (0.635;0.641)	0.498 (0.485;0.511)	0.893 (0.877;0.909)	0.65 (0.647;0.653)	0.538 (0.526;0.55)	1.212 (1.197;1.227)
	absent	0.643 (0.639;0.647)	0.49 (0.473;0.508)	0.391 (0.374;0.409)	0.654 (0.65;0.657)	0.526 (0.511;0.541)	0.782 (0.765;0.799)
Framingham 1991 fatal CHD	present	0.53 (0.522;0.538)	0.814 (0.534;1.095)	0.013 (-0.011;0.038)	0.544 (0.536;0.553)	0.89 (0.641;1.138)	0.052 (0.027;0.078)
	overall	0.652 (0.649;0.655)	0.383 (0.372;0.394)	0.364 (0.347;0.38)	0.668 (0.665;0.671)	0.436 (0.425;0.446)	0.724 (0.708;0.739)
	absent	0.647 (0.644;0.651)	0.359 (0.347;0.371)	-0.165 (-0.185;-0.144)	0.664 (0.66;0.667)	0.41 (0.399;0.421)	0.28 (0.261;0.298)
Framingham 1991 Stroke	present	0.526 (0.519;0.534)	0.63 (0.422;0.838)	0.014 (-0.011;0.038)	0.545 (0.537;0.553)	0.828 (0.605;1.052)	0.053 (0.028;0.079)
	overall	0.67 (0.667;0.673)	0.773 (0.758;0.788)	0.319 (0.306;0.331)	0.694 (0.691;0.696)	0.909 (0.894;0.924)	0.628 (0.616;0.639)
	absent	0.664 (0.66;0.667)	0.738 (0.719;0.756)	-0.147 (-0.162;-0.132)	0.689 (0.686;0.692)	0.877 (0.86;0.895)	0.234 (0.22;0.248)
Framingham 1998	present	0.514 (0.507;0.521)	0.483 (0.211;0.755)	0.013 (-0.012;0.037)	0.547 (0.54;0.555)	0.862 (0.711;1.013)	0.051 (0.026;0.077)
	overall	0.675 (0.672;0.678)	0.666 (0.652;0.681)	-0.174 (-0.187;-0.161)	0.693 (0.691;0.696)	0.757 (0.744;0.771)	0.144 (0.131;0.157)
	absent	0.664 (0.66;0.667)	0.595 (0.576;0.613)	-0.64 (-0.655;-0.624)	0.683 (0.68;0.686)	0.686 (0.67;0.703)	-0.248 (-0.263;-0.234)
QRISK 2	present	0.522 (0.514;0.529)	0.459 (0.275;0.642)	0.01 (-0.015;0.034)	0.549 (0.542;0.557)	0.671 (0.519;0.822)	0.047 (0.021;0.072)
	overall	0.669 (0.666;0.672)	0.682 (0.667;0.698)	0.04 (0.028;0.053)	0.688 (0.685;0.691)	0.777 (0.763;0.792)	0.354 (0.341;0.366)
	absent	0.664 (0.66;0.668)	0.63 (0.611;0.65)	-0.436 (-0.452;-0.421)	0.683 (0.68;0.686)	0.723 (0.706;0.741)	-0.049 (-0.063;-0.034)
QRISK 3	Present	0.522 (0.515;0.53)	0.521 (0.314;0.728)	0.009 (-0.016;0.033)	0.55 (0.542;0.558)	0.723 (0.552;0.894)	0.045 (0.019;0.071)
	overall	0.731 (0.728;0.734)	1.052 (1.034;1.07)	0.078 (0.066;0.09)	0.732 (0.729;0.735)	1.098 (1.081;1.115)	0.392 (0.381;0.403)
	absent	0.64 (0.636;0.645)	0.731 (0.704;0.759)	-0.2 (-0.214;-0.185)	0.656 (0.652;0.661)	0.845 (0.82;0.87)	0.171 (0.158;0.184)
RECODE	present	0.534 (0.527;0.542)	0.52 (0.294;0.745)	0.01 (-0.014;0.035)	0.558 (0.55;0.566)	0.721 (0.525;0.917)	0.049 (0.023;0.075)
	overall	0.66 (0.657;0.663)	0.466 (0.455;0.476)	0.917 (0.902;0.932)	0.678 (0.675;0.68)	0.53 (0.52;0.539)	1.262 (1.248;1.276)
	absent	0.662 (0.658;0.666)	0.461 (0.448;0.473)	0.401 (0.384;0.419)	0.68 (0.676;0.683)	0.522 (0.511;0.534)	0.824 (0.808;0.841)
Reynolds Risk	present	0.528 (0.52;0.536)	0.56 (0.35;0.77)	0.011 (-0.014;0.035)	0.552 (0.543;0.56)	0.754 (0.547;0.961)	0.049 (0.023;0.075)
	overall	0.67 (0.667;0.673)	0.444 (0.435;0.452)	2.763 (2.749;2.776)	0.689 (0.687;0.692)	0.496 (0.487;0.504)	3.115 (3.102;3.129)
SCORE CHD	absent	0.669 (0.665;0.672)	0.416 (0.405;0.427)	2.256 (2.24;2.273)	0.688 (0.685;0.692)	0.468 (0.458;0.479)	2.685 (2.67;2.701)

	present	0.525 (0.518;0.533)	0.574 (0.422;0.725)	0.012 (-0.012;0.036)	0.552 (0.545;0.56)	0.749 (0.628;0.87)	0.051 (0.025;0.076)
SCORE CVD	overall	0.675 (0.673;0.678)	0.434 (0.426;0.443)	2.147 (2.133;2.16)	0.697 (0.695;0.7)	0.492 (0.484;0.5)	2.509 (2.496;2.522)
	absent	0.67 (0.667;0.674)	0.4 (0.39;0.411)	1.647 (1.631;1.664)	0.693 (0.69;0.696)	0.46 (0.45;0.47)	2.087 (2.072;2.102)
	present	0.522 (0.515;0.529)	0.553 (0.394;0.713)	0.012 (-0.013;0.036)	0.552 (0.545;0.56)	0.765 (0.646;0.885)	0.05 (0.024;0.076)
UKPDS 56	overall	0.633 (0.629;0.636)	0.433 (0.421;0.444)	0.146 (0.129;0.163)	0.644 (0.641;0.647)	0.483 (0.471;0.494)	0.478 (0.463;0.494)
	absent	0.639 (0.635;0.643)	0.446 (0.431;0.46)	-0.386 (-0.405;-0.367)	0.649 (0.645;0.652)	0.488 (0.475;0.501)	0.024 (0.007;0.042)
	present	0.533 (0.525;0.541)	0.647 (0.364;0.929)	0.01 (-0.014;0.035)	0.549 (0.54;0.558)	0.768 (0.511;1.025)	0.049 (0.023;0.075)
UKPDS 68 C-HF	overall	0.654 (0.651;0.657)	0.424 (0.412;0.435)	1.371 (1.351;1.391)	0.682 (0.679;0.685)	0.516 (0.504;0.529)	1.726 (1.707;1.744)
	absent	0.644 (0.64;0.648)	0.389 (0.376;0.402)	0.877 (0.853;0.9)	0.675 (0.672;0.679)	0.489 (0.475;0.502)	1.31 (1.289;1.331)
	present	0.507 (0.499;0.514)	0.361 (-0.138;0.859)	0.012 (-0.012;0.036)	0.542 (0.534;0.55)	0.883 (0.676;1.09)	0.049 (0.023;0.074)
UKPDS 68 Stroke	overall	0.654 (0.651;0.657)	0.424 (0.412;0.435)	1.371 (1.351;1.391)	0.682 (0.679;0.685)	0.516 (0.504;0.529)	1.726 (1.707;1.744)
	absent	0.644 (0.64;0.648)	0.389 (0.376;0.402)	0.877 (0.853;0.9)	0.675 (0.672;0.679)	0.489 (0.475;0.502)	1.31 (1.289;1.331)
	present	0.507 (0.499;0.514)	0.361 (-0.138;0.859)	0.012 (-0.012;0.036)	0.542 (0.534;0.55)	0.883 (0.676;1.09)	0.049 (0.023;0.074)
UKPDS 82 C-HF	overall	0.644 (0.641;0.647)	0.479 (0.458;0.499)	0.745 (0.723;0.767)	0.671 (0.668;0.674)	0.586 (0.564;0.608)	1.075 (1.054;1.095)
	absent	0.633 (0.628;0.638)	0.433 (0.411;0.455)	0.258 (0.232;0.284)	0.663 (0.659;0.668)	0.549 (0.525;0.572)	0.663 (0.64;0.687)
	present	0.504 (0.496;0.511)	0.303 (-0.267;0.874)	0.012 (-0.012;0.037)	0.539 (0.531;0.547)	0.874 (0.589;1.158)	0.049 (0.023;0.074)
UKPDS 82 CHD	overall	0.632 (0.628;0.636)	0.425 (0.412;0.438)	0.041 (0.028;0.054)	0.653 (0.65;0.657)	0.515 (0.502;0.528)	0.379 (0.367;0.392)
	absent	0.618 (0.614;0.623)	0.419 (0.402;0.436)	-0.383 (-0.399;-0.367)	0.641 (0.636;0.645)	0.51 (0.494;0.527)	0.019 (0.004;0.033)
	present	0.54 (0.533;0.548)	1.043 (0.782;1.305)	0.016 (-0.009;0.04)	0.5 (0.492;0.508)	0.056 (-2.083;2.194)	0.052 (0.027;0.078)

ESM Table 23 Discrimination (c-statistic) and calibration (calibration-in-the-large (CIL) and calibration slope (CS)) of the risk scores against the CVD and CVD + AF + HF outcomes for the imputed dataset (including all type 2 diabetes patients irrespective of CVD status at baseline) stratified by sex (male vs female).

Risk Score Name	Sex (male vs female)	CVD			CVD + AF + HF		
		C-stat	CS	CIL	C-stat	CS	CIL
ASCVD	male	0.665 (0.662;0.669)	1.025 (0.986;1.064)	-0.002 (-0.016;0.013)	0.686 (0.682;0.689)	1.003 (0.97;1.037)	0.007 (-0.007;0.021)
	female	0.686 (0.682;0.69)	0.975 (0.931;1.02)	0.031 (0.014;0.049)	0.708 (0.704;0.712)	0.939 (0.903;0.975)	0.024 (0.007;0.04)
Framingham 1991 fatal CHD	male	0.629 (0.624;0.633)	1.01 (0.95;1.07)	0 (-0.015;0.014)	0.641 (0.637;0.645)	0.992 (0.94;1.043)	0.008 (-0.006;0.021)
	female	0.65 (0.645;0.655)	1.06 (0.972;1.148)	0.034 (0.016;0.051)	0.662 (0.657;0.666)	1.01 (0.936;1.083)	0.026 (0.009;0.042)
Framingham 1991 CVD	male	0.623 (0.618;0.627)	1.022 (0.965;1.078)	-0.001 (-0.016;0.013)	0.632 (0.628;0.637)	1.001 (0.955;1.047)	0.007 (-0.007;0.021)
	female	0.631 (0.626;0.636)	1.109 (1.02;1.198)	0.032 (0.015;0.05)	0.638 (0.633;0.643)	1.06 (0.989;1.132)	0.024 (0.008;0.04)
Framingham 1991 Stroke	male	0.648 (0.644;0.652)	1.05 (0.998;1.102)	-0.002 (-0.016;0.013)	0.662 (0.658;0.666)	1.029 (0.993;1.064)	0.007 (-0.008;0.021)
	female	0.67 (0.665;0.674)	1.081 (0.991;1.171)	0.036 (0.019;0.053)	0.684 (0.679;0.688)	1.047 (0.976;1.118)	0.028 (0.012;0.045)
CHS Advanced	male	0.677 (0.673;0.68)	1.04 (1.016;1.065)	-0.005 (-0.02;0.009)	0.699 (0.695;0.702)	1.016 (0.995;1.037)	0.003 (-0.011;0.017)
	female	0.692 (0.687;0.696)	0.984 (0.957;1.011)	0.029 (0.012;0.046)	0.716 (0.713;0.72)	0.957 (0.935;0.979)	0.02 (0.004;0.037)
CHS Basic	male	0.675 (0.672;0.679)	1.04 (1.015;1.065)	-0.005 (-0.02;0.009)	0.697 (0.694;0.701)	1.015 (0.994;1.037)	0.003 (-0.011;0.017)
	female	0.691 (0.687;0.695)	0.981 (0.953;1.009)	0.029 (0.012;0.046)	0.716 (0.712;0.719)	0.956 (0.934;0.979)	0.02 (0.004;0.037)
DARTS	male	0.633 (0.629;0.637)	1.037 (0.926;1.148)	-0.002 (-0.017;0.012)	0.65 (0.646;0.654)	1.022 (0.929;1.114)	0.006 (-0.008;0.02)
	female	0.658 (0.654;0.663)	1.046 (0.972;1.121)	0.025 (0.007;0.042)	0.678 (0.673;0.682)	0.994 (0.937;1.052)	0.016 (-0.001;0.033)
Finrisk CHD	male	0.646 (0.642;0.649)	1.039 (0.984;1.094)	-0.001 (-0.016;0.013)	0.666 (0.662;0.669)	1.019 (0.973;1.065)	0.007 (-0.006;0.021)
	female	0.679 (0.674;0.683)	1.021 (0.965;1.076)	0.03 (0.013;0.048)	0.699 (0.694;0.703)	0.974 (0.928;1.021)	0.022 (0.005;0.039)
Finrisk CVD	male	0.668 (0.665;0.672)	1.038 (1;1.076)	-0.002 (-0.017;0.012)	0.69 (0.686;0.693)	1.015 (0.984;1.047)	0.006 (-0.008;0.02)
	female	0.684 (0.679;0.688)	1.016 (0.965;1.067)	0.031 (0.013;0.048)	0.704 (0.7;0.708)	0.972 (0.928;1.017)	0.023 (0.006;0.04)
Finrisk Stroke	male	0.679 (0.675;0.682)	1.04 (1.01;1.069)	-0.004 (-0.018;0.011)	0.701 (0.697;0.704)	1.016 (0.992;1.04)	0.005 (-0.009;0.019)
	female	0.689 (0.685;0.694)	1.007 (0.96;1.054)	0.032 (0.015;0.05)	0.71 (0.706;0.714)	0.969 (0.927;1.011)	0.025 (0.008;0.041)
Framingham 1998	male	0.669 (0.665;0.673)	1.041 (1.007;1.076)	-0.004 (-0.018;0.011)	0.69 (0.686;0.693)	1.021 (0.996;1.046)	0.005 (-0.009;0.02)
	female	0.689 (0.685;0.693)	1.027 (0.988;1.065)	0.034 (0.017;0.051)	0.712 (0.708;0.715)	0.985 (0.956;1.014)	0.027 (0.01;0.043)

QRISK 2	male	0.665 (0.661;0.668)	1.001 (0.949;1.053)	0.002 (-0.012;0.016)	0.681 (0.677;0.684)	0.969 (0.925;1.013)	0.011 (-0.003;0.025)
	female	0.687 (0.682;0.691)	1.006 (0.959;1.052)	0.031 (0.013;0.049)	0.707 (0.703;0.711)	0.966 (0.929;1.002)	0.023 (0.006;0.04)
QRISK 3	male	0.657 (0.653;0.661)	0.99 (0.938;1.043)	0.002 (-0.013;0.016)	0.674 (0.67;0.677)	0.966 (0.921;1.01)	0.011 (-0.003;0.025)
	female	0.684 (0.68;0.689)	1.011 (0.961;1.06)	0.03 (0.012;0.047)	0.704 (0.7;0.708)	0.967 (0.928;1.006)	0.021 (0.004;0.038)
RECODE	male	0.726 (0.722;0.73)	1.017 (0.985;1.049)	0 (-0.016;0.016)	0.728 (0.724;0.732)	1.007 (0.971;1.043)	0.01 (-0.006;0.025)
	female	0.73 (0.725;0.735)	0.994 (0.945;1.043)	0.034 (0.015;0.054)	0.735 (0.73;0.739)	0.982 (0.935;1.03)	0.025 (0.007;0.043)
Reynolds Risk	male	0.653 (0.65;0.657)	1.035 (0.988;1.082)	0 (-0.015;0.014)	0.673 (0.67;0.677)	1.019 (0.98;1.058)	0.008 (-0.006;0.022)
	female	0.66 (0.656;0.665)	1.039 (0.951;1.127)	0.03 (0.012;0.048)	0.679 (0.675;0.684)	0.987 (0.917;1.057)	0.022 (0.005;0.04)
SCORE CHD	male	0.654 (0.65;0.657)	1.043 (0.999;1.086)	0 (-0.015;0.014)	0.674 (0.671;0.678)	1.025 (0.989;1.061)	0.008 (-0.006;0.022)
	female	0.682 (0.678;0.686)	1.026 (0.985;1.066)	0.031 (0.013;0.048)	0.705 (0.701;0.709)	0.979 (0.946;1.012)	0.023 (0.007;0.039)
SCORE CVD	male	0.663 (0.659;0.667)	1.041 (1.002;1.081)	-0.001 (-0.016;0.013)	0.684 (0.681;0.688)	1.023 (0.991;1.055)	0.008 (-0.006;0.022)
	female	0.688 (0.683;0.692)	1.02 (0.983;1.057)	0.032 (0.015;0.049)	0.711 (0.707;0.715)	0.978 (0.948;1.008)	0.024 (0.008;0.04)
UKPDS 56	male	0.614 (0.609;0.618)	1.015 (0.915;1.115)	0 (-0.014;0.014)	0.63 (0.625;0.634)	1.017 (0.934;1.1)	0.008 (-0.005;0.022)
	female	0.642 (0.638;0.647)	1.042 (0.944;1.14)	0.028 (0.01;0.046)	0.66 (0.655;0.664)	0.988 (0.917;1.059)	0.019 (0.002;0.037)
UKPDS 68 C-HF	male	0.659 (0.655;0.663)	1.043 (0.998;1.089)	-0.001 (-0.016;0.014)	0.683 (0.679;0.687)	1.024 (0.984;1.064)	0.008 (-0.007;0.022)
	female	0.674 (0.669;0.678)	0.996 (0.961;1.031)	0.033 (0.015;0.05)	0.701 (0.697;0.705)	0.965 (0.934;0.996)	0.026 (0.009;0.042)
UKPDS 68 Stroke	male	0.659 (0.655;0.663)	1.043 (0.998;1.089)	-0.001 (-0.016;0.014)	0.683 (0.679;0.687)	1.024 (0.984;1.064)	0.008 (-0.007;0.022)
	female	0.674 (0.669;0.678)	0.996 (0.961;1.031)	0.033 (0.015;0.05)	0.701 (0.697;0.705)	0.965 (0.934;0.996)	0.026 (0.009;0.042)
UKPDS 82 C-HF	male	0.648 (0.644;0.653)	1.055 (0.997;1.113)	-0.001 (-0.016;0.014)	0.672 (0.668;0.677)	1.022 (0.975;1.07)	0.008 (-0.007;0.022)
	female	0.656 (0.651;0.662)	0.961 (0.918;1.004)	0.029 (0.011;0.047)	0.683 (0.678;0.687)	0.938 (0.902;0.974)	0.021 (0.003;0.038)
UKPDS 82 CHD	male	0.651 (0.647;0.656)	1.037 (0.977;1.096)	-0.001 (-0.016;0.013)	0.669 (0.665;0.673)	1.008 (0.954;1.061)	0.007 (-0.007;0.022)
	female	0.689 (0.683;0.695)	0.952 (0.915;0.99)	0.036 (0.018;0.053)	0.707 (0.702;0.713)	0.96 (0.925;0.996)	0.027 (0.01;0.043)

ESM Table 24 Discrimination (c-statistic) and calibration (calibration-in-the-large (CIL) and calibration slope (CS)) of the risk scores against the CVD and CVD + AF + HF outcomes for the imputed dataset (including all type 2 diabetes patients irrespective of CVD status at baseline) stratified by statin usage at baseline (statin naïve vs statin users).

Risk Score Name	Statins usage at baseline (statin naïve vs statin users)	CVD			CVD + AF + HF		
		C-stat	CS	CIL	C-stat	CS	CIL
ASCVD	statin naïve	0.692 (0.688;0.695)	1.022 (0.989;1.055)	0.026 (0.012;0.041)	0.713 (0.71;0.716)	1.011 (0.983;1.038)	0.025 (0.011;0.039)
	statin users	0.623 (0.619;0.628)	1.002 (0.949;1.054)	-0.024 (-0.042;-0.007)	0.643 (0.639;0.648)	0.955 (0.915;0.996)	-0.025 (-0.042;-0.008)
Framingham 1991 fatal CHD	statin naïve	0.659 (0.655;0.663)	1.107 (1.054;1.16)	0.028 (0.014;0.043)	0.672 (0.668;0.675)	1.06 (1.024;1.095)	0.026 (0.013;0.04)
	statin users	0.589 (0.584;0.594)	1.02 (0.923;1.118)	-0.02 (-0.038;-0.003)	0.598 (0.593;0.603)	1.006 (0.92;1.093)	-0.021 (-0.038;-0.004)
Framingham 1991 CVD	statin naïve	0.644 (0.64;0.649)	1.111 (1.056;1.167)	0.028 (0.013;0.042)	0.652 (0.647;0.656)	1.061 (1.023;1.099)	0.025 (0.012;0.039)
	statin users	0.593 (0.588;0.598)	1.039 (0.954;1.123)	-0.021 (-0.038;-0.003)	0.597 (0.592;0.602)	1.043 (0.967;1.119)	-0.021 (-0.038;-0.004)
Framingham 1991 Stroke	statin naïve	0.668 (0.663;0.672)	1.086 (1.039;1.132)	0.03 (0.016;0.045)	0.684 (0.68;0.688)	1.05 (1.018;1.083)	0.029 (0.015;0.043)
	statin users	0.602 (0.597;0.607)	1.027 (0.964;1.09)	-0.021 (-0.039;-0.004)	0.616 (0.612;0.621)	1.04 (0.989;1.09)	-0.022 (-0.039;-0.005)
CHS Advanced	statin naïve	0.693 (0.69;0.696)	1.031 (1.007;1.055)	0.025 (0.01;0.039)	0.72 (0.716;0.723)	1.02 (1;1.041)	0.022 (0.009;0.036)
	statin users	0.626 (0.622;0.631)	0.988 (0.949;1.028)	-0.021 (-0.038;-0.003)	0.649 (0.645;0.654)	0.95 (0.918;0.982)	-0.021 (-0.038;-0.004)
CHS Basic	statin naïve	0.693 (0.689;0.696)	1.033 (1.007;1.058)	0.024 (0.01;0.038)	0.719 (0.716;0.722)	1.021 (0.999;1.043)	0.021 (0.008;0.035)
	statin users	0.628 (0.624;0.633)	0.994 (0.954;1.033)	-0.021 (-0.038;-0.003)	0.651 (0.646;0.655)	0.955 (0.923;0.987)	-0.021 (-0.038;-0.003)
DARTS	statin naïve	0.671 (0.667;0.675)	1.063 (1.017;1.109)	0.026 (0.011;0.04)	0.689 (0.686;0.693)	1.048 (1.008;1.087)	0.024 (0.009;0.038)
	statin users	0.579 (0.573;0.584)	0.953 (0.862;1.045)	-0.026 (-0.044;-0.009)	0.594 (0.589;0.599)	0.896 (0.83;0.962)	-0.028 (-0.045;-0.011)
Finrisk CHD	statin naïve	0.688 (0.684;0.691)	1.062 (1.028;1.095)	0.027 (0.012;0.042)	0.707 (0.704;0.71)	1.04 (1.012;1.069)	0.026 (0.012;0.039)
	statin users	0.599 (0.594;0.604)	1.006 (0.943;1.07)	-0.024 (-0.041;-0.006)	0.617 (0.612;0.622)	0.961 (0.91;1.013)	-0.024 (-0.042;-0.007)
Finrisk CVD	statin naïve	0.696 (0.692;0.7)	1.045 (1.017;1.074)	0.028 (0.013;0.042)	0.716 (0.713;0.72)	1.024 (1.001;1.048)	0.026 (0.012;0.04)
	statin users	0.62 (0.616;0.625)	1.001 (0.954;1.048)	-0.02 (-0.038;-0.003)	0.639 (0.634;0.643)	0.956 (0.916;0.996)	-0.02 (-0.038;-0.003)
Finrisk Stroke	statin naïve	0.696 (0.692;0.7)	1.032 (1.006;1.059)	0.028 (0.013;0.042)	0.717 (0.714;0.721)	1.012 (0.99;1.034)	0.026 (0.012;0.04)
	statin users	0.632 (0.627;0.636)	1.001 (0.96;1.041)	-0.016 (-0.034;0.001)	0.649 (0.645;0.654)	0.954 (0.919;0.988)	-0.016 (-0.033;0.002)
Framingham 1998	statin naïve	0.691 (0.687;0.694)	1.039 (1.007;1.071)	0.029 (0.015;0.044)	0.715 (0.712;0.718)	1.022 (0.999;1.045)	0.028 (0.014;0.042)
	statin users	0.609 (0.605;0.614)	1 (0.952;1.048)	-0.022 (-0.04;-0.005)	0.632 (0.627;0.636)	0.994 (0.954;1.033)	-0.023 (-0.04;-0.006)
QRISK 2	statin naïve	0.687 (0.683;0.69)	1.041 (0.995;1.087)	0.027 (0.012;0.041)	0.707 (0.704;0.711)	1.022 (0.989;1.056)	0.025 (0.011;0.039)
	statin users	0.62 (0.615;0.625)	0.98 (0.927;1.033)	-0.023 (-0.04;-0.005)	0.636 (0.632;0.641)	0.963 (0.913;1.013)	-0.023 (-0.04;-0.005)
QRISK 3	statin naïve	0.685 (0.681;0.689)	1.04 (0.997;1.083)	0.027 (0.012;0.042)	0.705 (0.702;0.708)	1.017 (0.985;1.049)	0.025 (0.011;0.04)

	statin users	0.613 (0.608;0.618)	0.993 (0.935;1.05)	-0.022 (-0.039;-0.004)	0.63 (0.625;0.634)	0.957 (0.902;1.012)	-0.022 (-0.039;-0.004)
RECODE	statin naïve	0.709 (0.704;0.713)	1.077 (1.033;1.121)	0.026 (0.011;0.041)	0.717 (0.713;0.721)	1.066 (1.031;1.101)	0.023 (0.009;0.037)
	statin users	0.743 (0.738;0.747)	0.992 (0.955;1.029)	-0.011 (-0.031;0.009)	0.74 (0.735;0.744)	0.951 (0.917;0.985)	-0.012 (-0.031;0.008)
Reynolds Risk	statin naïve	0.685 (0.681;0.688)	1.057 (1.019;1.094)	0.028 (0.013;0.043)	0.702 (0.699;0.705)	1.034 (1.004;1.065)	0.026 (0.012;0.04)
	statin users	0.602 (0.597;0.607)	1.01 (0.944;1.077)	-0.022 (-0.04;-0.005)	0.617 (0.612;0.622)	0.966 (0.914;1.019)	-0.023 (-0.04;-0.005)
SCORE CHD	statin naïve	0.694 (0.691;0.698)	1.066 (1.035;1.097)	0.027 (0.012;0.041)	0.714 (0.71;0.717)	1.043 (1.019;1.066)	0.025 (0.011;0.038)
	statin users	0.607 (0.603;0.612)	1.028 (0.977;1.08)	-0.024 (-0.041;-0.006)	0.625 (0.62;0.63)	0.967 (0.927;1.008)	-0.024 (-0.041;-0.007)
SCORE CVD	statin naïve	0.698 (0.694;0.701)	1.056 (1.029;1.083)	0.028 (0.013;0.042)	0.719 (0.716;0.722)	1.036 (1.014;1.057)	0.026 (0.013;0.04)
	statin users	0.614 (0.609;0.619)	1.015 (0.968;1.062)	-0.023 (-0.041;-0.006)	0.634 (0.63;0.639)	0.963 (0.926;1.001)	-0.024 (-0.041;-0.006)
UKPDS 56	statin naïve	0.659 (0.655;0.664)	1.079 (1.025;1.134)	0.024 (0.01;0.039)	0.67 (0.666;0.673)	1.058 (1.01;1.106)	0.022 (0.008;0.035)
	statin users	0.584 (0.578;0.59)	1.002 (0.913;1.091)	-0.023 (-0.04;-0.006)	0.591 (0.586;0.597)	0.929 (0.849;1.009)	-0.023 (-0.04;-0.006)
UKPDS 68 C-HF	statin naïve	0.671 (0.668;0.675)	1.04 (0.998;1.082)	0.026 (0.012;0.041)	0.7 (0.697;0.704)	1.031 (0.997;1.064)	0.025 (0.011;0.039)
	statin users	0.598 (0.593;0.603)	0.933 (0.867;0.998)	-0.021 (-0.039;-0.003)	0.625 (0.62;0.629)	0.923 (0.879;0.968)	-0.021 (-0.038;-0.003)
UKPDS 68 Stroke	statin naïve	0.671 (0.668;0.675)	1.04 (0.998;1.082)	0.026 (0.012;0.041)	0.7 (0.697;0.704)	1.031 (0.997;1.064)	0.025 (0.011;0.039)
	statin users	0.598 (0.593;0.603)	0.933 (0.867;0.998)	-0.021 (-0.039;-0.003)	0.625 (0.62;0.629)	0.923 (0.879;0.968)	-0.021 (-0.038;-0.003)
UKPDS 82 C-HF	statin naïve	0.658 (0.653;0.663)	1.021 (0.981;1.061)	0.022 (0.007;0.037)	0.687 (0.683;0.691)	1.013 (0.977;1.048)	0.019 (0.005;0.034)
	statin users	0.588 (0.583;0.593)	0.89 (0.803;0.977)	-0.021 (-0.038;-0.003)	0.614 (0.609;0.619)	0.911 (0.847;0.976)	-0.021 (-0.038;-0.003)
UKPDS 82 CHD	statin naïve	0.648 (0.643;0.653)	1.032 (0.978;1.086)	0.026 (0.012;0.04)	0.671 (0.666;0.675)	1.036 (0.993;1.079)	0.024 (0.01;0.037)
	statin users	0.586 (0.58;0.591)	1.011 (0.93;1.091)	-0.024 (-0.042;-0.007)	0.605 (0.6;0.61)	1.024 (0.955;1.094)	-0.026 (-0.043;-0.008)

ESM Figures

ESM Figure 1 Flow diagram of the selection of articles for the literature review.

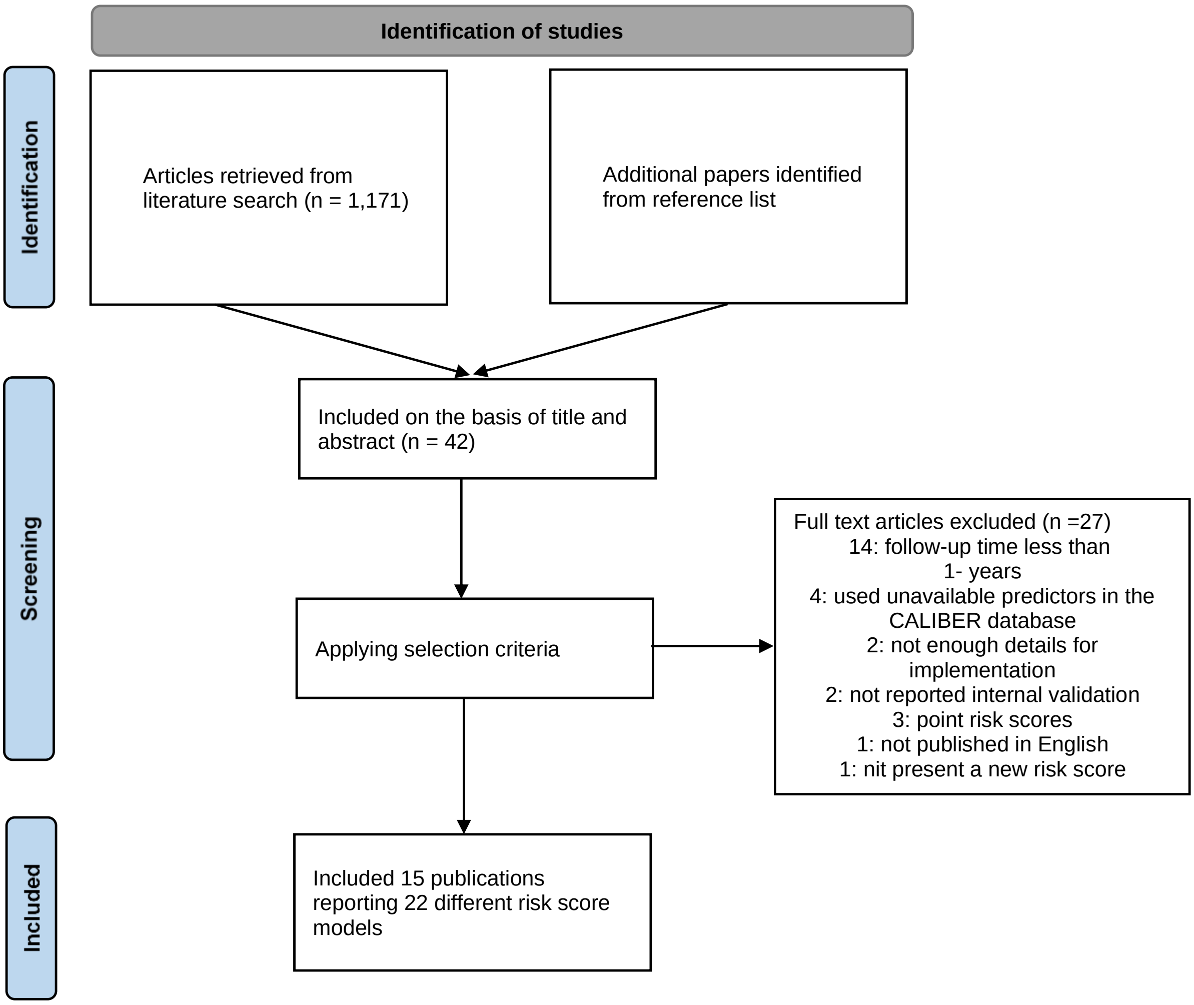

ESM Figure 2 The maximum number of predictors used by the considered CVD risk scores

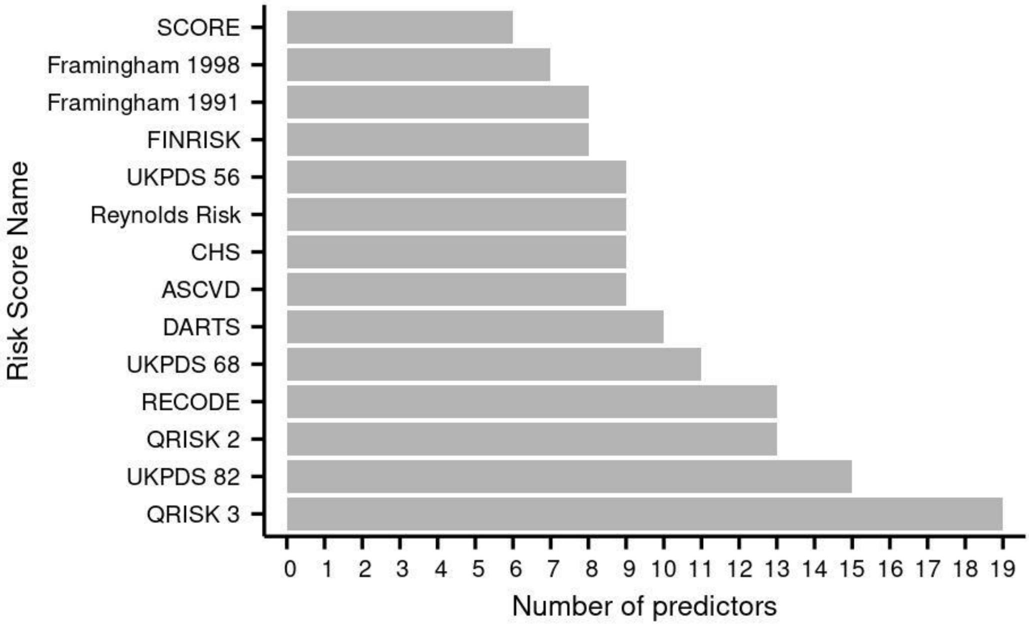

Calibration based on a complete-case analysis

ESM Figure 3: Calibration plots of the prediction rules for 10-year CVD risk, applied to patients with type 2 diabetes. Estimates are based on a complete-case analysis. Scores were evaluated against CVD, CHD, and CVD + AF + HF.

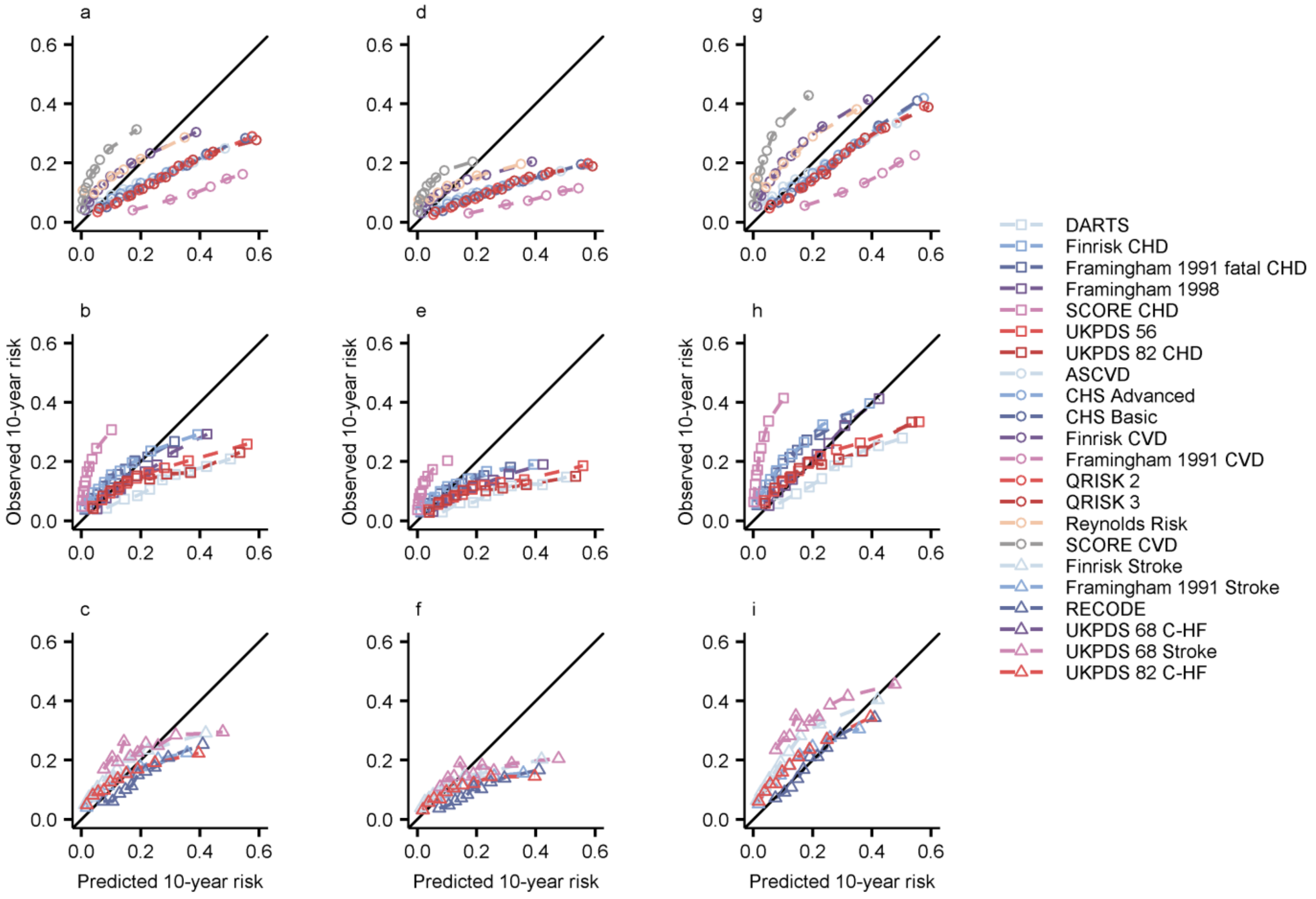

n.b. Estimates are based on complete cases of patients with type 2 diabetes. The performance depicted is based on 90% of the data (used for external validation). The observed 10-year risk (y-axis) is plotted against the mean predicted 10-year risk (x-axis) within groups defined by quintiles of predicted risk. The columns indicate the type of CVD the scores were evaluated against (a – c against CVD, d – f against CHD, g – i against CVD + AF + HF). Scores were grouped by the derivation outcomes CVD (subplots a, d, g), CHD (subplots b, e, h), or other (including Stroke, C-HF)) (subplots c, f, i). The diagonal line reflects perfect calibration.

ESM Figure 4: Calibration plots of the prediction rules for 10-year CVD risk, applied to patients with type 2 diabetes. Estimates are based on a complete-case analysis. Scores were evaluated against any Stroke, AF, and HF.

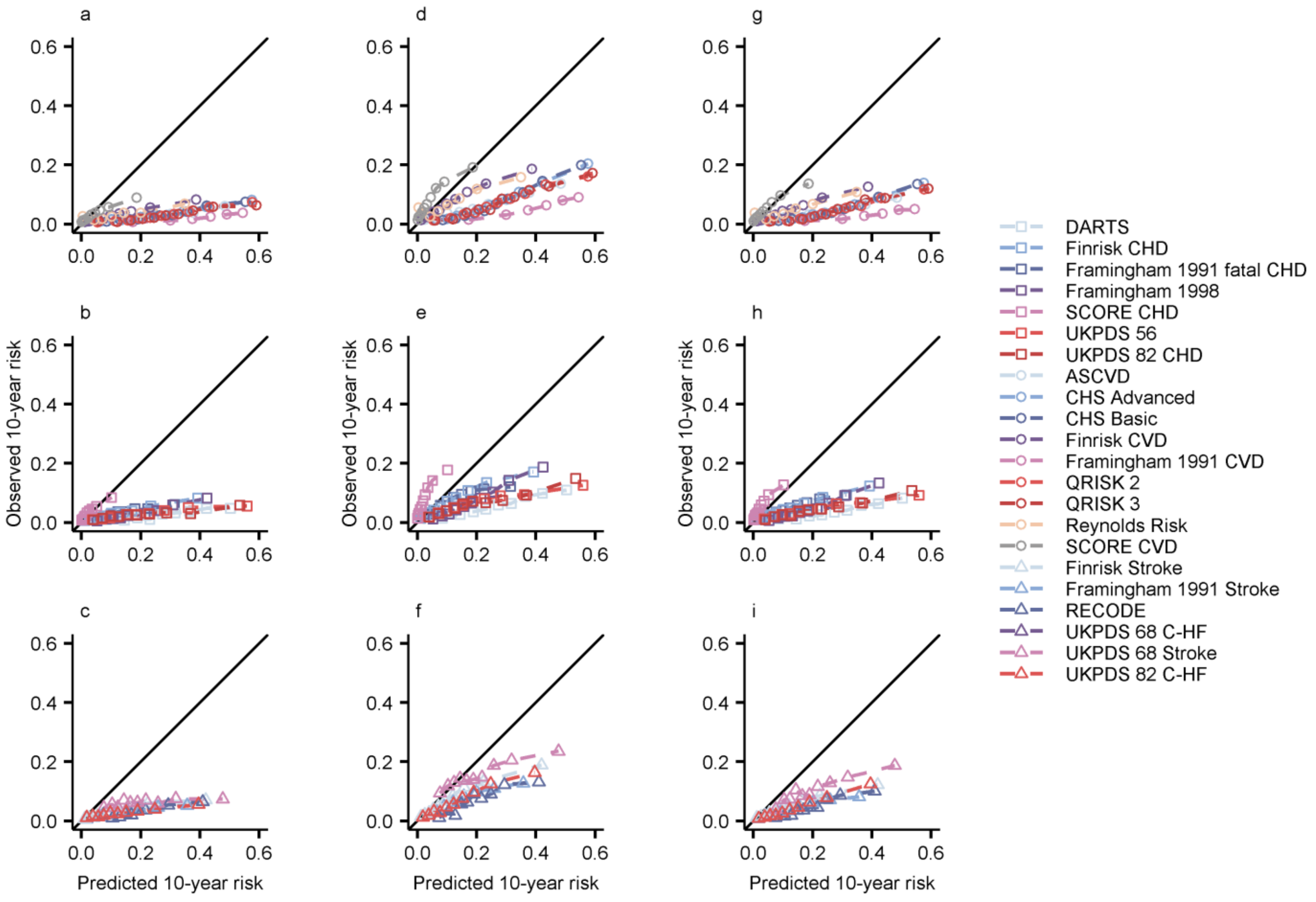

n.b. Estimates are based on complete cases of patients with type 2 diabetes. The performance depicted is based on 90% of the data (used for external validation). The observed 10-years risk (y-axis) is plotted against the mean predicted 10-year risk (x-axis) within groups defined by quintiles of predicted risk. The columns indicate the type of CVD the scores were evaluated against (a – c against Stroke, d -f against AF, g – i against HF). Scores were grouped by the derivation outcomes CVD (subplots a, d, g), CHD (subplots b, e, h), or other (including Stroke, C-HF)) (subplots c, f, i). The diagonal line reflects perfect calibration.

Calibration based on a multiple imputation analysis.

ESM Figure 5: Calibration plots of the prediction rules for 10-year CVD risk, applied to patients with type 2 diabetes. Estimates based on imputed data. Scores were evaluated against CVD, CHD, and CVD + AF + HF.

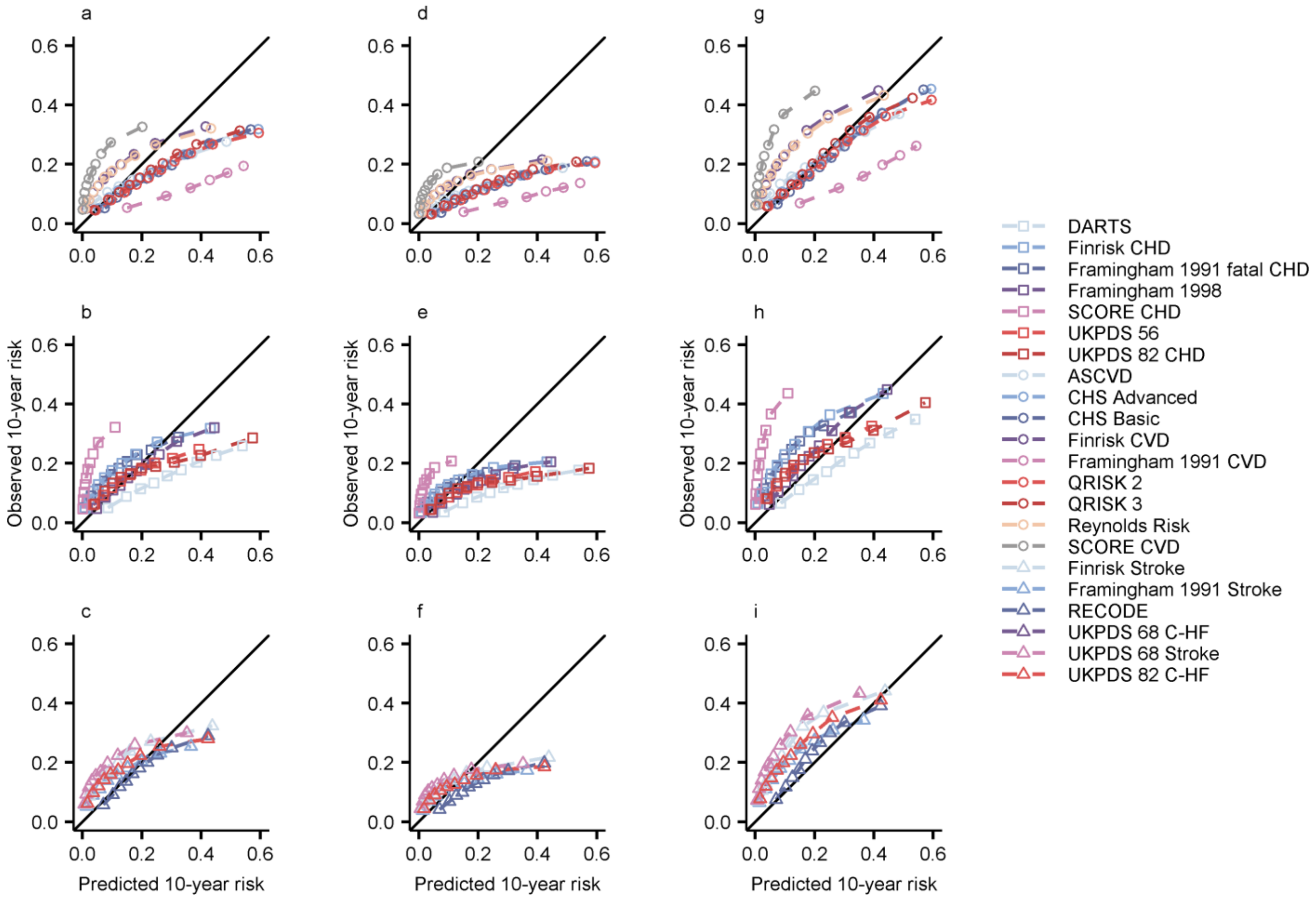

n.b. Estimates are based on imputed data. The observed 10-year (y-axis) risk is plotted against the mean predicted 10-year risk (x-axis) within groups defined by quintiles of predicted risk. The columns indicate the type of CVD the scores were evaluated against (a – c against CVD, d – f against CHD, g – i against CVD + AF + HF). Scores were grouped by the derivation outcomes CVD (subplots a, d, g), CHD (subplots b, e, h), or other (including Stroke, C-HF)) (subplots c, f, i). The diagonal line reflects perfect calibration.

ESM Figure 6: **Calibration plots of prediction rules for 10-years CVD risk, applied to patients with type 2 diabetes. Estimates based on imputed data. Scores were evaluated against any Stroke, AF, and HF.**

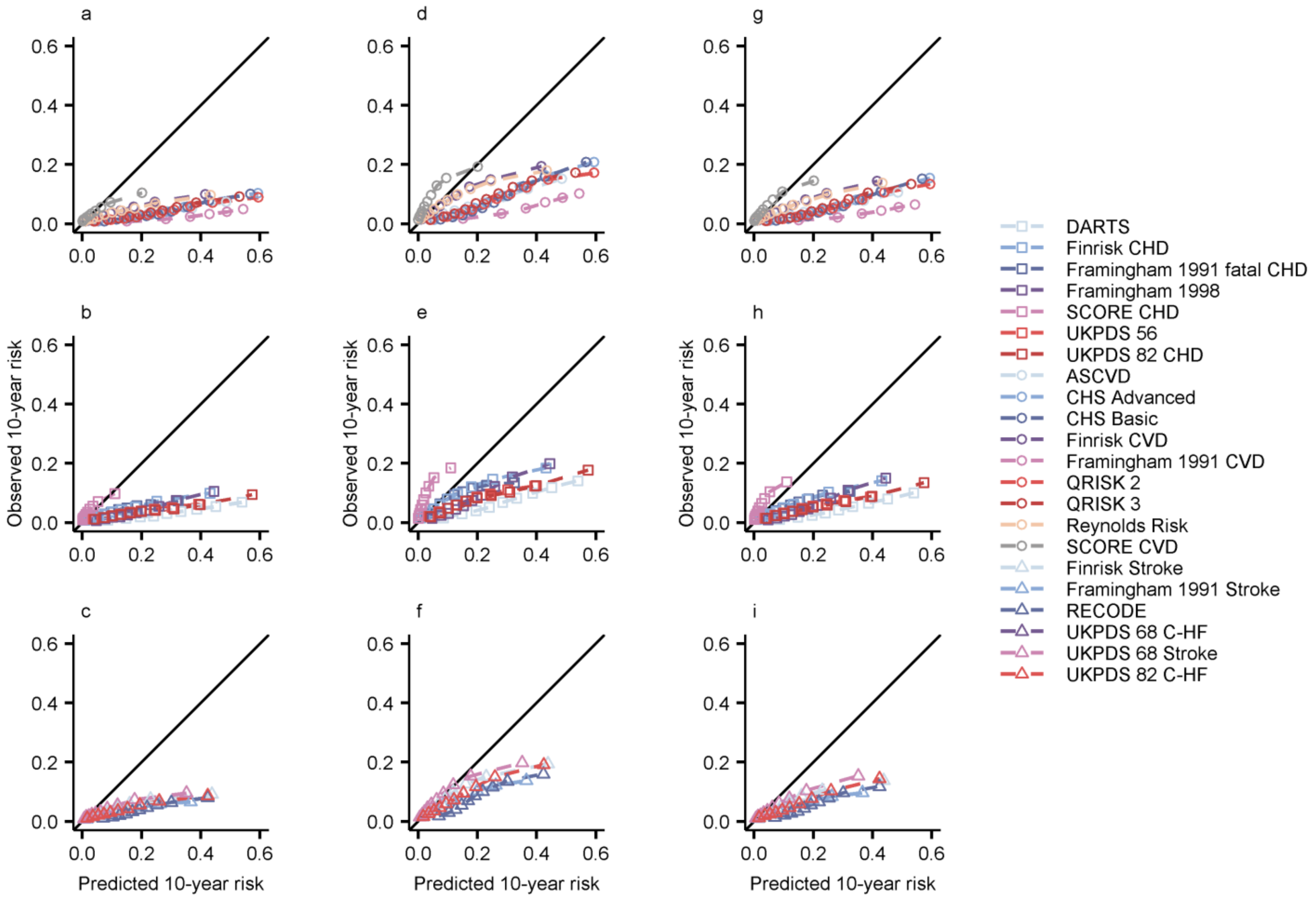

n.b. Estimates are based on imputed data. The performance depicted is based on 90% of the data used for external validation. The observed 10-year risk (y-axes) is plotted against the mean predicted 10-year risk (x-axis) within groups defined by quintiles of predicted risk. The columns indicate the type of CVD the scores were evaluated against (a – c against Stroke, d -f against AF, g – i against HF). Scores were grouped by the derivation outcomes CVD (subplots a, d, g), CHD (subplots b, e, h), or other (including Stroke, C-HF)) (subplots c, f, i). The diagonal line reflects perfect calibration.

ESM Figure 7 Interaction test p-value for the pairwise difference in c-statistic for CVD.

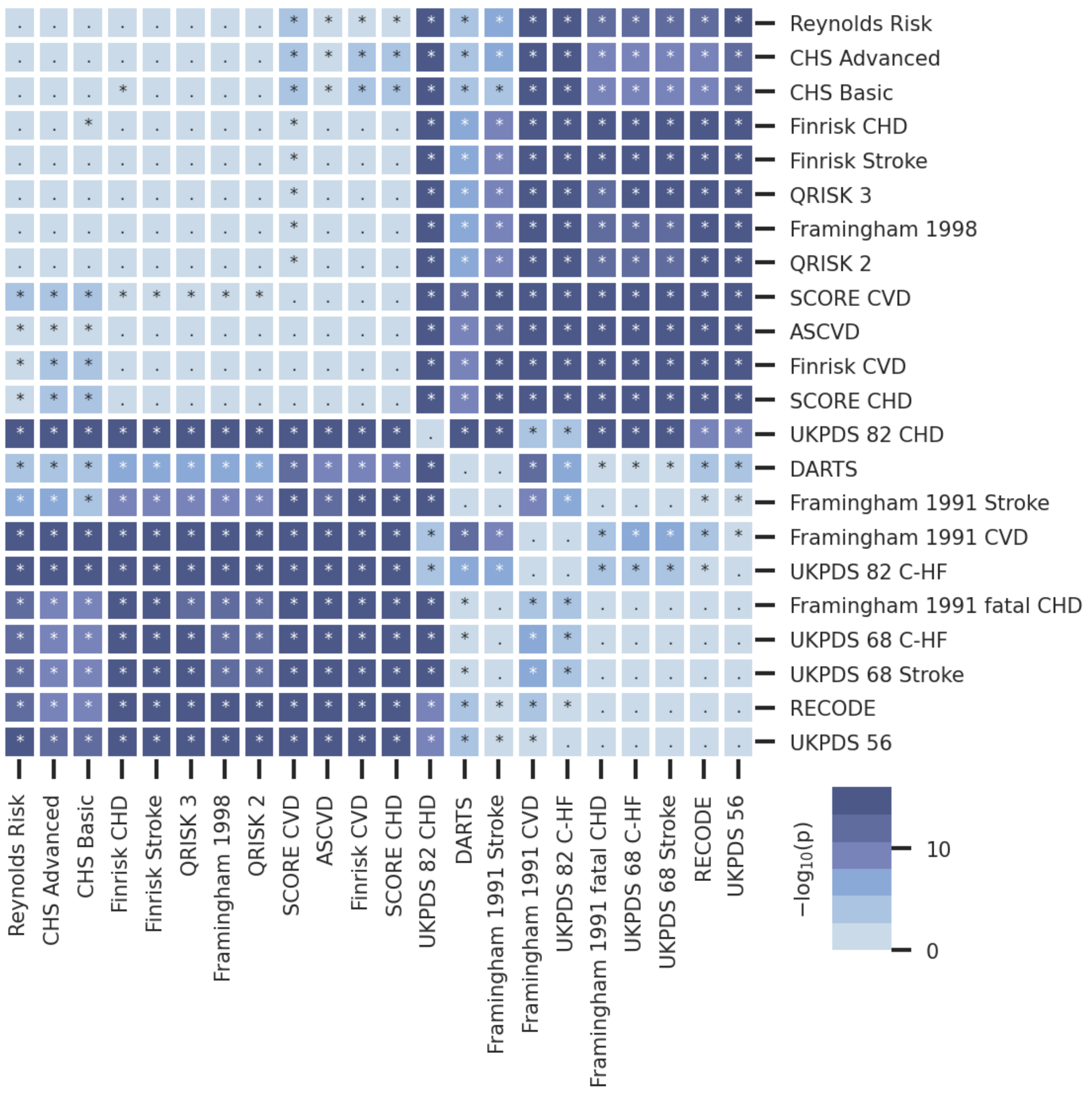

n.b. Stars inside a cell indicates significant difference (at an alpha of 0.05), dots indicate non-significant difference. Results are based on diabetes patients without pre-existing CVD at the time of diagnosis**.**

Discrimination estimates based on a complete-case analysis.

ESM Figure 8: C-statistic (discrimination) of 22 CVD risk prediction tools externally validated in a UK-based type 2 diabetes sample against CVD, CHD and CVD + AF + HF outcomes, split by the reported type of CVD outcome.

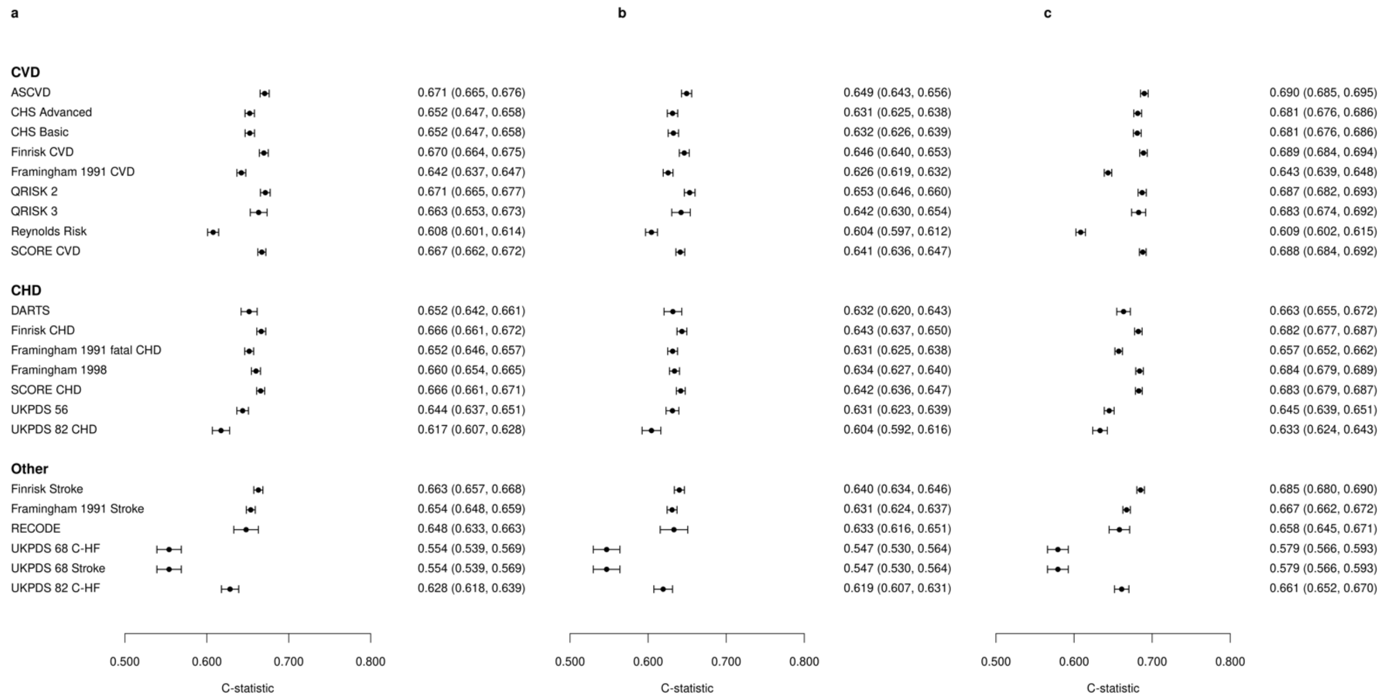

n.b. Point estimates are presented alongside 95% CI. Results were based on a complete-case analysis and based on 90% of the data used for external validation. The columns indicate the type of CVD the scores were evaluated against (a – against CVD, b – against CHD, c – against CVD + AF + HF).

ESM Figure 9: C-statistic (discrimination) of 22 CVD risk prediction tools externally validated in a UK-based type 2 diabetes sample against Stroke, AF and HF outcomes, split by the reported type of CVD outcome.

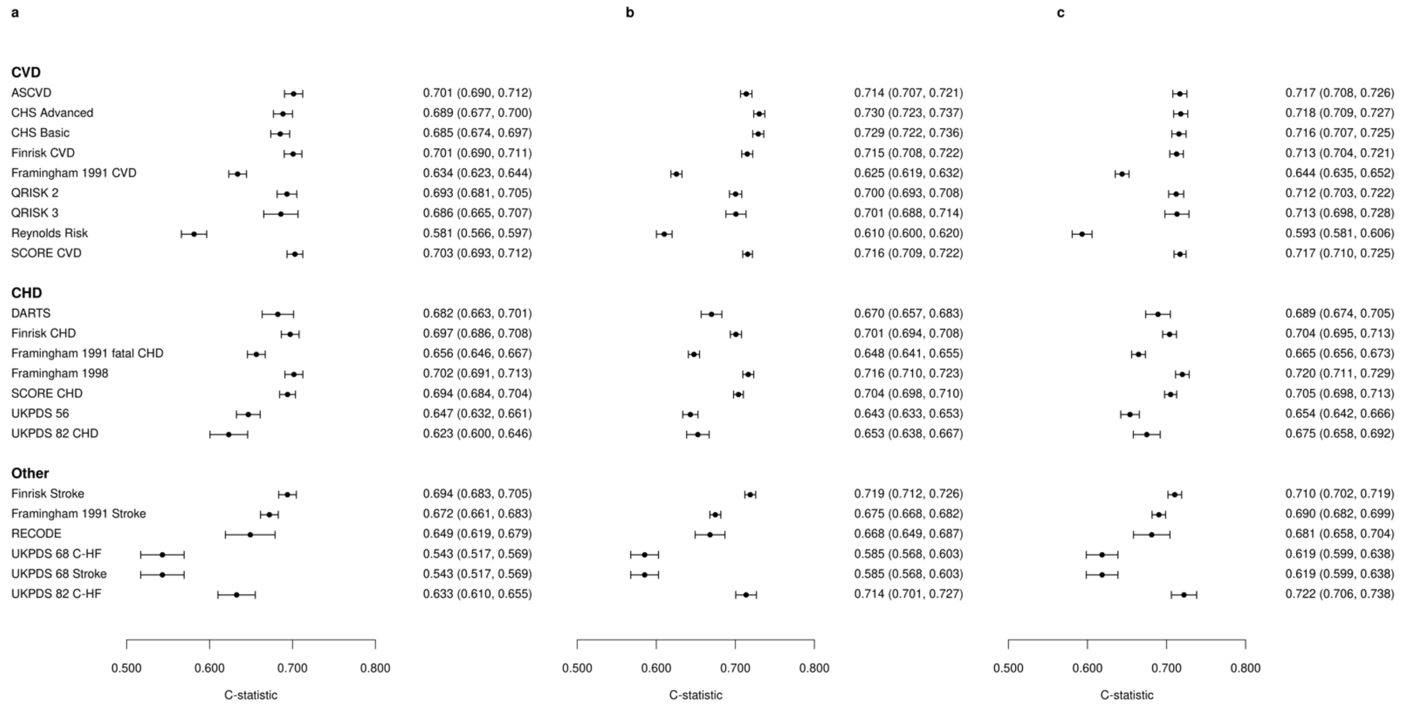

n.b. Point estimates are presented alongside 95% CI. Results were based on a complete-case analysis and based on 90% of the data used for external validation. The columns indicate the type of CVD the scores were evaluated against (a – against Stroke, b – against AF, c – against HF).

Discrimination estimates based on a multiple imputation analysis.

ESM Figure 10: C-statistic (discrimination) of 22 CVD risk prediction tools externally validated in a UK-based type 2 diabetes sample against CVD, CHD, and CVD + AF + HF outcomes, split by the reported type of CVD outcome.

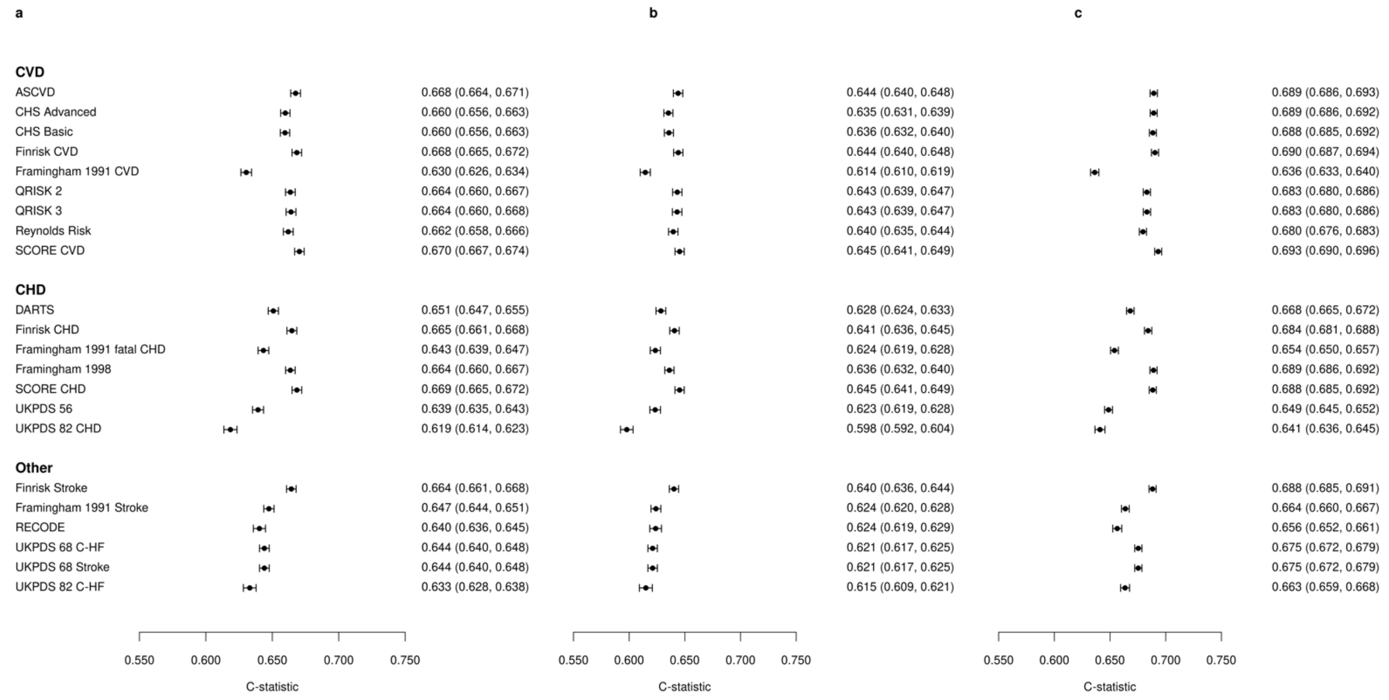

n.b. Point estimates are presented alongside 95% CI. Results were based on imputed data and based on 90% of the data used for external validation. The columns indicate the type of CVD the scores were evaluated against (a – against CVD, b – against CHD, c – against CVD + AF + HF).

ESM Figure 11: C-statistic (discrimination) of 22 CVD risk prediction tools externally validated in a UK-based type 2 diabetes sample against Stroke, AF and HF outcomes, split by the reported type of CVD outcome.

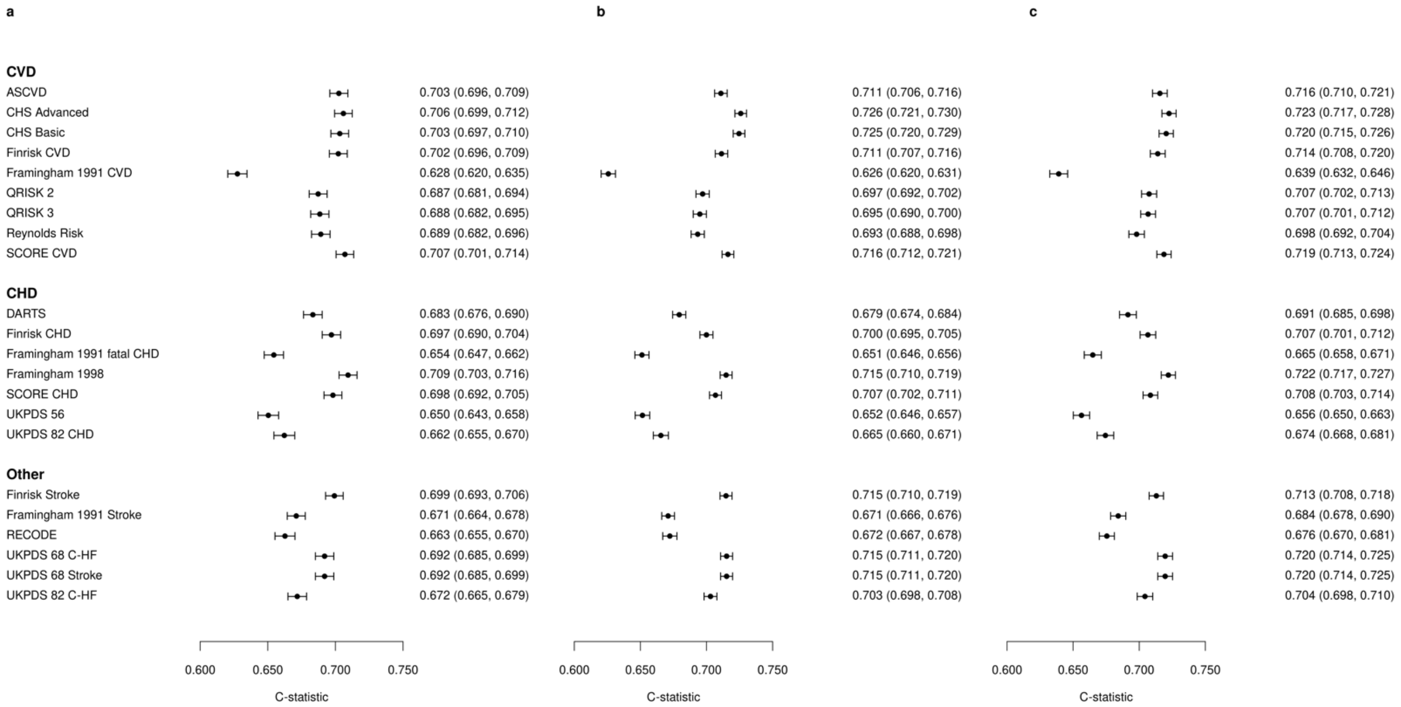

n.b. Point estimates are presented alongside 95%CI. Results were based on imputed data and based on 90% of the data used for external validation. The columns indicate the type of CVD the scores were evaluated against (a – against Stroke, b – against AF, c – against HF).

Performance after recalibration based on a complete-case analysis.

ESM Figure 12: Calibration plots for CVD, CHD, CVD+AF+HF after recalibration based on a complete-case analysis.

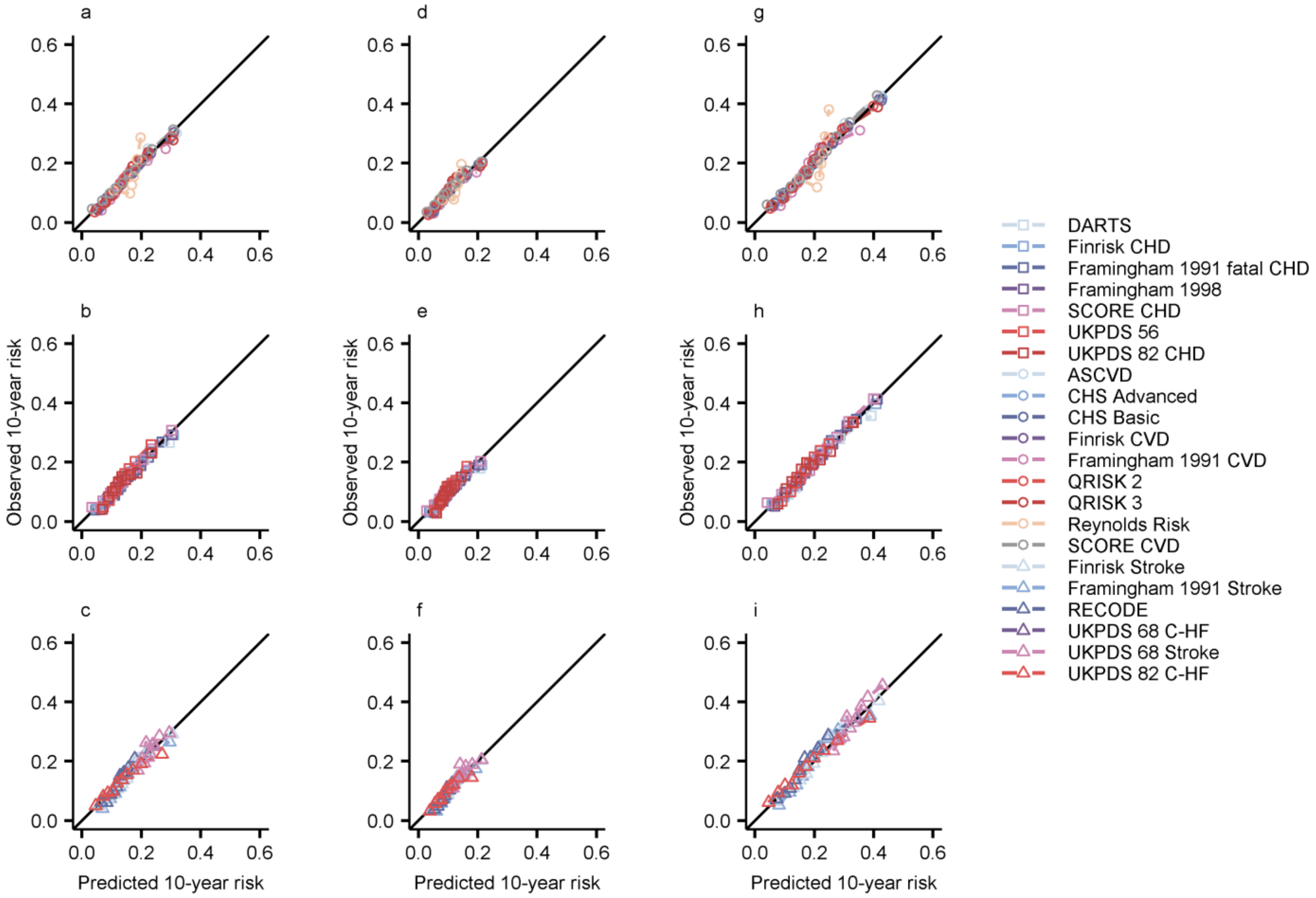

n.b. Estimates based on a complete-case analysis. The observed 10-year risk (y-axis) is plotted against the mean predicted 10-year risk (x-axis) within groups defined by quintiles of predicted risk. The columns indicate the type of CVD the scores were evaluated against (a – c against CVD, d – f against CHD, g – i against CVD + AF + HF). Scores were grouped by the derivation outcomes CVD (subplots a, d, g), CHD (subplots b, e, h), or other (including Stroke, C-HF)) (subplots c, f, i). The diagonal line reflects perfect calibration.

ESM Figure 13: Calibration plots for any stroke, AF, and HF, after recalibration based on a complete-case analysis.

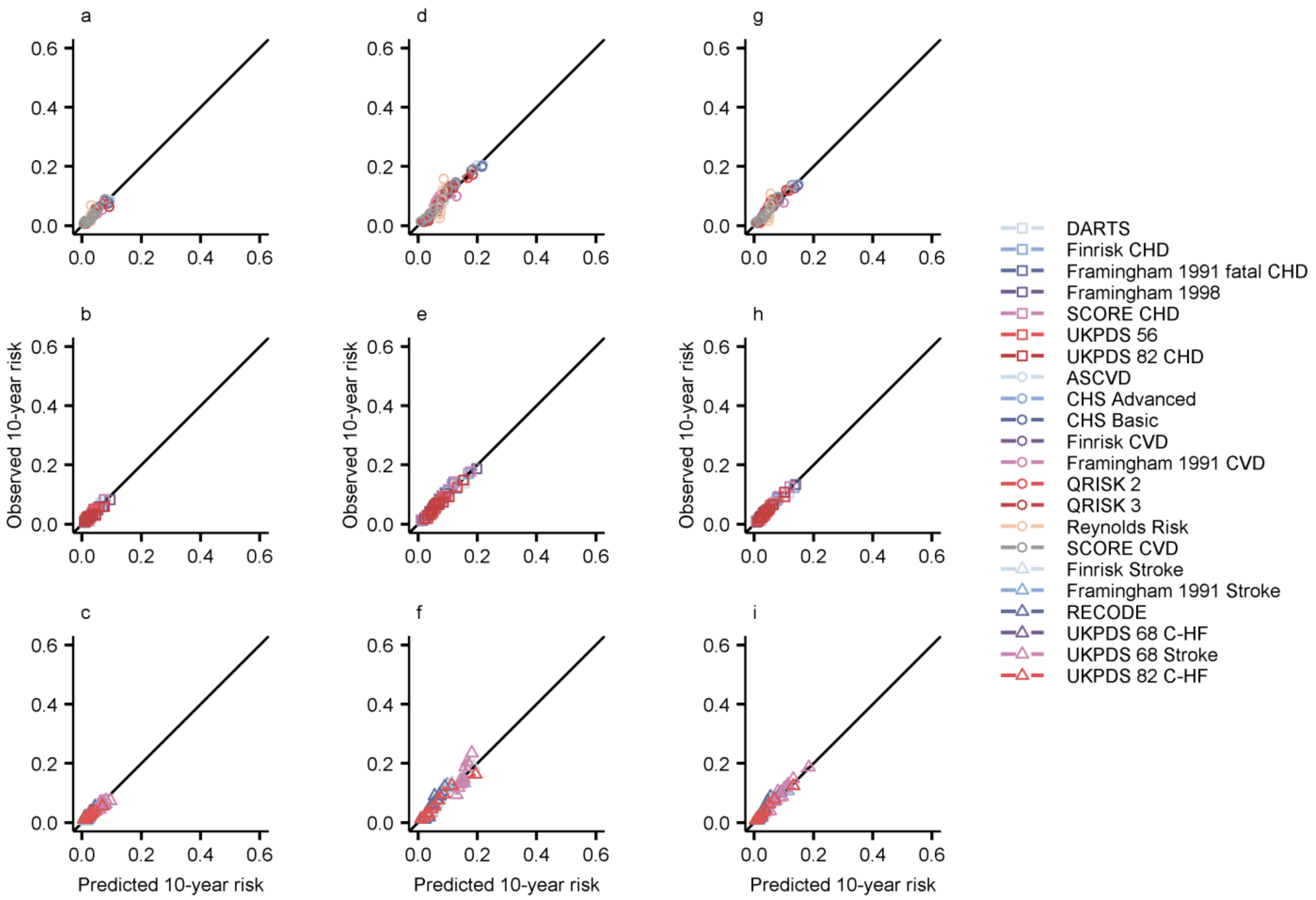

n.b. Estimates based on complete-case analysis. The observed 10-years risk is (y-axis) plotted against the mean predicted 10-year risk (x-axis) within groups defined by quintiles of predicted risk. The columns indicate the type of CVD the scores were evaluated against (a – c against Stroke, d – f against AF, g – i against HF). Scores were grouped by the derivation outcomes CVD, CHD, or other (including Stroke, C-HF)). Scores were grouped by the derivation outcomes CVD (subplots a, d, g), CHD (subplots b, e, h), or other (including Stroke, C-HF)) (subplots c, f, i). The diagonal line reflects perfect calibration.

Performance after recalibration based on a multiple imputation analysis.

ESM Figure 14: Calibration plots for CVD, CHD, and CVD+AF+HF, after recalibration of the CVD prediction rules calculated using the imputed datasets.

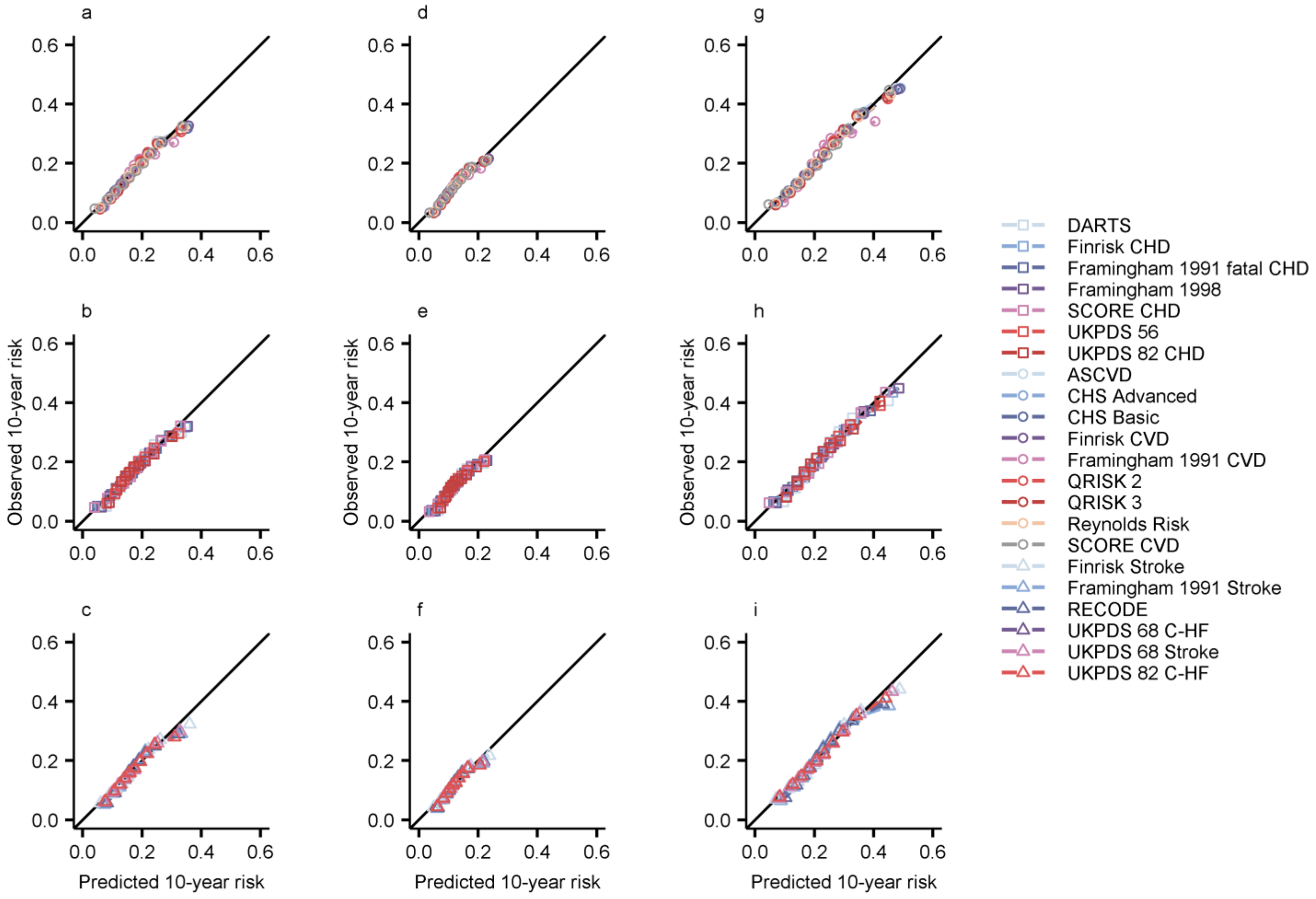

n.b. Estimates based on imputed data. The observed 10-year risk (y-axis) is plotted against the mean predicted 10-year risk (x-axis) within groups defined by quintiles of predicted risk. The columns indicate the type of CVD the scores were evaluated against (a – c against CVD, d – f against CHD, g – i against CVD + AF + HF). Scores were grouped by the derivation outcomes CVD (subplots a, d, g), CHD (subplots b, e, h), or other (including Stroke, C-HF)) (subplots c, f, i). The diagonal line reflects perfect calibration.

ESM Figure 15 Subgroup specific discrimination for CVD using imputed data for type 2 diabetes without pre-existing CVD+.

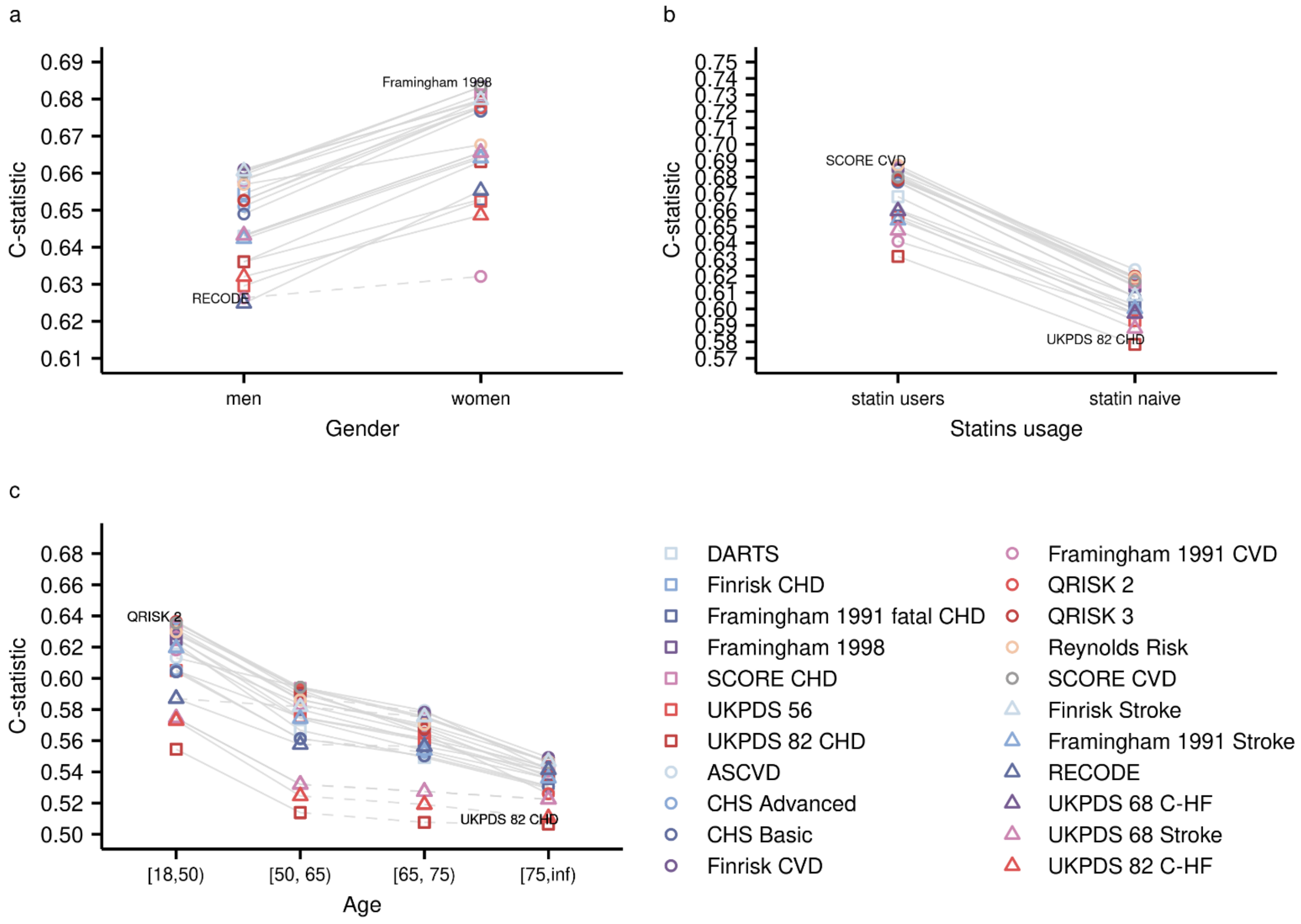

n.b. Results were based on 90% of the imputed data (for external validation) for type 2 diabetes patients without pre-existing CVD+. Plots stratified by a) sex (gender), b) statin usage, and c) age. Line type indicates the results of the interaction test for the difference in the c-statistics: a solid line refers to a statistically significant difference (p-value is less than 0.05) and a dashed line equates to a non-statistically significant difference (p-value is greater than 0.05).

ESM Figure 16 Subgroup specific discrimination for CVD using imputed data for all type 2 diabetes patients irrespective of their baseline CVD status.

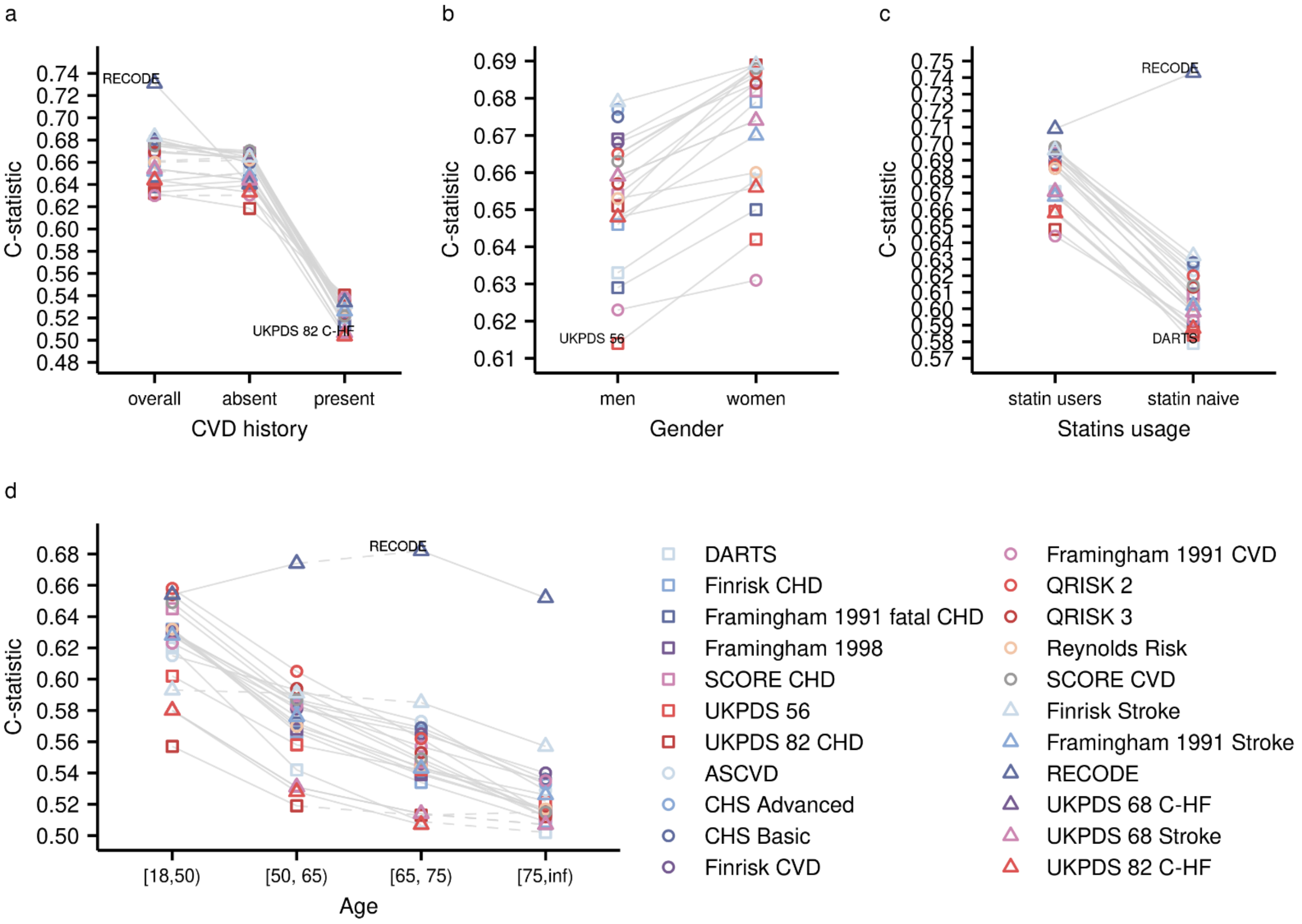

n.b. Results were based on 90% of the imputed data (for external validation) for type 2 diabetes patients irrespective of their baseline CVD status. Plots are stratified by a) CVD history at baseline, b) sex (gender), c) statin usage and d) age. Line type indicates the results of the interaction test for the difference in the c-statistics: a solid line refers to a statistically significant difference (p-value is less than 0.05) and a dashed line equates to a non-statistically significant difference (p-value is greater than 0.05).
